# Repression of Divergent Noncoding Transcription by a Sequence-Specific Transcription Factor

**DOI:** 10.1016/j.molcel.2018.10.018

**Published:** 2018-12-20

**Authors:** Andrew C.K. Wu, Harshil Patel, Minghao Chia, Fabien Moretto, David Frith, Ambrosius P. Snijders, Folkert J. van Werven

**Affiliations:** 1 Cell Fate and Gene Regulation Laboratory, The Francis Crick Institute, 1 Midland Road, London NW1 1AT, UK; 2 Bioinformatics and Biostatistics, The Francis Crick Institute, 1 Midland Road, London NW1 1AT, UK; 3 Protein Analysis and Proteomics Platform, The Francis Crick Institute, 1 Midland Road, London NW1 1AT, UK

**Keywords:** yeast, RSC, promoter, Rap1, transcription, divergent, noncoding RNA, transcription factor, repression, directionality

## Abstract

Many active eukaryotic gene promoters exhibit divergent noncoding transcription, but the mechanisms restricting expression of these transcripts are not well understood. Here, we demonstrate how a sequence-specific transcription factor represses divergent noncoding transcription at highly expressed genes in yeast. We find that depletion of the transcription factor Rap1 induces noncoding transcription in a large fraction of Rap1-regulated gene promoters. Specifically, Rap1 prevents transcription initiation at cryptic promoters near its binding sites, which is uncoupled from transcription regulation in the protein-coding direction. We further provide evidence that Rap1 acts independently of previously described chromatin-based mechanisms to repress cryptic or divergent transcription. Finally, we show that divergent transcription in the absence of Rap1 is elicited by the RSC chromatin remodeler. We propose that a sequence-specific transcription factor limits access of basal transcription machinery to regulatory elements and adjacent sequences that act as divergent cryptic promoters, thereby providing directionality toward productive transcription.

## Highlights

• Expression of divergent noncoding RNAs is repressed by Rap1
• Rap1 prevents initiation of divergent noncoding transcription near its binding sites
• Rap1 provides directionality toward productive transcription

## Graphical Abstract

**Figure:**
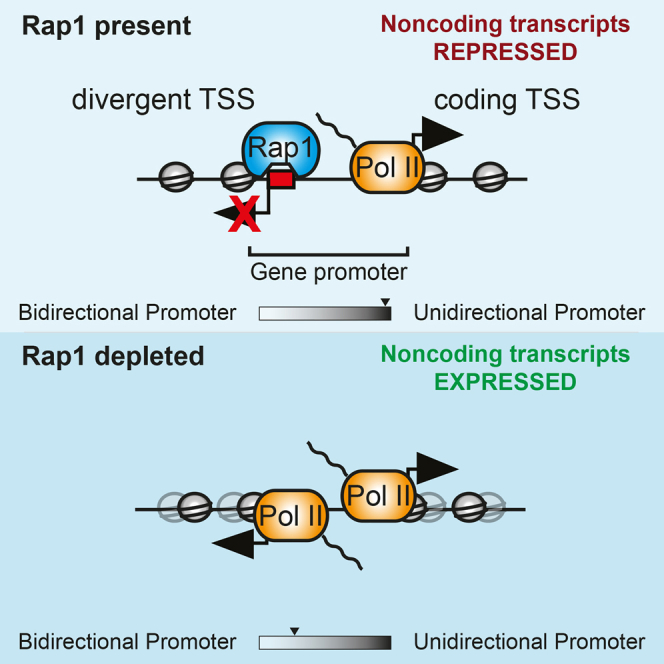

## Teaser

Wu et al. characterize how divergent noncoding RNAs are repressed at highly expressed gene promoters in yeast. The authors identified that the sequence-specific transcription factor Rap1 restricts transcription initiation in the divergent direction locally near its binding site, thereby controlling promoter directionality.

## Introduction

Precise control of gene expression is critical for all cellular functions. How and when genomes produce coding messenger RNAs and prevent the expression of unwanted RNAs has been a long-standing question of interest. In this context, an apparent paradox exists: genomic locations of coding gene transcription also produce aberrant noncoding transcripts. The transcriptionally active coding gene promoters, which often express noncoding transcripts in the antisense direction (Neil et al., 2009, Seila et al., 2008, Xu et al., 2009), are a major source. This process is known as divergent or bidirectional transcription. The functions of the noncoding RNAs produced and the mechanisms that limit expression of divergent noncoding transcripts are not well understood.

Divergent noncoding transcription is present across eukaryotic species. A large fraction of all noncoding transcripts emanate from divergent or bidirectional gene promoters (Neil et al., 2009, Seila et al., 2008, Xu et al., 2009). Typically, divergent noncoding transcripts initiate within or nearby coding gene promoters, but they do not share the same core promoter as transcripts in the coding direction (Andersson et al., 2015, Duttke et al., 2015, Rhee and Pugh, 2012, Scruggs et al., 2015). The transcription of divergent noncoding RNAs is lower than coding genes (Churchman and Weissman, 2011). Divergent noncoding transcripts are unstable and rapidly degraded. The Nrd1-Nab3-Sen1 and premature polyadenylation signal pathways in yeast and mammalian cells, respectively, terminate and degrade divergent transcripts (Jensen et al., 2013). In addition, exosome and nonsense-mediated decay pathways degrade cryptic and divergent transcripts (Neil et al., 2009, van Dijk et al., 2011). Divergent and pervasive transcription can also be repressed by controlling TATA-binding protein activity (Xue et al., 2017). Finally, CAF-1-mediated chromatin assembly represses the accumulation of divergent noncoding transcripts at promoters, which in turn is opposed by chromatin regulators that promote rapid turnover of nucleosomes (Marquardt et al., 2014).

In budding yeast, 138 genes encode for the protein subunits of the ribosome. These highly expressed ribosomal protein (RP) genes account for approximately half of all RNA polymerase II transcription (Warner, 1999). Transcription of nearly all RP genes is controlled by the pioneer transcription factor Rap1, which binds to upstream elements in RP gene promoters (Lieb et al., 2001). When RP promoters are active, Rap1 recruits coactivators such as Fhl1, Ifh1, and Sfp1, as well as basal transcription factors like TFIID and TFIIA (Azad and Tomar, 2016, Hu and Li, 2007). Thus, Rap1 orchestrates RP gene expression. Given that RP genes are among the most actively transcribed genes in yeast, they are an ideal model for studying how aberrant transcription is controlled.

Here, we describe how divergent noncoding transcription is repressed at highly active RP gene promoters. We find that depletion of Rap1, but not other transcription factors important for RP expression, causes transcription in the divergent direction. Rap1 represses noncoding transcription typically within 50 bp of the Rap1 motif, which is uncoupled from transcription regulation in the protein-coding direction. We further show that Rap1-mediated repression of divergent transcription is distinct from known chromatin-based mechanisms. Thus, a sequence-specific transcription factor controls promoter directionality by repressing transcription in the divergent direction. Our work adds a new layer of regulation to various mechanisms that limit expression of aberrant transcripts and defines how promoter directionality is controlled.

## Results

### Depletion of Rap1 Causes Divergent Transcription at *RPL43B* and *RPL40B*

In budding yeast, a large fraction of bidirectional promoters express noncoding transcripts, also known as cryptic unstable transcripts (CUTs) or stable unannotated transcripts (SUTs), in the divergent direction (Neil et al., 2009, Xu et al., 2009). Transcription of divergent CUTs and SUTs typically correlates with nucleosome-depleted regions (NDRs) and promoter activity in the coding gene direction. Considering that RP genes are among the most highly expressed genes in yeast, surprisingly few RP gene promoters (16 out of 138 promoters) display an annotated divergent noncoding transcript (CUT or SUT) (Neil et al., 2009, Xu et al., 2009). We hypothesized that RP promoters must have a robust mechanism for limiting divergent noncoding transcription.

**Figure 1:**
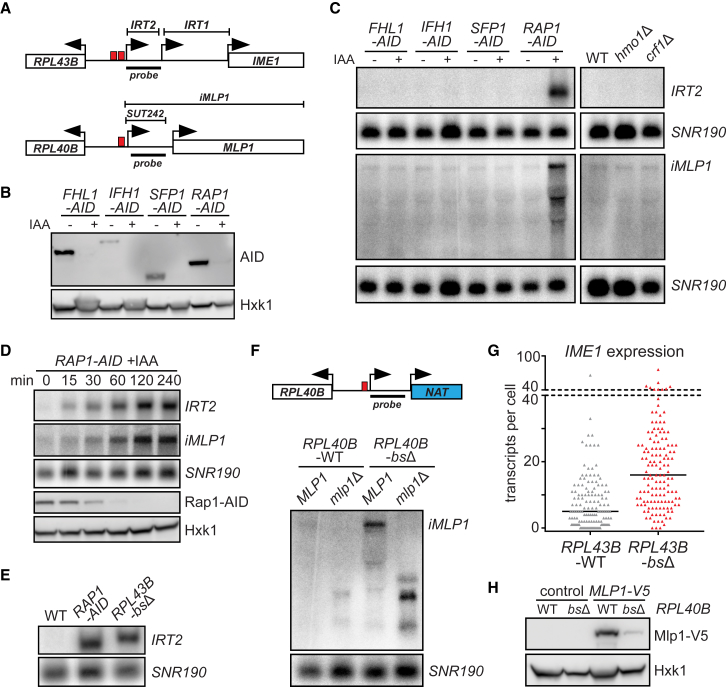
Rap1 Prevents Expression of Noncoding RNAs (A) Schematic of two divergent ribosomal protein (RP) gene promoters. Red boxes depict Rap1 motifs. (B) Auxin-induced depletion (AID) of transcription factors important for RP gene regulation detected by western blot. *FHL1-AID* (FW4200), *IFH1-AID* (FW4202), *SFP1-AID* (FW4204), and *RAP1-AID* (FW3877) cells were treated with 3-indole-acetic acid (IAA). Hxk1 was used as a loading control. (C) *IRT2* and *iMLP1* expression detected by northern blot in cells described in (B), WT (FW629), *hmo1*Δ (FW4132), and *crf1*Δ (FW4136). ^32^P-labeled probes targeting *IRT2* or *SUT242/iMLP1* and *SNR190* were used. (D) Similar to (C), *RAP1-AID* (FW3877) with multiple time points (+ IAA). (E) *IRT2* expression in *RPL43B-bsΔ* cells (FW3443). WT (FW629) and Rap1-depleted (FW3877) cells were included as in (C). (F) *iMLP1* expression in *RPL40B-bs*Δ (FW4141), *mlp1*Δ (FW6030), and *mlp1*Δ *RPL40B-bs*Δ (FW6029) cells. *NAT*, nourseothricin marker; replaced *MLP1*. (G) *IME1* expression in single diploid cells: WT (FW631) or *RPL43B-bs*Δ (FW6139). Each triangle represents transcript count for one cell and black lines indicate median number of transcripts per cell. n = 139 cells; ^∗^p < 0.0001 (unpaired Student’s t test). (H) Mlp1-V5 expression in WT (FW629), *RPL40B-bsΔ* (FW4141), *MLP1-V5* (FW4122), and *MLP1-V5 RPL40B*-*bs*Δ (FW4120) cells. See also Figure S1.

To investigate this, we deleted or depleted transcription factors important for RP gene regulation. We selected the *RPL43B* and *RPL40B* genes to study, since both promoters are directly adjacent to a divergent noncoding transcript: *IRT2* and *SUT242*, respectively (Figure 1A). Four RP gene transcription factors (Fhl1, Ifh1, Sfp1, and Rap1) have essential roles in cellular fitness (Hu and Li, 2007); hence, we generated auxin-inducible degron (AID) alleles (Nishimura et al., 2009) (Figure 1B). We measured the expression of divergent transcripts by northern blot using probes directed against *IRT2* and *SUT242*. No effects on *IRT2* and *SUT242* expression were observed when we depleted Fhl1, Ifh1, or Sfp1, or in *hmo1*Δ or *crf1*Δ cells (Figure 1C). Strikingly, Rap1-depleted cells (*RAP1-AID* + 3-indole-acetic acid [IAA]) showed strong induction of *IRT2* (Figure 1C). In addition, the *RPL40B* promoter displayed expression of multiple divergent transcripts upon Rap1 depletion. The transcript with the strongest signal approximated the size of the adjacent *MLP1* gene, which we define as isoform of *MLP1* (*iMLP1*). *IRT2* and *iMLP1* expression increased simultaneously as Rap1 protein levels decreased (Figures 1D and S1A). Thus, Rap1 specifically represses divergent transcription at the *RPL43B* and *RPL40B* promoters.

Rap1 is a pioneer transcription factor that binds to DNA sequence elements in RP and metabolic gene promoters (Lieb et al., 2001). To examine whether the Rap1 binding site (bs) is important for repressing divergent noncoding transcription, we deleted Rap1 motifs in the *RPL43B* and *RPL40B* promoters (*RPL43B-bs*Δ and *RPL40B-bs*Δ). *IRT2* and *iMLP1* expression levels increased in *RPL43B-bs*Δ and *RPL40B-bs*Δ, respectively, to a level comparable to Rap1-depleted cells (*RAP1-AID* + IAA) (Figures 1E, 1F, and S1B). Initiation of *IRT2* transcription occurred downstream of the Rap1 bs in *RPL43B-bs*Δ, because the *IRT2* transcript length increased due to the residual loxP sequence. Thus, Rap1 binding is required to repress divergent noncoding transcription from the *RPL43B* and *RPL40B* promoters.

Transcription within intergenic regions affects local coding gene expression (Ard et al., 2017). We examined the effect of divergent transcription on the expression of neighboring genes. Previous work showed that *IRT2* is part of a regulatory circuit that facilitates expression of *IME1*, the master regulator of entry into meiosis (Moretto et al., 2018). We hypothesized that Rap1 prevents mis-expression of *IRT2* from affecting *IME1* levels. Indeed, median *IME1* expression increased from 5 transcripts per cell in wild-type (WT) to 16 in the *RPL43B-bs*Δ mutant (Figures 1G and S1C). We also investigated the effect of *iMLP1* expression. *iMLP1* is a long transcript isoform of *MLP1*; when we deleted *MLP1* in the *RPL40B-bs*Δ cells, *iMLP1* disappeared and a shorter transcript appeared (Figure 1F). Mlp1 protein levels were markedly reduced in *RPL40B-bs*Δ, suggesting that *iMLP1* transcription affects expression of *MLP1* (Figures 1H and S1D). The 5′ extended sequence of *iMLP1* harbors 15 upstream AUG sequences, which may render *iMLP1* translationally inert similar to other 5′ extended transcript isoforms (Chen et al., 2017, Cheng et al., 2018, Chia et al., 2017). We conclude that mis-regulation of Rap1-repressed divergent transcripts affects neighboring gene expression.

### Rap1 Represses Noncoding Transcription near Its Binding Site

**Figure 2:**
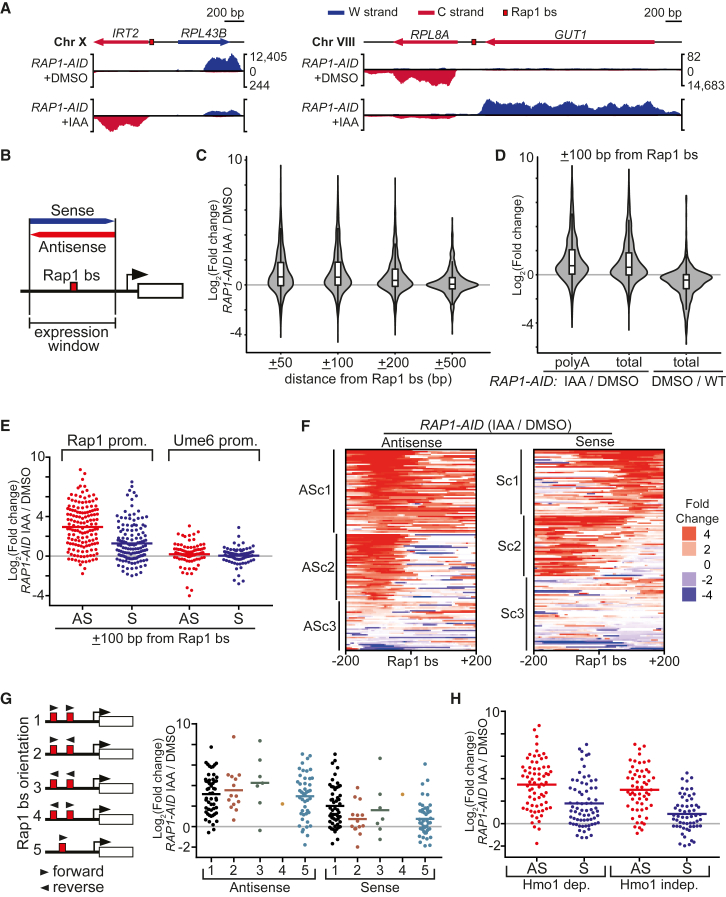
Rap1 Represses Divergent Noncoding Transcription (A) Examples of divergent noncoding RNAs repressed by Rap1. IAA- and DMSO-treated *RAP1-AID* (FW3877) cells were processed for total RNA-seq. Normalized reads (y axis) for the Watson (W, blue) and Crick (C, red) strands. (B) Scheme for determining RNA-seq signals around Rap1 binding sites. Reads that overlapped with the selected genomic region were counted. (C) Violin and box-and-whisker plots of total RNA-seq data as in (A), showing expression changes for different window sizes. n = 564 Rap1 binding sites, signals for W and C strands were computed separately. (D) Similar to C, comparing polyadenylated (poly(A)) and total RNA-seq data. As a control, the expression changes in *RAP1-AID* + DMSO over WT (FW629) are displayed (total RNA-seq). (E) Similar to (D), using scatterplots to display expression changes for antisense (AS) and sense (S) strand windows relative to the coding gene for Rap1 (n = 141) and Ume6 (n = 87) -regulated promoters. Horizontal red or blue lines: mean value. (F) Heatmaps showing changes in RNA expression on AS or S strands for data described in (E). Promoters were clustered on AS (ASc1–3) or S (Sc1–3) using k-means clustering (k = 3). (G) Different classes of RP promoters based on orientation of Rap1 motifs (red boxes) (left) and corresponding scatterplots of data described in (E) (right). (H) Similar to (E), but data are separated into Hmo1-dependent or -independent promoters. See also Figure S2.

We next investigated how depleting Rap1 affects noncoding transcription at a genome-wide scale by RNA sequencing (RNA-seq). We performed RNA-seq on both polyadenylated (poly(A)) and total RNA (Figure S2A). As expected, the expression of Rap1-regulated coding genes decreased upon Rap1 depletion (Figures S2B and S2C) (Knight et al., 2014, Lieb et al., 2001). In addition, *IRT2* and *iMLP1* expression increased in IAA-treated *RAP1-AID* cells, whereas the control (DMSO) did not show *IRT2* or *iMLP1* expression (Figures 2A and S2D). We also observed noncoding transcription from other RP gene promoters. For example, the *RPL8A* promoter expressed a divergent transcript spanning the neighboring *GUT1* gene, but antisense to the coding sequence (Figure 2A). Consequently, sense *GUT1* expression was reduced 1.7-fold. Thus, RNA-seq is able to identify novel Rap1-repressed divergent transcripts.

Our data from example loci indicate that Rap1 mediates repression of noncoding transcription close to the Rap1 binding sites. To systematically determine how Rap1 depletion affects noncoding transcription, we binned RNA-seq data in windows of 50, 100, 200, and 500 bp up- and downstream of 564 annotated Rap1 sites (Figure 2B) (Lieb et al., 2001, Rhee and Pugh, 2011). For smaller windows (50 and 100 bp), approximately 40% of Rap1 binding sites displayed increased RNA expression (>2-fold) upon Rap1 depletion (Figure 2C). For the larger windows (200 and 500 bp), the number of Rap1 sites showing increased RNA expression decreased to 30% and 16%, respectively, suggesting a spatial effect limited to regions harboring Rap1 binding sites. Our analyses with different window sizes showed little difference between RNA-seq data from poly(A) RNA and total RNA (Figures 2D and S2E). In conclusion, Rap1 represses transcription near Rap1 binding sites across the genome.

Next, we analyzed the RNA-seq data to identify features of cryptic transcript repression by Rap1. First, we determined whether there is a bias for the orientation of Rap1-repressed transcripts. We selected 141 Rap1 binding sites at well-annotated gene promoters regulated by Rap1 for further analysis (mostly RP genes) (Knight et al., 2014, Lieb et al., 2001). Expression near the Rap1 binding sites was upregulated in both the sense and antisense direction after Rap1 depletion; however, the largest increase was detected in the antisense direction (Figure 2E). A control set of promoters regulated by the repressor Ume6 was not affected by Rap1 depletion (McKnight et al., 2016). Second, we clustered the data centered on the Rap1 binding sites (Figures 2F and S2F–S2H). The antisense clusters 1 and 2 (ASc1 and ASc2) both displayed increased expression upstream of the Rap1 binding sites, while ASc3 showed very mild increase (Figure 2F). When we clustered for the sense direction signals, we observed that transcripts were upregulated both up- and downstream of the Rap1 site (Sc1 and Sc2). Finally, we examined whether the transcripts induced upon Rap1 depletion were enriched for specific classes of RP gene promoters (Knight et al., 2014). We found that the orientation or number of Rap1 motifs had little effect (Figure 2G). In addition, promoters regulated by Hmo1 displayed a comparable increase in expression to Hmo1-independent promoters (Figure 2H). Thus, Rap1 represses transcription near the Rap1 binding sites in the antisense, and to lesser extent, the sense direction.

### A Proximal Rap1 Motif Is Required and Sufficient to Repress Divergent Transcription

**Figure 3:**
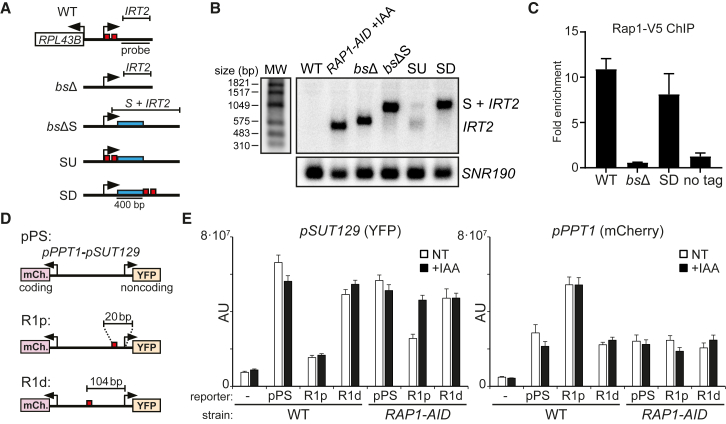
A Proximal Rap1 Motif Is Required and Sufficient to Repress Divergent Transcription (A) Schematic of mutants (*bs*Δ, FW3440; *bs*ΔS, FW3920; SU, FW7451; SD, FW3922). Blue bar, spacer sequence; red boxes, Rap1 binding sites. (B) *IRT2* expression in mutants described in A, WT (FW629), and *RAP1-AID* + IAA (FW3877). Northern blot membranes were probed for *IRT2* and *SNR190*. MW, RNA molecular weight marker. (C) Rap1 binding to *RPL43B* promoter measured by chromatin immunoprecipitation (ChIP) (FW4732, FW4734, FW4735, and FW629). Data were normalized over *ACT1* and plotted as mean ± SEM (n = 3). (D) Schematic of fluorescent reporter constructs. pPS was described previously (Marquardt et al., 2014). The Rap1 sites from the *RPL43B* promoter were integrated at a proximal (R1p, 20 bp) or distal (R1d, 104 bp) position to the TSS of *SUT129*. (E) Ectopic repression of divergent noncoding transcription by Rap1. WT (FW629), pPS, R1p, and R1d in WT or *RAP1-AID* (FW6407; FW6895; FW7253; FW6208; FW6206; FW6408) cells were not treated (NT) or treated (+IAA), fixed, and imaged. Mean signals corrected for background (AU, arbitrary units) were plotted + 95% confidence intervals (n = 50 cells per sample). See also Figure S3.

Our results demonstrate that Rap1 represses noncoding transcription near the Rap1 binding sites. If close proximity of the Rap1 binding site to the cryptic promoter sequence is important, then increasing the distance should impair repression of noncoding transcription. To test this, we integrated 400-bp spacer sequences upstream or downstream of the Rap1 motifs relative to the *RPL43B* promoter (Figures 3A and 3B). When we integrated a spacer to replace Rap1 binding sites (*bs*ΔS), *IRT2* was expressed and the size of the transcript approximated the spacer sequence plus *IRT2* (S + *IRT2*) indicating that initiation of *IRT2* occurs downstream of the Rap1 binding sites. Strikingly, we observed a similar pattern when we integrated the spacer directly downstream (SD), but not upstream (SU), of the Rap1 binding sites relative to *RPL43B*. The spacer sequence had no effect on Rap1 binding to the *RPL43B* promoter (Figure 3C). Thus, the Rap1 binding sites must be nearby the cryptic promoter sequence for repression of divergent transcription.

Next, we determined whether the Rap1 binding site is sufficient to repress divergent transcription. We integrated Rap1 motifs in a fluorescent reporter construct that harbors a divergent promoter transcribing *PPT1* in the coding direction and *SUT129* in the noncoding direction (pPS) (Figures 3D and S3A) (Marquardt et al., 2014). A Rap1 motif proximal to the *SUT129* promoter (R1p) lowered yellow fluorescent protein (YFP) levels, while *PPT1* (mCherry) activity increased (Figure 3E). *SUT129* promoter (R1p) activity increased to match control plasmid (pPS) levels upon Rap1 depletion (*RAP1-AID* + IAA). The repression of *SUT129* by Rap1 was not dependent on transcription regulation in the coding direction because in *RAP1-AID* (IAA or NT) cells the *PPT1* signal matched WT (Figure 3E, right panel). The results were comparable using a reporter with Rap1 motifs in the reverse orientation (R1prv) (Figure S3B). Finally, a more distal Rap1 binding site (R1d) to *SUT129* showed comparable YFP levels to the WT reporter, and Rap1 depletion had little effect (Figure 3E). Thus, the Rap1 motif is sufficient to repress divergent noncoding transcription when located near the cryptic promoter sequences.

### TSS Mapping of Rap1-Repressed Divergent Noncoding Transcripts

**Figure 4:**
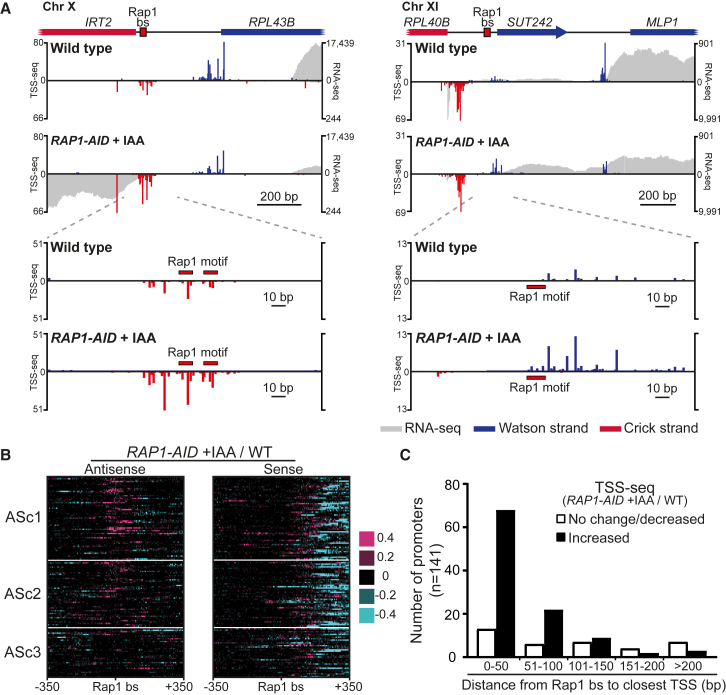
Rap1 Represses Divergent Transcription Close to Its Binding Site (A) Transcription start sites (TSSs) at *RPL43B* and *RPL40B* in WT (FW629) and *RAP1-AID* + IAA (FW3877) cells. TSS-seq signal per million reads and total RNA-seq data are plotted for both strands. (B) TSS-seq difference heatmap near 141 promoter Rap1 binding sites ordered as in Figure 2F (ASc1–3). Pink regions depict higher TSS-seq signal (5 bp bin size) in *RAP1-AID* + IAA versus WT; cyan regions depict lower TSS-seq signal. (C) Distribution of TSSs near promoter Rap1 binding sites from data described in (A) and (B). The distance from the Rap1 binding sites (n = 141) to the closest TSS was measured and their frequency is plotted in bins of 50 bp. See also Figure S4.

To investigate the relationship between Rap1 motifs and cryptic promoters at a genome-wide scale, we mapped transcription start sites by sequencing (TSS-seq) in WT and Rap1-depleted cells (Figures S4A–S4C). At the *RPL43B* promoter, a cluster of multiple TSSs was detected in a region of 35 bp up- and downstream of the Rap1 motifs in Rap1-depleted cells, and to a lesser extent, in WT cells (Figure 4A, left panel). The signals are unlikely to originate from abortive RNA polymerase II initiation because the TSS-seq procedure isolates poly(A) and capped RNAs. At the *RPL40B* promoter, multiple *iMLP1* TSSs in a region of 23 bp were detected directly upstream of the Rap1 binding site in the *RPL40B* promoter, and the TSS-seq signals increased upon Rap1 depletion (Figure 4A, right panel). Conversely, the *MLP1* protein coding TSS signal decreased in Rap1-depleted cells supporting our observation that Mlp1 protein levels decreased in cells mis-expressing *iMLP1*.

Next, we computed the changes in TSS signals between WT and Rap1-depleted cells. The TSS-seq data matched the RNA-seq data well over a wide range of expression (Figure S4D). ASc1 and ASc2 clusters displayed increased TSS signals around the Rap1 binding sites, whereas there were fewer differences in cluster 3 (ASc3) (Figures 2F and 4B, antisense). As expected, TSS signals decreased in the sense direction downstream of the Rap1 binding sites in Rap1-depleted cells because coding gene expression was reduced (Figure 4B, sense). Interestingly, sequences directly upstream of the canonical coding transcript TSSs displayed increased TSS signals in the sense direction, suggesting that Rap1 is also important for TSS selection (Challal et al., 2018). Finally, most Rap1-regulated promoters examined contained an antisense TSS as the nearest one to the Rap1 binding site (82% antisense, 18% sense). Approximately 50% of the promoters displayed (>2-fold) increased TSS signals within 50 bp of the Rap1 motif in Rap1-depleted cells (Figures 4C and S4E). Thus, Rap1 represses initiation of divergent transcription close to its promoter regulatory elements.

### The Rap1 C-Terminal Domain Contributes to Repressing Divergent Transcription

**Figure 5:**
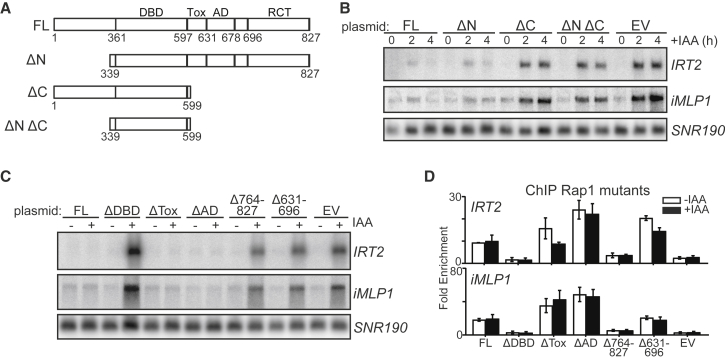
The Rap1 C-Terminal Domain Contributes to Repression of Divergent Transcription (A) Schematic of Rap1 domains and truncation mutants. DBD, DNA-binding domain; Tox, toxicity domain; AD, activation domain; RCT, Rap1 C-terminal interacting domain. (B) *IRT2* and *iMLP1* expression in Rap1 mutants detected by northern blot. *RAP1-AID* (FW3877) cells expressing full-length Rap1 (FL) (FW5129), ΔN (FW5133), ΔC (FW5138), ΔN ΔC (FW5141), or an EV (FW5145) were used. (C) *IRT2* and *iMLP1* expression in different Rap1 domain mutants (FL [FW4948]; ΔDBD [FW4950]; ΔTox [FW4952]; ΔAD [FW4954]; Δ764–827 [FW4958]; Δ631–696 [FW4960]; EV [FW5145]). (D) Rap1 binding at *RPL43B* (*IRT2*) and *RPL40B* (*iMLP1*) promoters for mutants in (C) (FW5420, FW5393, FW5394, FW5424, FW5395, FW5396, and FW5399), measured by ChIP. Data normalized over *ACT1* and plotted as mean ± SEM (n = 3) are shown. See also Figure S5.

Functional domains of Rap1 are important in gene repression and activation (Azad and Tomar, 2016), so we examined whether repression of divergent transcription requires a specific domain. We generated deletions in the N- and C-terminal domains of Rap1 (Figure 5A). The Rap1 fragments were expressed in *RAP1-AID* cells (Figure S5A). As expected, full-length Rap1 (FL, 1–827) maintained repression of *IRT2* and *iMLP1* expression upon Rap1 depletion (*RAP1-AID* + IAA), whereas the empty vector (EV) control displayed divergent transcription (Figure 5B). A deletion of the N terminus (ΔN, 339–827) rescued Rap1 depletion. Cells harboring deletions in the C terminus (ΔC, 1–599) or N and C termini (ΔN ΔC, 339–599) displayed expression of *IRT2* and *iMLP1*. The Rap1 DNA binding domain represses divergent transcription to some extent because *IRT2* and *iMLP1* expression decreased in ΔN ΔC to ∼70% of the EV (Figure S5B).

Important functions of Rap1 are exerted by the C-terminal silencing domain, the activation domain (AD), and the toxicity domain (Tox) (Freeman et al., 1995, Garbett et al., 2007, Layer et al., 2010, Sussel and Shore, 1991). We assessed the ability of Rap1 domain deletion mutants to repress divergent transcription (Figures 5A and S5C–S5E) (Layer et al., 2010). We found that Rap1ΔTox and Rap1ΔAD did not affect *IRT2* and *iMLP1* repression, whereas mutants lacking the DNA binding domain (Rap1ΔDBD), the silencing domain (Rap1Δ764–827), or the AD plus an adjacent sequence (Rap1Δ631–696) failed to repress *IRT2* and *iMLP1* (Figure 5C). Except for Rap1ΔDBD and Rap1Δ764–827, the Rap1 C-terminal mutants associated at the *RPL43B* (*IRT2*) and *RPL40B* (*iMLP1*) promoters (Figures 5D and S5D). We also examined whether different point and patch mutations in the Rap1 silencing domain, already characterized for telomere regulation and hidden mating-type loci silencing, affected repression of *IRT2* expression (Feeser and Wolberger, 2008). We found that none of the mutants caused a significant increase in *IRT2* expression indicating the Rap1 silencing domain is not important for repressing divergent transcription (Table S1). In conclusion, part of Rap1 C terminus, which includes the AD but not the silencing domain, contributes to repression of divergent transcription.

### RSC Chromatin Modeler Elicits Divergent Transcription in the Absence of Rap1

**Figure 6:**
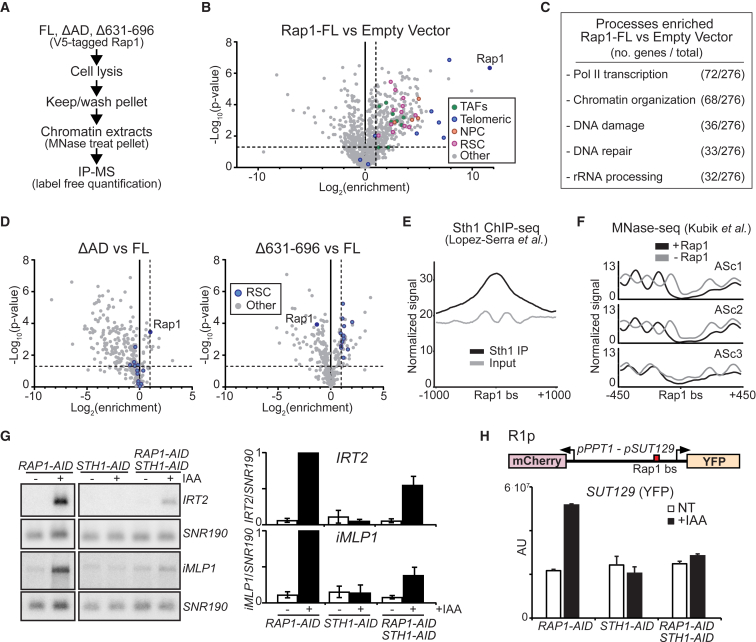
RSC Chromatin Modeler Elicits Divergent Transcription in the Absence of Rap1 (A) Scheme to identify proteins interacting with chromatin bound Rap1. FL (FW5420), ΔAD (FW5424), Δ631–696 (FW5396), and EV (FW5399) were affinity purified and processed for LC-MS label-free quantification (LFQ). (B) Volcano plot showing differences in protein enrichment for Rap1-V5 (FL versus EV). Enrichment (log_2_) versus p value (unpaired two-sample t test, –log_10_ scale) for n = 916 identified proteins plotted. Horizontal dashed line: 1.303 (p = 0.05); vertical dashed line: 2-fold enrichment. (C) Yeast GO-Slim Process analysis of data described in (B). (D) Volcano plots of ΔAD versus FL (left) and Δ631–696 versus FL (right). Proteins that were enriched in FL versus EV (n = 289 proteins) as described in (B) are plotted. (E) Metagene plots of Sth1 ChIP-seq for Rap1-regulated promoters (n = 141), centered on Rap1 binding sites (GEO: GSE56994) (Lopez-Serra et al., 2014). (F) Metagene plots of MNase-seq data (GEO: GSE73337) (Kubik et al., 2015) for clusters described in Figure 2F (ASc1–3). The signals in the presence (black) or absence (gray) of Rap1 are displayed. (G) *IRT2* and *iMLP1* expression in cells depleted for Rap1, Sth1, or both (FW3877; FW6032; FW6231). Membranes were probed for *IRT2*, *iMLP1*, and *SNR190* (left). Quantification of *IRT2* and *iMLP1* expression (right). Mean values ± SEM are plotted (n = 3). (H) *SUT129* promoter activity upon co-depletion of RSC and Rap1 (FW6206; FW6218; FW6433). Cells were grown, treated, processed, and imaged as described in Figure 3E. Mean signals corrected for background (AU, arbitrary units) were plotted + 95% confidence intervals (n = 50 cells per sample). See also Figure S6.

Given that the Rap1Δ631–696 mutant displayed divergent transcription but maintained its ability to bind the Rap1 motif, we hypothesized this mutant may associate differently with corepressors or activators of divergent transcription. To identify candidate regulators, we affinity-purified Rap1 from micrococcal nuclease (MNase) solubilized chromatin and used proteomics mass spectrometry to identify associated proteins (Figure 6A) (van Werven et al., 2008). We compared full-length Rap1 (Rap1-FL), Rap1ΔAD, Rap1Δ631–696, and an empty vector (EV) control (Figure S6A; Table S2). Several proteins known to interact with Rap1 were enriched in Rap1-FL versus EV: TAFs, telomere-related proteins, and nuclear pore complex (NPC) proteins (Layer et al., 2010, Van de Vosse et al., 2013) (Figure 6B). In addition, we identified multiple subunits of the RSC complex (12 out of 17). As expected, enriched proteins were involved in RNA polymerase II transcription and chromatin organization (Figures 6C and S6B). Next, we searched for interacting proteins that showed differential enrichment between Rap1Δ631–696 and Rap1-FL but were not altered in Rap1ΔAD. We found that all identified subunits of RSC were enriched in Rap1Δ631–696 suggesting that Rap1 negatively affects RSC association to the local chromatin environment (Figure 6D).

RSC (remodels the structure of chromatin) is an ATP-dependent chromatin remodeling complex, and it generates NDRs at promoters to facilitate gene activation (Cairns et al., 1996, Clapier et al., 2017). The ATPase subunit of RSC, Sth1, binds near promoter Rap1 binding sites, supporting our observation that RSC interacts with chromatin bound Rap1 (Figures 6E and S6C) (Lopez-Serra et al., 2014, Parnell et al., 2015). RSC interacts with nucleosomes and DNA directly and does not require Rap1 for promoter association or action (Krietenstein et al., 2016, Kubik et al., 2015, Kubik et al., 2018). In line with this observation, a narrow NDR is maintained at promoters in the absence of Rap1 (Figures 6F and S6D). It is worth noting that for the clusters with high levels of divergent transcription (ASc1 and ASc2) nucleosomes are highly organized directly upstream of the Rap1 motif, likely due to transcription-coupled chromatin remodeling (Figures 6F and S6D) (Venkatesh and Workman, 2015).

We hypothesized that RSC promotes divergent transcription in the absence of Rap1. To test this, we depleted Sth1 together with Rap1 (Figure S6E). Depleting Sth1 (*STH1-AID* + IAA) had no effect on *IRT2* and *iMLP1* expression (Figure 6G). When Rap1 and Sth1 were co-depleted, *IRT2* and *iMLP1* expression was greatly reduced compared to Rap1 depletion alone (Figures 6G and S6F). Depleting RSC also suppressed divergent transcription when we used the *PPT1*/*SUT129* reporter plasmid harboring proximal Rap1 sites (R1p, Figures 6H and S6G). Taken together, we propose that Rap1 reduces RSC association to the local chromatin environment and is positioned to repress divergent noncoding transcription—restricting RSC to stimulate productive coding transcription instead.

### Chromatin Regulators Control Divergent Transcription in a Manner Distinct from Rap1

Chromatin remodelers and histone modifying enzymes play essential roles in repressing noncoding transcription (Venkatesh and Workman, 2015). Thus, chromatin regulators may mediate Rap1-dependent repression of divergent transcription. To identify repressors of divergent transcription, we measured *IRT2* and *iMLP1* expression levels in 62 gene deletion and depletion strains (Table S3). Specifically, we selected genes that are (1) involved in cryptic or divergent transcription (e.g., Set2, Set3, and Spt16), (2) known to interact with Rap1 (e.g., Sir3, Rif1, and Rif2), or (3) regulate chromatin and transcription.

**Figure 7:**
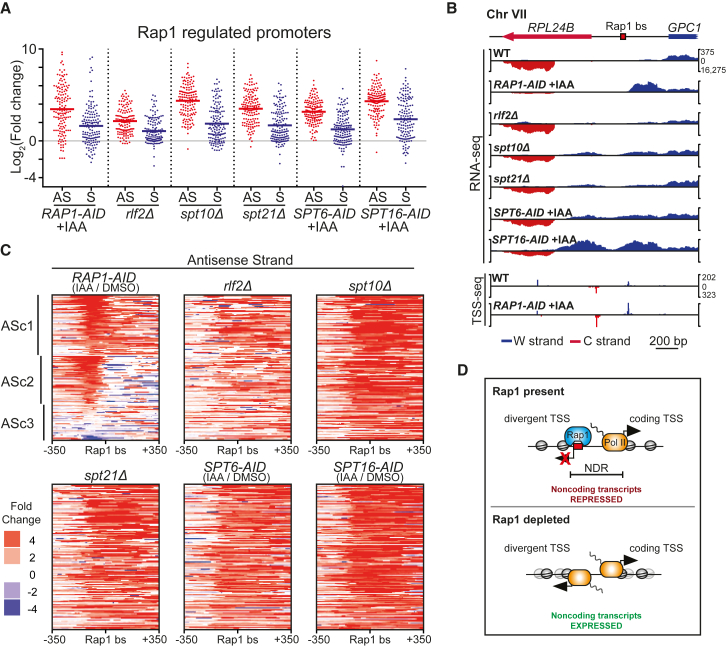
Chromatin Regulators Control Divergent Transcription in a Manner Distinct from Rap1 (A) Scatterplots showing the changes in RNA expression around Rap1 binding sites for *spt10Δ* (FW5543), *spt21Δ* (FW5547), *rlf2Δ* (FW5609) compared to WT (FW629), and for *RAP1-AID* (FW3877), *SPT6-AID* (FW5555), and *SPT16-AID* (FW5559) cells (IAA/DMSO). Changes in RNA expression were calculated for n = 141 promoter Rap1 sites ±100 bp for AS and S strands. Horizontal red or blue bars indicate mean values. (B) Data from (A) of the *RPL24B* locus including TSS-seq data described in Figure 4. (C) Heatmaps displaying the data from (A), clustered as in Figure 2F. (D) Model for Rap1-mediated repression of divergent transcription. Rap1 promotes transcription from the coding direction TSS (black arrows) but represses initiation from the divergent TSS. When Rap1 is absent, transcription in the divergent direction occurs. See also Figure S7.

Fourteen mutants displayed increased *iMLP1* expression. Only depletion of Spt16 (*SPT16-AID +*IAA) increased *IRT2* expression. When we compared *iMLP1* expression patterns from our data to a published dataset, we found that five mutants overlapped, which we decided to study further (van Bakel et al., 2013). These were (1) putative histone acetyltransferase Spt10, (2) transcription factor Spt21, (3) CAF-1 chromatin assembly complex component Rlf2, (4) chromatin remodeler and elongation factor Spt6, and (5) FACT (facilitates chromatin transcription) complex component Spt16. All candidates have known roles in the repression of divergent or cryptic transcription (Cheung et al., 2008, DeGennaro et al., 2013, Marquardt et al., 2014, Mason and Struhl, 2003). We performed RNA-seq with gene deletion or depletion mutants and observed increased expression within Rap1-regulated promoters (Figures 7A and S7A–S7C).

Next, we examined whether these five chromatin regulators mediate repression of divergent transcription by Rap1. We found little overlap between Rap1-repressed divergent transcripts and transcripts repressed by the chromatin regulators. To illustrate, the *RPL24B* and *RPL40B* promoters showed antisense transcription downstream of the Rap1 motif nearer to or within the coding gene in *rlf2*Δ, *spt10*Δ, and *spt21*Δ cells, and in cells depleted for Spt6 or Spt16 (Figures 7B and S7D). We also identified promoters (*RPL25* and *RPL43B*) that displayed no detectable divergent transcription in the five mutants, while there was a clear signal in Rap1-depleted cells (Figure S7D). The five depletion or deletion mutants displayed increased antisense transcription initiating downstream of the Rap1 binding sites, but not near Rap1 binding sites (Figures 7C and S7E). Taken together, Rap1 acts in concert with chromatin regulators to repress divergent transcription, but in a distinct manner that is spatially limited.

## Discussion

Here, we describe how highly expressed coding gene promoters limit divergent noncoding transcription in yeast. We identify a surprising role for the pioneer transcription factor Rap1. We find that Rap1 represses divergent noncoding transcription at its binding motif and adjacent sequences. Our data demonstrate how a sequence-specific transcription factor can prevent regulatory sequences from producing aberrant transcripts and define a mechanism for providing directionality toward productive transcription.

### Mechanism for Rap1-Mediated Repression of Divergent Transcription

Several lines of evidence indicate that Rap1 represses divergent noncoding transcription, uncoupled from transcription regulation in the coding direction. First, Rap1 represses divergent transcription near the Rap1 binding site. Second, abrogating other transcription factors important for RP gene expression did not affect divergent transcription. Third, close proximity of the Rap1 binding site to the cryptic promoter is essential for repressing divergent transcription. Fourth, the Rap1 binding site ectopically represses divergent noncoding transcription without affecting transcription in the protein-coding direction. Conversely, the AD of Rap1, which directs transcription in the protein-coding direction, is not required for repressing divergent transcription. Finally, we provide evidence that chromatin regulators repress divergent transcription by mechanisms distinct from Rap1.

Promoter directionality is shaped by evolution toward protein-coding genes through enrichment of DNA binding protein motifs (Jin et al., 2017). In this context, Rap1 promotes directionality in multiple ways. First, Rap1 recruits cofactors and basal transcription machinery, which promote transcription in the coding direction (Azad and Tomar, 2016, Hu and Li, 2007). Second, Rap1 asymmetrically occupies the promoter NDR at the 5′ end, where it represses the divergent core promoter (Figure 7C). Core promoters are intrinsically directional (Duttke et al., 2015), and two independent pre-initiation complexes initiate divergent transcription at mRNA-noncoding RNA pairs in yeast (Rhee and Pugh, 2012). Hence, repressing transcription initiation at the antisense core promoter regulates promoter directionality. As a consequence, upstream regulatory elements or transcription factor binding sites should not overlap with core promoters to avoid concurrent steric interference. In mammalian cells, some pioneer transcription factors also open chromatin asymmetrically (Sherwood et al., 2014)—suggesting that repression of divergent transcription by transcription factors could be broadly conserved.

We found no evidence that Rap1 silencing and roadblock functions, and cofactors of Rap1, are important for repression of divergent transcription (Candelli et al., 2018, Yarrington et al., 2012). Typically, the Rap1 roadblock acts as a failsafe mechanism by terminating upstream transcriptional readthrough toward the downstream coding gene. In contrast, most Rap1-repressed divergent transcripts initiate antisense and upstream of the Rap1 motif, so there is no potential roadblock downstream of the divergent TSS. We find no evidence for contributions from other Rap1 cofactors suggesting that Rap1 itself represses divergent transcription directly. In this context, Rap1-mediated repression of divergent transcription shows parallels to prokaryotic operon regulation and synthetic transcriptional repression systems. In bacteria, transcriptional repressors bind operon sequences near TSSs and directly prevent recruitment of RNA polymerase through steric hindrance (Browning and Busby, 2004). Similarly, Rap1-mediated repression of divergent core promoters could also act through steric hindrance. Like bacterial repressors, Rap1 binds near the TSS of (divergent) core promoters. In eukaryotes, direct steric repression of transcription can be achieved by dCas9 CRISPR interference and transcription activator-like effector repressors (TALERs) when targeted near TSSs (Gilbert et al., 2013, Li et al., 2015).

### A Model for Control of Divergent Noncoding Transcription by Rap1

Interestingly, the C terminus of Rap1 contributes to repression of divergent transcription. Perhaps the region we identified (residue 631–696) contributes to exclusion or activity of other factors. The Rap1 C terminus may modulate the affinity and binding mode of the DNA binding domain (Feldmann et al., 2015). Consistent with our observations, a study by Challal et al. also found that Rap1 regulates the fidelity of TSS selection at gene promoters (Challal et al., 2018). It is worth noting that this study showed that the DNA binding domain of Rap1 is, at least in part, sufficient to prevent initiation at aberrant TSSs. Future work should clarify the contributions of the Rap1 DNA-binding and C-terminal domains toward repression of noncoding transcription.

We find that Rap1 controls the activity or action of RSC, because in Rap1-depleted cells RSC elicits divergent transcription. Our data indicate that Rap1 restricts RSC to stimulate productive transcription in the protein-coding direction. Recently, it was shown that RSC helps maintain an NDR in the absence of Rap1 (Kubik et al., 2018). A RSC-dependent NDR could contribute to divergent transcription in the absence of Rap1. We propose that, in WT cells, Rap1 competes locally with the binding of activators of divergent transcription, such as RSC and basal transcription machinery (Figure 7D).

Decades of work have shown that eukaryotes have adopted redundant strategies to limit expression of aberrant noncoding RNAs. Mis-regulation of divergent noncoding transcripts could have negative effects on local or global gene expression, especially in gene-dense genomes such as that in budding yeast. Repression of cryptic transcription nearby regulatory elements, as we have shown for Rap1, could be a conserved property of sequence-specific transcription factors and other DNA binding proteins.

## STAR★Methods

### Key Resources Table

**Table:** 

REAGENT or RESOURCE	SOURCE	IDENTIFIER
**Antibodies**		

Anti-V5 tag (mouse) antibody	Thermo Fisher Scientific	R96025; RRID: AB_2556564
Anti-hexokinase (rabbit) antibody	US Biological	H2035; RRID: AB_2629457
Anti-Myc tag (mouse) antibody	Merck Millipore	05-724; RRID: AB_309938
Anti-HA tag (mouse) antibody	This paper	12CA5
Anti-FLAG tag (mouse) antibody	Sigma-Aldrich (Merck)	F3165; RRID: AB_259529
Anti-mouse IgG HRP-linked antibody	GE Life Sciences	NA931V; RRID: AB_772210
Anti-rabbit IgG HRP-linked antibody	GE Life Sciences	NA934V; RRID: AB_772206
Anti-V5 agarose affinity gel	Sigma-Aldrich (Merck)	A7345; RRID: AB_10062721

**Chemicals, Peptides, and Recombinant Proteins**		

Indole-3-acetic acid (IAA, auxin)	Sigma-Aldrich (Merck)	I3750
ULTRAhyb Ultrasensitive Hybridization Buffer	Thermo Fisher Scientific	AM8670
Prime-It II Random Primer Labeling Kit	Agilent	300385
dATP [α-32P]	PerkinElmer	NEG512H500UC
dATP [α-32P]	Hartmann Analytic	SRP-203
Proteinase K	Thermo Fisher Scientific	EO0491
rDNase	Machery-Nagel	740963
Micrococcal Nuclease (MNase)	NEB	M02475
RNA Fragmentation Reagents (Ambion)	Thermo Fisher Scientific	AM8740
Shrimp Alkaline Phosphatase (rSAP)	NEB	M0371L
Cap-Clip Acid Pyrophosphatase	CellScript	C-CC15011H
T4 RNA ligase 1 (high concentration)	NEB	M0437M
SuperScript IV Reverse Transcriptase	Thermo Fisher Scientific	18090050
RNasin Plus Ribonuclease Inhibitor	Promega	N2115
RNase H	NEB	M0297L
RNase cocktail	Thermo Fisher Scientific	AM2286
Dynabeads MyOne Streptavidin C1	Thermo Fisher Scientific	65002
NotI-HF	NEB	R3189M
T3 RNA polymerase	NEB	M0378S
Vaccinia capping enzyme	NEB	M2080S
TURBO DNase	Thermo Fisher Scientific	AM2238
Linear acrylamide	Thermo Fisher Scientific	AM9520
*IME1* and *ACT1* single molecule RNA-FISH probes	Biosearch Technologies	N/A

**Critical Commercial Assays**		

PowerUp SYBR Green Master Mix	Thermo Fisher Scientific	A25742
Amersham ECL Prime Western Blotting Detection Reagent	GE Life Sciences	RPN2232
Ribo-Zero Gold rRNA Removal Kit (Yeast)	Illumina	MRZY1324
TruSeq Stranded Total RNA	Illumina	RS-122-2202
TruSeq Stranded mRNA	Illumina	RS-122-2101
Poly(A)Purist MAG Kit	Thermo Fisher Scientific	AM1922
2100 Bioanalyzer	Agilent	G2939BA
HighPrep PCR	MagBio	AC-60050
Qubit dsDNA HS Assay Kit	Thermo Fisher Scientific	Q32851
KAPA HiFi HotStart ReadyMix PCR Kit	KAPA Biosystems (Roche)	KK2602
KAPA Hyper Prep Kit	KAPA Biosystems (Roche)	KK8504
KAPA SI Adaptor Kit Set A+B (30 uM)	KAPA Biosystems (Roche)	KK8700
RNeasy MinElute Cleanup Kit	QIAGEN	74204

**Deposited Data**		

Total, poly(A), and TSS RNA sequencing	This paper	GEO: GSE110004
Sth1 ChIP-seq	Lopez-Serra et al., 2014	GEO: GSE56994
Sth1 MNase ChIP-seq	Parnell et al., 2015	GEO: GSE65594
MNase-seq	Kubik et al., 2015	GEO: GSE73337

**Experimental Models: Organisms/Strains**		

*S. cerevisiae:* Strain background: BY, see Table S4	This paper	N/A

**Oligonucleotides**		

Oligonucleotides and primers, see Table S6	This paper	N/A

**Recombinant DNA**		

Plasmids, see Table S5	This paper	N/A

**Software and Algorithms**		

Cutadapt (version 1.9.1)	Martin, 2011	https://cutadapt.readthedocs.io/en/stable/
RSEM (version 1.3.0)	Li and Dewey, 2011	https://github.com/deweylab/RSEM
STAR (version 2.5.2a)	Dobin et al., 2013	https://github.com/alexdobin/STAR
DESeq2 (version 1.12.3)	Love et al., 2014	https://doi.org/10.18129/B9.bioc.DESeq2
BWA (version 0.5.9-r16)	Li and Durbin, 2009	http://bio-bwa.sourceforge.net/
DANPOS2 (version 2.2.2)	Chen et al., 2013	https://sites.google.com/site/danposdoc/
Subread (version 1.5.1)	Liao et al., 2014	http://subread.sourceforge.net/
SAMTools (version 1.3.1)	Li et al., 2009	http://www.htslib.org/
BEDTools (version 2.26.0)	Quinlan and Hall, 2010	https://bedtools.readthedocs.io/
BigWig and BigBed	Kent et al., 2010	http://hgdownload.soe.ucsc.edu/admin/exe/linux.x86_64/
MaxQuant (version 1.6.01)	Cox and Mann, 2008	http://www.coxdocs.org/doku.php?id=:maxquant:start
Perseus (version 1.4.0.2)	Tyanova et al., 2016	http://www.coxdocs.org/doku.php?id=:perseus:start
ImageJ (version 1.48k)	Schneider et al., 2012	https://imagej.nih.gov/ij/index.html
SGD Gene Ontology Slim Mapper	Saccharomyces Genome Database	https://www.yeastgenome.org/cgi-bin/GO/goSlimMapper.pl

### Contact for Reagent and Resource Sharing

Further information and requests for resources and reagents should be directed to the Lead Contact, Folkert van Werven (folkert.vanwerven@crick.ac.uk).

### Experimental Model and Subject Details

#### Yeast Strains

Strains isogenic to the *Saccharomyces cerevisiae* BY strain background were used throughout this study. The genotypes are listed in Table S4. Gene deletions were generated using the one-step disruption protocol as described previously (Longtine et al., 1998). The gene deletion strains used to examine mis-regulation of *IRT2* and *iMLP1* expression in Table S4 were described previously (Winzeler et al., 1999).

#### Growth and Conditions

Cells were grown in YPD media (1% w/v yeast extract, 2% w/v peptone, 2% w/v glucose, supplemented with tryptophan (96 mg/L), uracil (24 mg/L) and adenine (12 mg/L). Cells were cultured with shaking in conical flasks at 30°C. For single molecule RNA FISH experiments described in Figure 1, diploid cells were grown to saturation in nutrient-rich YPD media, then shifted to sporulation media (SPO, 0.3% w/v potassium acetate and 0.02% w/v raffinose) to OD_600_ 1.8 and immediately fixed with formaldehyde (3% v/v). Cells were collected immediately after resuspending in SPO media.

For auxin induced depletion experiments, 3-indole-acetic acid (IAA) (Sigma-Aldrich) was used to induce AID-tagged protein depletion. 1 M IAA stocks were prepared in dimethyl sulfoxide (DMSO) and added directly to cultures to a final concentration of 500 μM.

### Method Details

#### Plasmids and Yeast Transformation

A one-step tagging procedure was used for generating C-terminal auxin-inducible degron (AID) alleles (*RAP1-AID, FHL1-AID, IFH1-AID, SFP1-AID, SPT6-AID, SPT16-AID, and STH1-AID*), which contains three copies of the V5 epitope and the IAA7 degron (Nishimura et al., 2009). The *RAP1-AID-MYC* allele harbors nine copies of the Myc epitope and *IAA17* residues 71-114 (AID-MYC), and *STH1-AID-FLAG* allele contains six copies of the FLAG epitope and *IAA17* residues 71-114 (AID-FLAG) (Morawska and Ulrich, 2013). The AID strains also contained a single copy integration plasmid expressing *Oryza sativa* TIR1 (*osTIR1*) ubiquitin E3 ligase from the *GPD1* promoter (gift from Leon Chan). *osTIR1* plasmids were linearized by digestion with PmeI and integrated at either the *HIS3* or *LEU2* locus.

All Rap1 mutants and truncation constructs were expressed from single copy integration plasmids (gift from Wendell Lim) in *RAP1-AID* or *RAP1-AID-MYC* strain backgrounds. The truncation or domain deletion mutants described in Figure 5 were cloned from *RAP1* plasmids (gift from Amanda Johnson and Tony Weil) by NotI and XhoI digestion into plasmid 372 to generate plasmids 471-474, and by SacI and KpnI digestion to generate plasmids 477-483 (Garbett et al., 2007, Layer et al., 2010). Three copies of the V5 epitope tag from strain 4732 were introduced by Gibson-style cloning (NEBuilder HiFi, NEB) at the C terminus of Rap1 in plasmids 477-483 to generate plasmids 558, 559, 561, 562, 566, and 568 used for ChIP in Figure 5D.

Single copy integration *RAP1* expression plasmids containing C-terminal point and patch mutations were re-cloned from plasmids described previously (gift from Cynthia Wolberger) by Gibson-style cloning (Feeser and Wolberger, 2008). In short, plasmid 471 was linearized by PCR to allow cloning of homologous Rap1 C terminus fragments containing point and patch mutations. Rap1 plasmids were then linearized by digestion with PmeI and integrated at the *HIS3* locus.

The mCherry-YFP *pPPT1-pSUT129* fluorescent reporter plasmid was described previously (gift from Sebastian Marquardt) (Marquardt et al., 2014). The Rap1 transcription factor binding sites from the *RPL43B* promoter were cloned into unique SspI (proximal) or XmnI (distal) restriction sites. The *PPT1-SUT129* locus was replaced by digesting the plasmid with EcoRI and integrating the reporter construct by transformation as described above. All plasmids are listed in Table S5.

#### Fluorescence Microscopy and Quantification

Cells were grown in YPD to the exponential phase and fixed with formaldehyde (3.7% w/v) for 15 min. Fixed cells were washed with phosphate-sorbitol buffer (0.1 M KP_i_ (pH 7), 0.05 M MgCl_2_, 1.2 M sorbitol), and resuspended in phosphate-sorbitol buffer before imaging. Imaging was performed using a 100x oil objective, NA 1.4, and SOLA SE light engine (Lumencor) on a Nikon Eclipse Ti-E imaging system (Nikon). We used 500 ms exposure time using GFP and mCherry filters to quantify YFP and mCherry levels, respectively. An ORCA-FLASH 4.0 camera (Hamamatsu) and NIS-Elements AR software (Nikon) were used to collect images.

Whole cell fluorescence signals were obtained for YFP and mCherry channels using ImageJ software (NIH) (Schneider et al., 2012). ROIs were manually drawn around the periphery of each cell. The mean intensity in each channel per cell was multiplied by the cell area to obtain mean signal. The signal for each channel was corrected for cell-free background fluorescence in a similar way. Auto-fluorescence signal was also determined as described for wild-type cells. For the analyses, 50 cells were quantified per sample.

#### Spot Growth Assay

Cells were grown to saturation in YPD media, then diluted to OD_600_ 0.4 in sterile water. Serial dilutions (5-fold) were spotted onto YPD agar plates in the presence of IAA or DMSO. Cells were incubated at 30°C for 2 days before imaging.

#### RNA Extraction

Yeast cells were collected from cultures by centrifugation, washed with sterile water, and snap-frozen in liquid nitrogen. RNA was extracted from yeast cell pellets using Acid Phenol:Chloroform:Isoamyl alcohol (125:24:1, Ambion) and precipitated in ethanol with 0.3 M sodium acetate. RNA was resuspended in DEPC-treated sterile water.

#### Northern Blot

Northern blots were performed as previously described (Chia et al., 2017). RNA samples were denatured in denaturation buffer (1 M deionized glyoxal, 50% v/v DMSO, 10 mM sodium phosphate (NaP_i_) buffer (pH 6.8)) at 70°C for 10 min. Denatured samples were mixed with loading buffer (10% v/v glycerol, 2 mM NaP_i_ buffer, 0.4% w/v bromophenol blue) and separated on an agarose gel (1.1% v/v agarose, 0.01 M NaP_i_ buffer) by electrophoresis for 2 hr at 80 Volts. Total RNA was transferred onto positively charged nylon membranes (GE Amersham Hybond N+) by capillary transfer and rRNA bands were visualized by methylene blue staining (0.02% w/v methylene blue, 0.3 M sodium acetate).

The membranes were blocked for at least 3 hr at 42°C in hybridization buffer (1% w/v SDS, 40% v/v deionized formamide, 25% w/v dextran sulfate, 58 g/L NaCl, 200 mg/L sonicated salmon sperm DNA (Agilent), 2 g/L BSA, 2 g/L polyvinyl-pyrolidone, 2 g/L Ficoll 400, 1.7 g/L pyrophosphate, 50 mM Tris pH 7.5) or ULTRAhyb Ultrasensitive Hybridization Buffer (Thermo Fisher Scientific) before hybridization. The radioactively labeled probes were synthesized using a Prime-it II Random Primer Labeling Kit (Agilent), 25 ng of target-specific DNA template, and dATP [α-^32^P] (Perkin-Elmer or Hartmann Analytic). The oligonucleotide sequences used to generate target-specific DNA templates for *IRT2*, *iMLP1*, and *SNR190* northern blot probes are found in Table S6. After overnight hybridization at 42°C, blots were washed for 30 min at 65°C with each of the following: 2X saline-sodium citrate (SSC) buffer, 2X SSC + 1% w/v SDS, 1X SSC + 1% SDS, and 0.5X SSC + 1% SDS. Membranes were exposed to phosphorimaging screens before scanning using Typhoon 9400, FLA 9500, or FLA 7000 instruments (GE Healthcare Life Sciences). For re-probing, membranes were washed with stripping buffer (1 mM Tris, 0.1 mM EDTA, 0.5% SDS) at 85°C until negligible residual signal remained on the membrane.

*IRT2*, *iMLP1*, and *SNR190* levels were estimated from northern blots using ImageJ (Schneider et al., 2012). The net intensity of each region of interest was determined by subtracting the mean background intensity of the areas immediately above and below the region of interest from the intensity of the main band(s). Signals were first normalized to *SNR190* levels, and then further normalized to a specific band on the same membrane.

#### Western Blot

Western blots were performed as previously described (Chia et al., 2017). Protein extracts were prepared using the trichloroacetic acid (TCA) extraction protocol. Cells were collected by centrifugation and re-suspended in cold 5% w/v TCA for at least 10 min. Samples were washed with acetone, then completely air-dried. Cells were resuspended with protein breakage buffer (50 mM Tris (pH 7.5), 1 mM EDTA, 2.75 mM dithiothreitol (DTT)) and disrupted using 0.5 mm glass beads and a Mini Beadbeater (Biospec). Two volumes of protein extract were mixed with 1 volume of SDS-PAGE sample buffer (187.5 mM Tris (pH 6.8), 6.0% v/v β-mercaptoethanol, 30% v/v glycerol, 9.0% v/v SDS, 0.05% w/v Bromophenol blue) and denatured at 95°C for 5 min. After SDS-polyacrylamide gel electrophoresis (4%–20% gradient), proteins were transferred onto PVDF membranes. The membranes were blocked in blocking buffer (1% w/v BSA, 1% w/v non-fat powdered milk in phosphate buffered saline with 0.01% v/v Tween-20 (PBST) buffer) before incubation with primary antibodies in blocking buffer overnight at 4°C. Membranes were washed in PBST buffer and incubated with anti-mouse or anti-rabbit IgG HRP-linked antibodies in blocking buffer. Protein levels were detected using Amersham ECL Prime detection reagent and an Amersham Imager 600 instrument (GE Healthcare).

#### Antibodies

The following antibodies were used for western blotting (also see Key Resources Table). Anti-V5 mouse monoclonal IgG_2A_ (1:2000, Thermo Fisher Scientific R96025, previously Invitrogen 46-0705), anti-hexokinase rabbit (1:8000, US Biological H2035), anti-Myc tag mouse monoclonal (1:2000, Merck Millipore CAT 05-724 Lot DAM1764400), anti-HA tag mouse IgG (1:2000, clone 12CA5), anti-FLAG tag monoclonal mouse IgG_1_ (1:2000, Sigma-Aldrich F3165), anti-mouse IgG antibody HRP-linked (1:10000, GE healthcare NA931V5), anti-rabbit IgG antibody HRP-linked (1:10000, GE healthcare NA934V).

#### Chromatin Immunoprecipitation

ChIP experiments were performed as previously described (Chia et al., 2017). In short, cells were fixed with 1% v/v formaldehyde for 20 min at room temperature and reactions were quenched with glycine (100 mM). Cells were washed once with FA lysis buffer (0.05 M HEPES-KOH (pH 7.5), 0.15 M NaCl, 0.001 M EDTA (pH 8), 1% v/v Triton X-100, 0.1% w/v sodium deoxycholate, 0.1% w/v SDS) and snap-frozen with liquid nitrogen. Cell pellets were disrupted using a Mini Beadbeater and zirconia/silica beads (0.5 mm, Biospec), and cross-linked chromatin extracts were sheared by sonication using a Bioruptor (Diagenode, 9 cycles of 30 s on/off, high intensity). Extracts were incubated for 2 hr at room temperature with 20 μL of anti-V5 antibodies conjugated to agarose beads (Sigma-Aldrich). Subsequently, reverse-crosslinking was performed in TE-SDS buffer (10 mM Tris (pH 8), 1 mM EDTA, 1.0% w/v SDS) at 65°C overnight, samples were treated with Proteinase K (Thermo Fisher Scientific), and DNA fragments were purified by spin column (Machery-Nagel). ChIP signals at *RPL43B* and *RPL40B* promoters were determined by qPCR using PowerUp SYBR Green Master Mix (Thermo Fisher Scientific) and a QuantStudio 3 instrument (Applied Biosystems). As a negative control, we used a primer pair directed to the *ACT1* ORF 3′ end. The oligonucleotide sequences used for ChIP experiments are in Table S6.

#### Single Molecule RNA FISH

Single molecule RNA fluorescence *in situ* hybridization (FISH) was performed as previously described (Moretto et al., 2018). In short, cells were fixed with formaldehyde overnight, treated with zymolyase and further fixed in 80% v/v ethanol. Subsequently, cells were hybridized with fluorophore-labeled probes (Biosearch Technologies) directed to *IME1* (AF594) and the internal control *ACT1* (Cy5) (Dyes, Thermo Fisher Scientific). Cells were imaged using a 100x oil objective, NA 1.4, and SOLA SE light engine (Lumencor) on a Nikon Eclipse Ti-E imaging system (Nikon). DIC, DAPI, AF594 (*IME1*), and Cy5 (*ACT1*) images were collected every 0.3 micron (20 stacks) using an ORCA-FLASH 4.0 camera (Hamamatsu) and NIS-Elements AR software (Nikon). ImageJ was used to generate maximum intensity Z projections of the images (Schneider et al., 2012). Subsequently, StarSearch software (Raj laboratory, University of Pennsylvania, http://rajlab.seas.upenn.edu/StarSearch/launch.html) was used to quantify transcripts in single cells. Comparable thresholds were used to count RNA foci in single cells. Only cells positive for the internal control *ACT1* were quantified for analysis (n = 139 cells).

#### RNA Sequencing Library Preparation

Total RNA from yeast was incubated with rDNase (Machery-Nagel) and column purified (Machery-Nagel) prior to sequencing library preparation. 1 μg of intact yeast total RNA was depleted of ribosomal RNA (rRNA) using a commercial kit (Illumina RiboZero Gold rRNA Removal Kit (Yeast)) for total RNA sequencing and 500 ng of RNA was used for polyadenylated (polyA) RNA sequencing. Libraries were prepared using the TruSeq Stranded Total RNA kit or TruSeq stranded mRNA kit (Illumina) according to the manufacturer’s instructions (10 or 13 PCR cycles). Each library was sequenced on the HiSeq 2500 or 4000 platform (Illumina) and generated ∼45 million 101 bp strand-specific paired-end reads per sample, on average.

#### TSS Sequencing Library Preparation

To obtain libraries representing the 5′ ends of polyadenylated and capped transcripts (TSS-seq), approximately 7-9 μg of poly(A)^+^ RNA together with *in vitro* spike-ins was first subjected to zinc-mediated fragmentation (Ambion) at 70°C. The reaction was subsequently cleaned up using RNeasy MinElute columns (QIAGEN) to isolate RNA fragments with mode length of ∼200 nucleotides. These fragments were incubated with shrimp alkaline phosphatase (rSAP, NEB) at 37°C to remove the 5′ phosphate groups of non-capped fragments, followed by acid phenol/chloroform extraction and ethanol precipitation as described above. With the exception of a “no decapping” control sample from wild-type (WT, FW629) cells, dephosphorylated fragments were next treated with Cap-Clip Acid Pyrophosphatase (CellScript) to remove the 5′-terminal caps from fragments representing the bona fide 5′ ends of transcripts. After another round of acid phenol/chloroform extraction and ethanol precipitation, all samples were treated with T4 RNA ligase 1 (NEB) to introduce a custom adaptor sequence to the 5′ uncapped ends of fragments. Excess adapters were removed via a column clean up step. First strand cDNA synthesis was performed using Superscript IV Reverse Transcriptase (Thermo Fisher Scientific), and second strand synthesis was performed using a KAPA HiFi HotStart ReadyMixPCR Kit (KAPA Biosystems) after RNase H (NEB) and RNase cocktail digestion (Ambion). Double-stranded cDNA was quantified by Qubit fluorometric quantitation (Thermo Fisher Scientific) and used as inputs for library preparation using a KAPA Hyper Prep Kit (KAPA Biosystems) and KAPA Single-Indexed adapters for Illumina platforms (KAPA Biosystems). Libraries were quantified by Qubit and sequenced on the HiSeq 4000 platform (Illumina), and typically generated ∼39 million 76bp strand-specific single-end reads per sample. ∼16 million single-end reads were generated from the “No decapping” control library.

#### Differential Expression Analysis

Adaptor trimming was performed with cutadapt (version 1.9.1) with parameters “–minimum-length=25–quality-cutoff=20 -a AGATCGGAAGAGC -A AGATCGGAAGAGC” (Martin, 2011). The RSEM package (version 1.3.0) (Li and Dewey, 2011) in conjunction with the STAR alignment algorithm (version 2.5.2a) (Dobin et al., 2013) was used for the mapping and subsequent gene-level counting of the sequenced reads with respect to all *S. cerevisiae* genes downloaded from the Ensembl genome browser (assembly R64-1-1, release 90). The parameters used were “–star-output-genome-bam–forward-prob 0,” and all other parameters were kept as default. Differential expression analysis was performed with the DESeq2 package (version 1.12.3) (Love et al., 2014) within the R programming environment (version 3.3.1).

A list of experimentally determined Rap1 sites was obtained from a high-resolution ChIP-exo dataset (Rhee and Pugh, 2011). For differential expression analysis with varying window sizes (e.g., ± 50 bp to ± 500 bp), sites within 500 bp of chromosome ends were removed. To determine the list of 141 well annotated Rap1-regulated genes, we combined lists of ribosomal protein (RP) genes (Reja et al., 2015) and previously identified Rap1-regulated glycolytic pathway genes (Lieb et al., 2001), and removed the RP genes regulated by Abf1 (instead of Rap1). We manually assigned the corresponding promoter Rap1 site to each Rap1-regulated gene from the ChIP-exo dataset. If ChIP-exo coordinates were missing, the Rap1 motif coordinate identified from from Lieb et al. was assigned instead (Lieb et al., 2001). STAR genomic alignments were filtered to only include those that were unspliced, primary, uniquely mapped, properly paired, and had a maximum insert size of 500bp. Fragment counts within specified windows (e.g., ± 100 bp) around the 564 Rap1 sites (1128 intervals total, Watson and Crick strand alignments were assigned to separate intervals) were obtained using the featureCounts tool from the Subread package (version 1.5.1) (Liao et al., 2014). The parameters used were “-O–minOverlap 1–nonSplitOnly–primary -s 2 -p -B -P -d 0 -D 600 -C.” Windows on separate strands were treated as separate intervals for all strand-specific RNA-seq experiments, and only reads which overlapped with the corresponding strand and interval were counted. Differential expression analysis around Rap1 binding sites was performed as described in the section above, however, the DESeq2 size factors with respect to the transcriptome were used to normalize the per-sample counts. The same strategy was employed to perform analysis for promoter regions of Ume6-regulated genes (McKnight et al., 2016). Ume6 sites were approximated −250 bp relative to the annotated start of Ume6-regulated genes. The ggplot2 package (version 2.2.1) was used within the RStudio programming environment (version 3.4.0) to generate violin and box-and-whisker plots. The calculated log_2_(Fold change) values from DESeq2 analysis were plotted using the geom_violin (scale = “count”) and geom_boxplot functions (outlier data points not shown in boxplot but included in violin plot). Volcano and scatterplots were generated using the geom_point function in ggplot2 or Graphpad Prism (version 7.02). Screenshots of RNA-seq and TSS-seq data were taken using the Integrative Genomics Viewer (Broad Institute, version 2.3.75).

#### TSS-Seq Analysis

Adaptor trimming was performed with cutadapt (version 1.9.1) (Martin, 2011) with parameters “–minimum-length=20–quality-cutoff=20 -a AGATCGGAAGAGC.” The custom 5′ adaptor sequence specific to the protocol was removed by re-running cutadapt with the parameters “–minimum-length=20–quality-cutoff=20 -g GCACTCTGAGCAATACC,” and only the reads containing the adaptor sequence were used for further analysis. BWA (version 0.5.9-r16) (Li and Durbin, 2009) using default parameters was used to perform the read mapping to the *S. cerevisiae* genome (assembly R64-1-1, release 90). Uniquely mapped alignments corresponding to the sense and antisense strands were obtained using SAMtools view (version 1.3.1) by using the flags “-q 1 -F 20” and “-q 1 -f 16,” respectively (Li et al., 2009). BedGraph coverage tracks representing the TSS-seq signal per million mapped reads were generated using BEDTools genomeCoverageBed (version 2.26.0) (Quinlan and Hall, 2010) with the parameters “-bg −5 -scale <SCALE_FACTOR>.” BedGraph files were converted to bigWig using the wigToBigWig binary available from the UCSC with the “-clip” parameter (Kent et al., 2010). Coverage tracks from three biological replicates for each sample were merged for plotting. TSS annotations were obtained from Ensembl assembly R64-1-1, release 90 and annotated SMORE-seq TSSs described previously (Park et al., 2014). To calculate TPM values for each TSS, TSS-seq counts were obtained by quantifying the abundance of reads with the 1^st^ transcribed 5′ nucleotide within ± 75 bp of annotated TSSs (Park et al., 2014), on the respective strand. For the analysis in Figure 4C, the closest cryptic TSS to the Rap1 binding site was annotated manually, and distance was measured from the Rap1 binding site to the mode TSS cluster peak. Differential expression analysis was performed using DESeq2, normalized by sequencing depth. A log_2_(fold change) value > 1 (fold change > 2), comparing *RAP1-AID* +IAA over wild-type cells, was considered an increase.

#### ChIP-Seq and MNase-Seq Analysis

Publicly available datasets for Sth1 ChIP-seq (GEO: GSE56994) (Lopez-Serra et al., 2014), Sth1 MNase ChIP-seq (GEO: GSE65594) (Parnell et al., 2015), and MNase-seq (GEO: GSE73337) (Kubik et al., 2015) described previously were obtained from GEO. ChIP-seq and MNase-seq reads were adaptor-trimmed using cutadapt as specified previously. Genome-wide mapping of the trimmed reads was performed with BWA (version 0.5.9-r16) (Li and Durbin, 2009) using default parameters. Single-end ChIP-seq alignments were filtered to remove duplicate and multi-mapped reads. Paired-end MNase-seq alignments were filtered to only include those that were properly paired, uniquely mapped, had a maximum of two mismatches in either read, and an insert size within the range 120 - 200 bp. Genome-wide nucleosome coverage profiles were obtained using the DANPOS2 dpos (version 2.2.2) (Chen et al., 2013) command with parameters “–span 1–smooth_width 20–width 40–count 1000000.”

#### Immunuprecipication of Chromatin-Bound Rap1 and Mass Spectrometry

Chromatin extracts were prepared as previously described (van Werven et al., 2008). In short, cells were disrupted using 0.5 mm glass beads in nuclear isolation buffer (NIB: 250 mM sucrose, 10 mM MgCl_2_, 20 mM HEPES (pH 7.8), 0.1% v/v Triton X-100, 5 mM β-mercaptoethanol, 1X cOmplete protease inhibitor (Roche)). The pellet was then collected by centrifugation (27000 x *g*, 15 min, 4°C), washed once in NIB buffer, centrifuged again, and resuspended in 4.5 mL NIB buffer with 2 mM CaCl_2_. Samples were treated with 3000 U of micrococal nuclease (MNase, NEB) for 4 min at 30°C and reactions were stopped by addition of EDTA to 10 mM, then transferred onto ice. The concentration of NaCl was adjusted to 150 mM and samples were clarified by centrifugation at 16000 x *g* for 10 min at 4°C. The supernatant was taken as the chromatin extract.

Anti-V5 tag immunoprecipitation was performed on approximately 15 mg of chromatin extract from cells expressing full-length Rap1-V5 (FL, FW5420), Rap1(ΔAD, FW5424)-V5, Rap1(Δ631-696, FW5396)-V5, or containing an empty vector control (Untagged, FW5399). 100 μL of anti-V5 agarose affinity gel antibody (Sigma-Aldrich) was incubated with chromatin extracts for 4 hr at 4°C. Agarose beads were washed 5 times with 1 mL NIB wash buffer (NIB buffer with 350 mM NaCl) and proteins were eluted in SDS-PAGE sample buffer by heating at 95°C for 5 min.

Eluted proteins were subjected to SDS-PAGE, migrated approximately 1 cm into the gel (12% NuPAGE, Invitrogen), and stained with InstantBlue Protein Stain (Expedeon). Proteins were in-gel digested using trypsin, and peptides were analyzed with an Orbitrap-Fusion Lumos mass spectrometer coupled to an Ultimate3000 HPLC equipped with an EASY-Spray nanosource (Thermo Fisher Scientific). Label-free quantification (LFQ) was performed using MaxQuant software (v1.6.01) (Cox and Mann, 2008). Perseus software (version 1.4.0.2) was used for further statistical processing of the proteingroup.txt output table (Tyanova et al., 2016). LFQ intensities were log_2_ transformed, and the dataset was filtered for proteins having at least three values in at least one group (each group consisting of triplicate injections). The remaining missing values were imputed using default Perseus settings by drawing from a simulated noise distribution with a down shift of 1.8 and a width of 0.3 compared with the log_2_ LFQ intensity distribution. Two-sample t tests were performed with a permutation-based FDR set at 0.05. Proteins that were enriched >2-fold with p < 0.05 (comparing Full-Length versus Untagged Control samples) were subjected to SGD Gene Ontology Slim Mapper Process Analysis (https://www.yeastgenome.org/cgi-bin/GO/goSlimMapper.pl). Volcano plots were generated using Graphpad Prism (version 7.02). The processed mass spectrometry data are available in Table S2.

For the GO-Slim terms in Figure 6C, proteins that were enriched >2-fold with p < 0.05 (unpaired two-sample t test) were used for the analysis (n = 289 proteins) in the SGD Yeast GO-Slim Process Mapper. 13 proteins that were ambiguously assigned to genes were excluded from the analysis.

#### Oligonucleotides Used in This Study

A table of oligonucleotides used in this study is available in Table S6.

### Quantification and Statistical Analysis

Details of statistical tests used, sample number, and number of independent experiments are included in the relevant figure legends. p values were calculated using the Students’ t test, with p < 0.05 considered significant. Error bars are described in individual figure legends as ± standard error of the mean (±SEM) or 95% confidence intervals. Standard box-and-whisker plots were generated showing the median value (horizontal line), lower and upper quartiles (lower and upper hinges), and lowest and highest values (whiskers, within 1.5 times interquartile range).

### Data and Software Availability

The accession number for the RNA sequencing and TSS sequencing data reported in this paper is GEO: GSE110004.

## Supplemental Information

Document S1. Figures S1–S7 and Tables S1, S3, S5, and S6

Table S2. Chromatin-Bound Rap1 Mass Spectrometry Data, Related to Figure 6

Table S4. Strain Genotypes, Related to STAR Methods

Document S2. Article plus Supplemental Information
