# Supplementary material for: Repression of Divergent Noncoding Transcription by a Sequence-Specific Transcription Factor
Source: Mol Cell. 2018 Dec 20;72(6):942–954.e7. doi: 10.1016/j.molcel.2018.10.018 (PMC6310685; doi:10.1016/j.molcel.2018.10.018)
Supplement: Document S1. Figures S1–S7 and Tables S1, S3, S5, and S6 [file mmc1.pdf]

Molecular Cell, Volume *72*

Supplemental Information

Repression of Divergent Noncoding Transcription

by a Sequence-Specific Transcription Factor

Andrew C.K. Wu, Harshil Patel, Minghao Chia, Fabien Moretto, David Frith, Ambrosius P. Snijders, and Folkert J. van Werven

Figure S1. Wu *et al.*

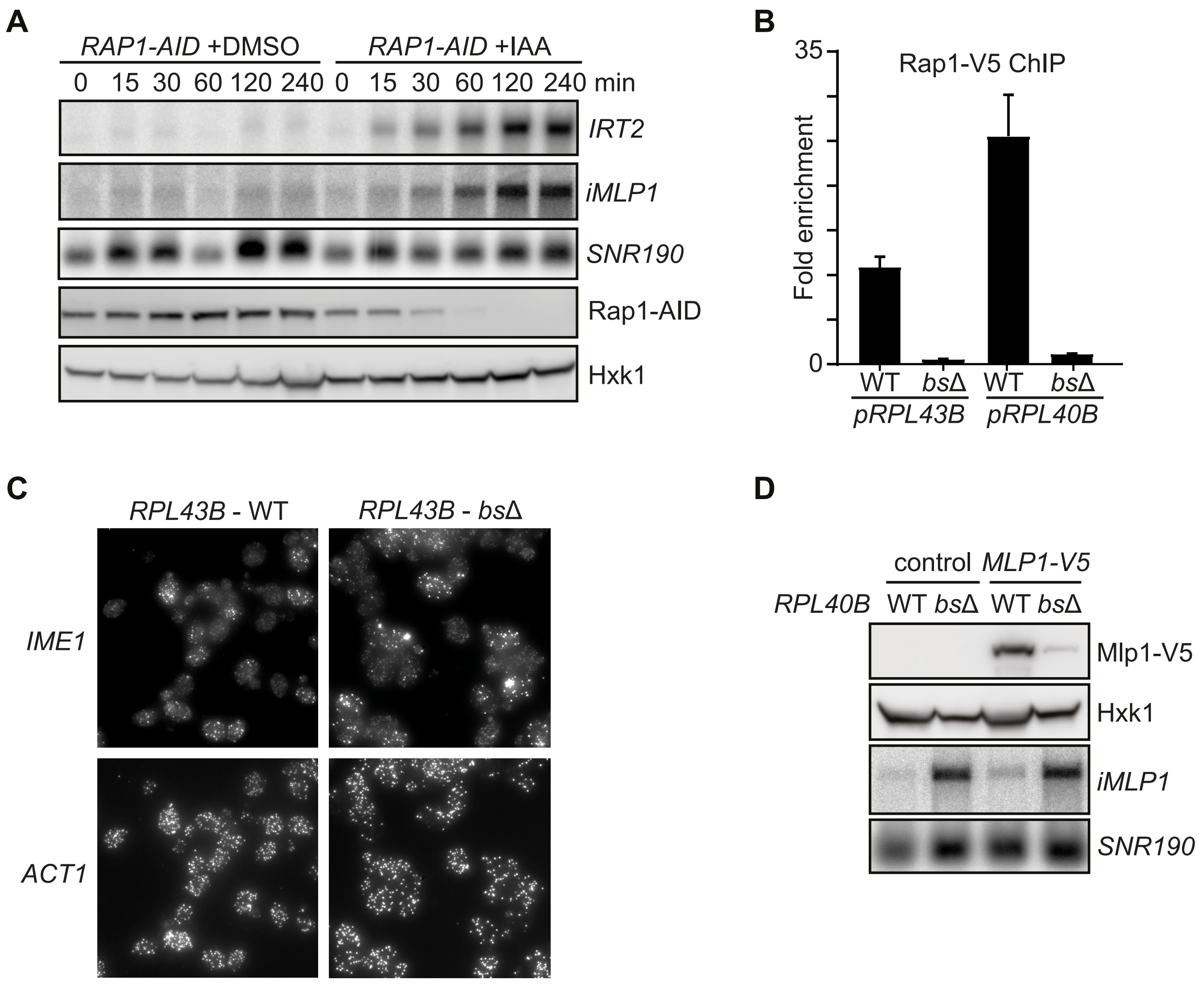

Figure S1. Rap1 prevents expression of noncoding RNAs, Related to Figure 1. (**A**) Northern blots and western blots corresponding to Figure 1D, except samples were included from *RAP1-AID* cells (FW3877) treated with DMSO or IAA at equivalent time points. *IRT2*, *iMLP1*, and *SNR190* transcripts were detected using labelled northern blot probes as described in Figure 1D, and Rap1-V5-AID and Hxk1 were detected using anti-V5 and anti-Hxk antibodies. (**B**) Rap1 binding at *RPL43B* and *RPL40B* promoters determined by chromatin immunoprecipitation. Cells harbouring Rap1-V5 (FW4732), *RPL43B*-bsΔ Rap1-V5 (FW4734), and *RPL40B*-bsΔ Rap1-V5 (FW6228) were grown to exponential phase. Cell were crosslinked with formaldehyde, chromatin extracts were prepared, and anti-V5 antibodies were used to immunoprecipitate Rap1-V5 bound DNA fragments. Rap1 binding at *RPL43B* and *RPL40B* promoters was measured by qPCR, and the signals were normalized over *ACT1* gene 3' end. The mean fold enrichment from three independent experiments plus the standard error of the mean (+SEM) is plotted. (**C**) Representative single-molecule RNA fluorescence in-situ hybridization (RNA FISH) images corresponding to Figure 1H. Single spots corresponding to individual *IME1* (AF594) or *ACT1* (Cy5) mRNA transcripts were counted in diploid wild-type (FW631) or *RPL43B*-bsΔ (FW6139) cells immediately after shifting to SPO medium. (**D**) Northern blots and western blots corresponding to Figure 1D, showing the induction of *iMLP1* and reduction of Mlp1 expression when the *RPL40B* promoter Rap1 site is deleted. Wild-type (FW629), *RPL40B*-bsΔ (FW4141), or *MLP1* tagged with V5 epitope tag (FW4122) and *MLP1-V5 RPL40B*-bsΔ (FW4120) cells were grown to exponential growth and samples were collected. Expression of Hxk1 and *SNR190* are shown for western and northern blot loading controls, respectively.

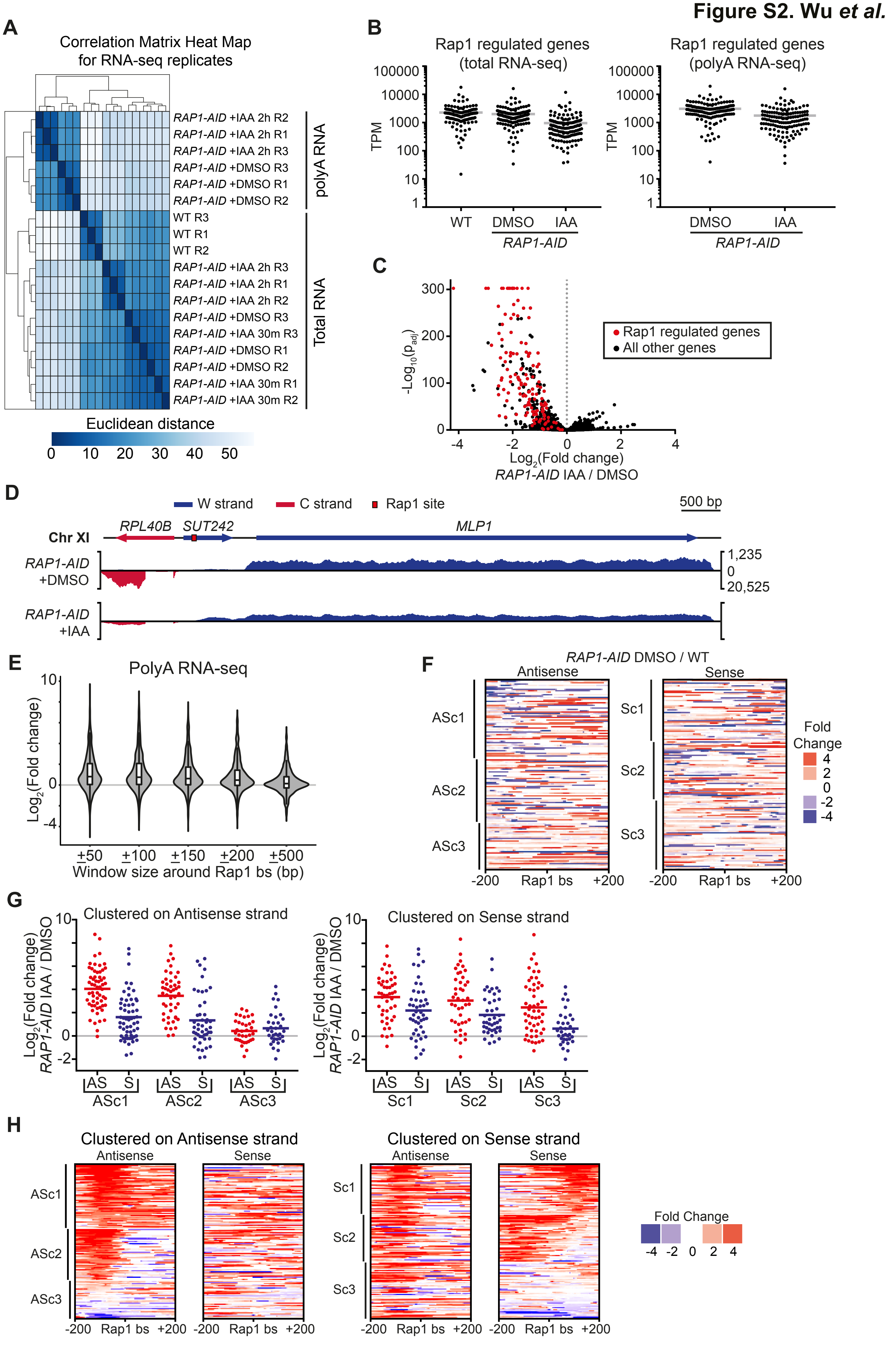
A
Correlation Matrix Heat Map for RNA-seq replicates
RAP1-AID +IAA 2h R2
RAP1-AID +IAA 2h R1
RAP1-AID +IAA 2h R3
RAP1-AID +DMSO R3
RAP1-AID +DMSO R1
RAP1-AID +DMSO R2
WT R3
WT R1
WT R2
RAP1-AID +IAA 2h R3
RAP1-AID +IAA 2h R1
RAP1-AID +IAA 2h R2
RAP1-AID +DMSO R3
RAP1-AID +IAA 30m R3
RAP1-AID +DMSO R1
RAP1-AID +DMSO R2
RAP1-AID +IAA 30m R1
RAP1-AID +IAA 30m R2
polyA RNA
Total RNA
Euclidean distance
0 10 20 30 40 50
B
Rap1 regulated genes (total RNA-seq)
Rap1 regulated genes (polyA RNA-seq)
TPM
WT
DMSO
IAA
RAP1-AID
C
-Log10(padj)
Rap1 regulated genes
All other genes
Log2(Fold change)
RAP1-AID IAA / DMSO
D
W strand
C strand
Rap1 site
500 bp
Chr XI
RPL40B
SUT242
MLP1
RAP1-AID +DMSO
RAP1-AID +IAA
1,235
0
20,525
E
PolyA RNA-seq
Log2(Fold change)
±50 ±100 ±150 ±200 ±500
Window size around Rap1 bs (bp)
F
RAP1-AID DMSO / WT
Antisense
Sense
ASc1
ASc2
ASc3
Sc1
Sc2
Sc3
Fold Change
-200 Rap1 bs +200
G
Clustered on Antisense strand
Clustered on Sense strand
Log2(Fold change) RAP1-AID IAA / DMSO
AS S
ASc1 ASc2 ASc3
Sc1 Sc2 Sc3
H
Clustered on Antisense strand
Clustered on Sense strand
Antisense
Sense
Fold Change
-4 -2 0 2 4
-200 Rap1 bs +200

Figure S2. Rap1 represses divergent noncoding transcription, Related to Figure 2.

(A) Correlation matrix heat map for RNA-seq biological replicate samples, showing the Euclidean distance between the samples across the genome (based on all genes). **(B)** Scatter plots showing distribution of expression for Rap1 regulated genes (n = 141) in wild-type (FW629), and *RAP1-AID* (FW3877) cells after DMSO or IAA treatment. Separate plots shown for total (rRNA depleted) and polyadenylated (polyA) RNA-seq. Each dot represents transcripts per million (TPM) for each gene, shown on the y-axis (exponential scale). Gray lines indicate mean values for each group. **(C)** Volcano plot showing that the expression of Rap1-regulated genes is decreased upon Rap1 depletion. On the y-axis the false discovery rate adjusted *p*-value ($-Log_{10}(p_{adj})$) is plotted, and on the x-axis the fold change is displayed (Log_2(Fold change)). *RAP1-AID* (FW3877) cells after IAA or DMSO treatment were compared. For the analyses (n = 7126) genes were used and in red (n = 141) Rap1 regulated genes are highlighted. Data are calculated from three independent experiments. **(D)** Genome browser tracks showing example of divergent noncoding RNA *iMLP1* repressed by Rap1, adjacent to the *RPL40B* locus. *RAP1-AID* cells (FW3877) were grown to exponential phase and were treated with DMSO or IAA for 2 hours. Samples were taken and processed for total RNA-seq. The normalized reads are shown on the y-axis for the Watson (W, blue) and Crick (C, red) strands. **(E)** Violin and box-and-whisker plots, as described in Figure 2C, showing distribution of expression changes around Rap1 sites (n = 564) for polyadenylated (polyA) transcripts. The fold change in expression comparing *RAP1-AID* +IAA versus *RAP1-AID* +DMSO treated cells is plotted on the y-axis. Signals for Watson and Crick strands were computed separately, resulting in n = 1128 data points for window sizes of 50, 100, 150, 200, and 500 bp up- and downstream of the Rap1 binding site. **(F)** Control heat maps showing the changes in RNA expression on the antisense and sense strands around n = 141 promoter Rap1 sites. For the analysis *RAP1-AID* (FW3877) DMSO treated cells were compared to wild type control (WT, FW629) cells. Promoters were clustered and ordered as described in Figure 2F. **(G)** Scatter plots showing RNA expression changes 100 bp up- and downstream of Rap1 binding sites ($\pm$100 bp) after Rap1 depletion as described in Figure 2E. Data are separated by antisense (left) or sense (right) clusters. Fold change values were calculated for *RAP1-AID* (FW3877) +IAA or +DMSO treated cells from three independent experiments. Number of promoters in each cluster: ASc1 (n = 59), ASc2 (n = 47), ASc3 (n = 35), Sc1 (n = 46), Sc2 (n = 43), Sc3 (n = 52). Horizontal lines, mean values. **(H)** Heat maps showing the changes in RNA expression on the antisense and sense strands, similar to Fig. 2F except that both strands are shown. Clustered and ordered based on antisense (ASc1-3) or sense (Sc1-3) strand signals using k-means clustering (k = 3).

Figure S3. Wu *et al.*

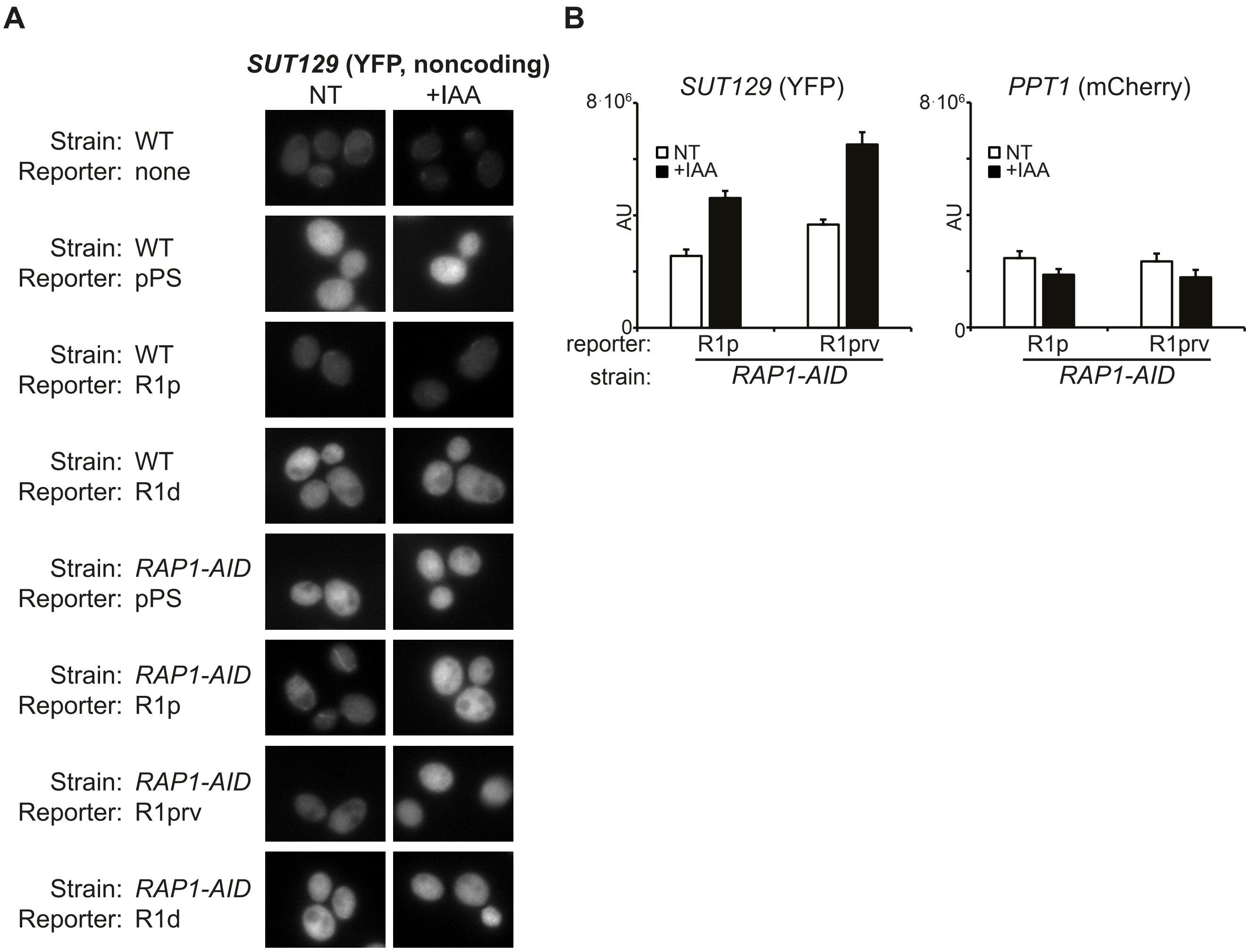

Figure S3. A proximal Rap1 motif is required and sufficient to repress divergent transcription, Related to Figure 3.

(A) Representative images showing *SUT129* promoter activity (YFP, noncoding), as described in Figure 3. Cells were grown in YPD and samples were either not treated (NT) or treated with 500 μM IAA (+IAA) for four hours. The following cells were fixed and imaged: Wild-type cells harbouring no reporter (FW629), control reporter (pPS, FW6407), reporter with proximal Rap1 motifs (R1p, FW6895), reporter with distal Rap1 motifs (R1d, FW7253), *RAP1-AID* cells harbouring control reporter (pPS, FW6208), reporter with proximal Rap1 motifs (R1p, FW6206), reporter with proximal Rap1 motifs in reverse orientation (R1prv, FW6204), reporter with distal Rap1 motifs (R1d, FW6408) **(B)** Orientation of the Rap1 motifs does not affect repression of divergent noncoding transcription. *RAP1-AID* cells harbouring the reporter with R1p (FW6206), or R1prv (FW6204) were quantified for *SUT129* (YFP, noncoding) and *PPT1* (mCherry, coding) promoter activity. Mean signals corrected for background (AU, arbitrary units) are plotted plus 95% confidence intervals (error bars). N = 50 cells were quantified per sample.

Figure S4. Wu *et al.*

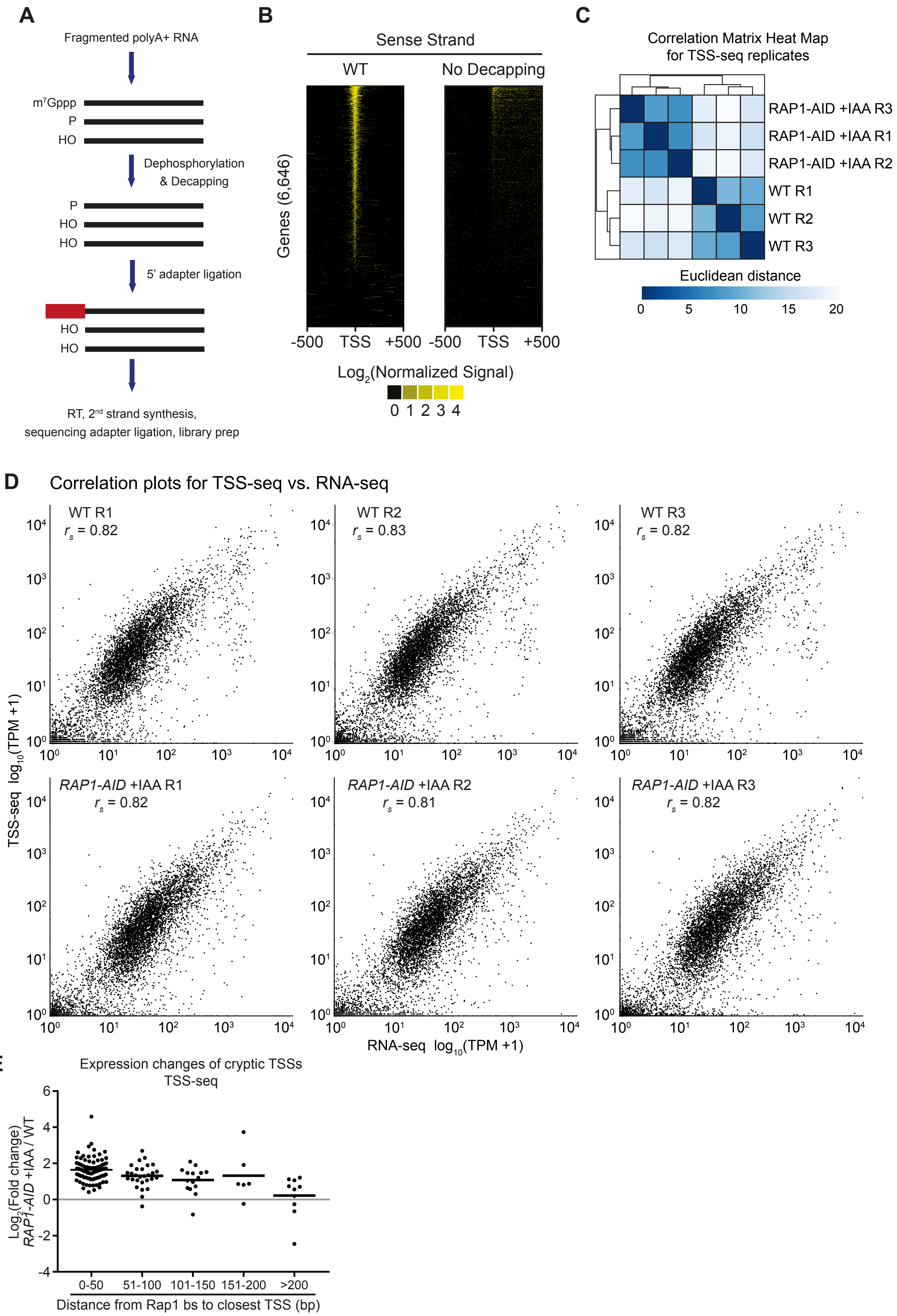

Figure S4. Rap1 represses divergent transcription initiation near its binding site, Related to Figure 4.

(A) Diagram of TSS sequencing (TSS-seq) protocol. Polyadenylated RNA was isolated and subjected to fragmentation, dephosphorylation, and decapping. A custom adapter sequence was ligated to 5' uncapped fragment ends. Samples then underwent reverse transcription, second strand synthesis, and library preparation for Illumina sequencing. See STAR methods for details. **(B)** Heat map of normalized TSS-seq signals for 6,646 *S. cerevisiae* genes in wild-type (FW629) cells (left), aligned to annotated transcription start sites (TSSs). A colour scale displays the range of normalized signals (Log_2 scale). Normalized counts from 3 independent experiments were merged for the plot. A "no decapping" control sample is shown where samples were not treated with decapping enzyme to enrich for 5' capped transcript ends. **(C)** Correlation matrix heat map for TSS-seq biological replicate samples, showing the Euclidean distance between the samples across the genome. Correlations are based on $\pm$75 bp windows centered on annotated TSSs for all genes (Park et al., 2014). **(D)** Scatter plots showing correlation between TSS-seq and RNA-seq data. TSS-seq counts were obtained from each gene by quantifying the abundance of reads with the 1st transcribed 5' nucleotide within $\pm$75 bp of annotated TSSs (Park et al., 2014), on the respective strand. These were converted to TPM values and plotted (TSS-seq, y-axis) against RNA-seq TPM values (x-axis) for the corresponding gene. Individual replicate comparisons are plotted separately. r_S, spearman's correlation coefficient. **(E)** Scatter plots showing changes in expression of cryptic TSSs near promoter Rap1 sites, comparing *RAP1-AID* +IAA versus wild-type control samples. TSSs were classified into bins of 50 bp, increasing in distance to the promoter Rap1 binding site. Fold change values were calculated from three independent experiments. Horizontal lines, mean values.

Figure S5. Wu *et al.*

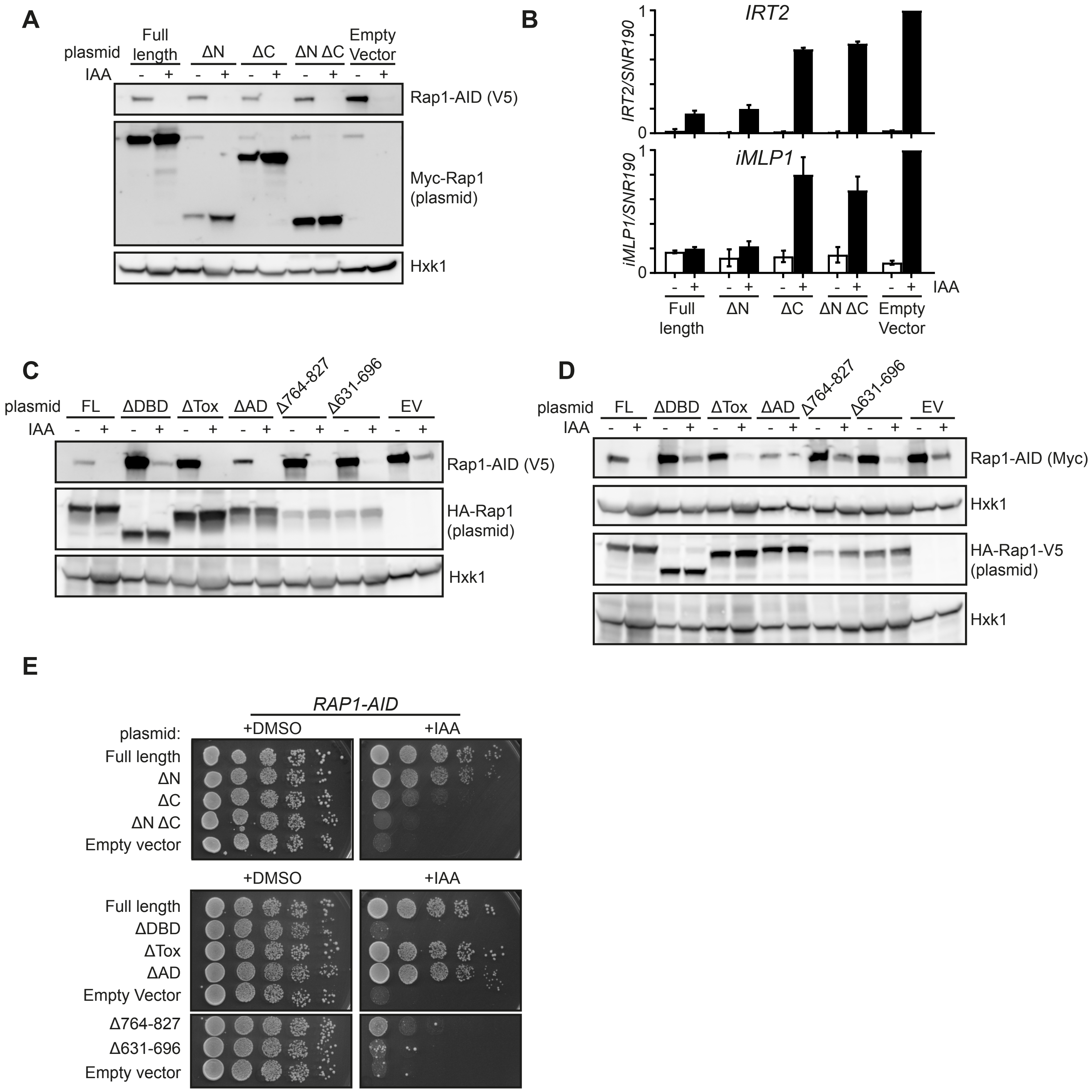

Figure S5. The Rap1 C-terminal domain contributes to repression of divergent noncoding transcription, Related to Figure 5.

(A) Expression of Rap1 truncation mutants, corresponding to Figure 5B. *RAP1-AID* cells expressing single copy integration vectors harboring full-length (FL) (FW5129), N-terminal deletion (ΔN) (FW5133), C-terminal deletion (ΔC) (FW5138), and ΔN ΔC (FW5141) Rap1*,* or empty vector (EV, FW5145). Samples were collected from cells before and 2 hours after treatment with IAA. AID-tagged Rap1 protein, Myc-tagged truncated Rap1 proteins, and Hxk1 (loading control) were detected using anti-V5, Myc, and Hxk antibodies, respectively. **(B)** Quantification of *IRT2* and *iMLP1* expression described in Figure 5B. Samples from two independent experiments were processed and the mean values plus standard error of the mean are displayed (±SEM). Signals for *IRT2* and *iMLP1* were normalized over *SNR190*, and to control for technical variation between experiments and blots, the normalized signal for Rap1-V5-AID +IAA containing empty vector was set to 1. **(C)** Expression of Rap1 domain mutants described in Figure 5C. *RAP1-AID* cells expressing single copy integration vectors harboring full-length Rap1 (FL) (FW4948), DNA-binding domain deletion (ΔDBD) (FW4950), toxicity domain deletion (ΔTox) (FW4952), activation domain deletion ΔAD (FW4954), residues 764 to 827 deleted (Δ764-827) (FW4958), residues 631 to 696 deleted (Δ631-696) (FW4960), or empty vector (EV) (FW5145). Samples were collected 0 and 2 hours after treatment with IAA. AID-tagged Rap1 protein, HA-tagged Rap1 domain mutant proteins, and Hxk1 (loading control) were detected using anti-V5, HA, and Hxk1 antibodies, respectively. **(D)** Similar to C, except that endogenous Rap1 was tagged with AID-Myc and the Rap1 domain mutant constructs also contain C-terminal V5 epitope tags, as described in Figure 5D. **(E)** Spot growth assay of Rap1 truncation and domain deletion constructs described in A and C. Cells were grown to saturation in YPD media overnight, then adjusted to equivalent optical density (OD_{600} = 0.4). Serial 5-fold dilutions were spotted onto YPD agar plates with IAA or DMSO. Plates were incubated at 30 °C for 2 days before imaging.

Figure S6. Wu *et al.*

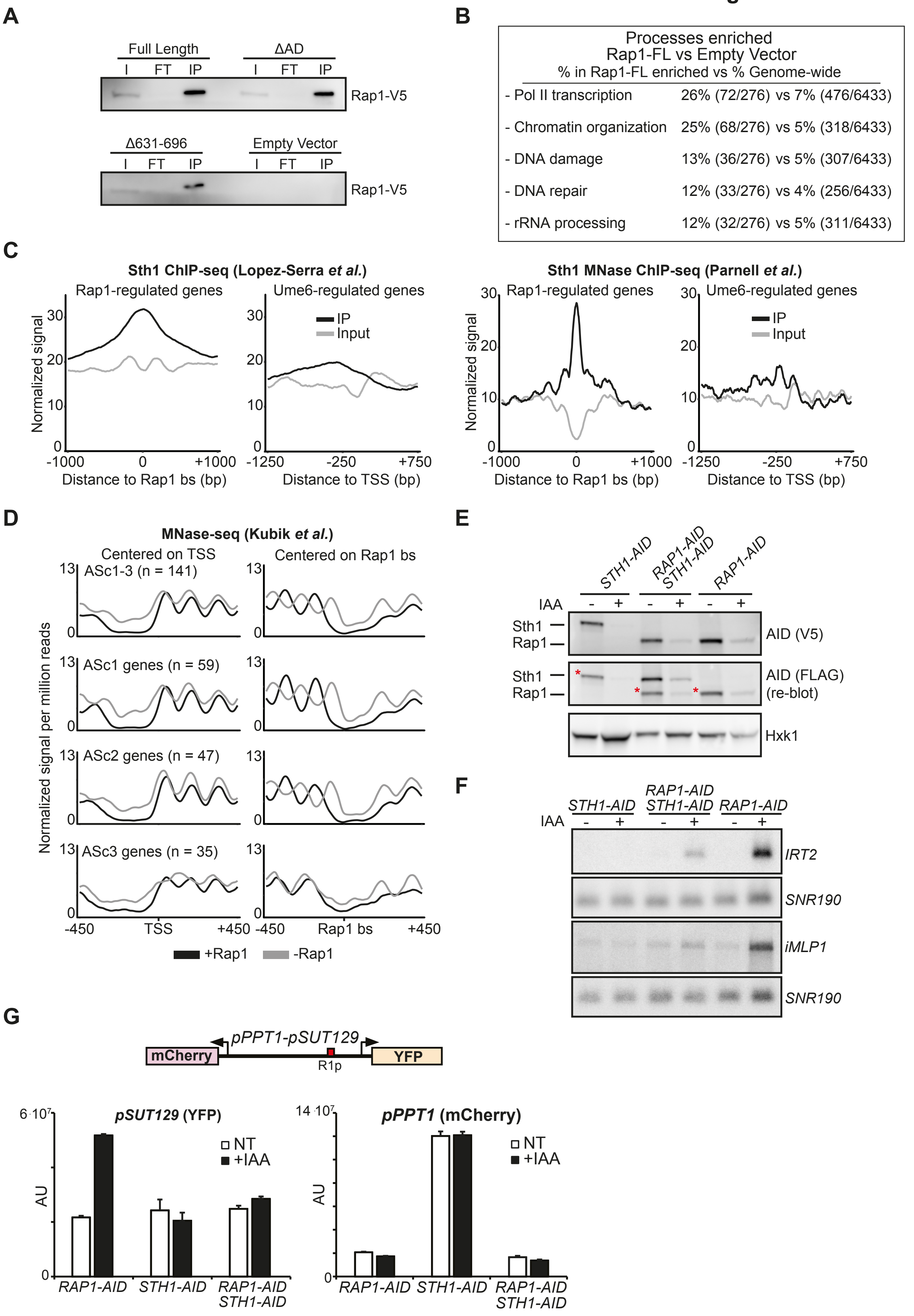

Figure S6. Co-depletion of RSC with Rap1 suppresses divergent transcription, Related to Figure 6.

(A) Expression of V5-tagged Rap1 domain mutant proteins detected after immunoprecipitation from MNase-treated chromatin extracts as described in Figure 6A. 0.67% of input (I), 0.67% of flow-through (FT), and 10% of immunoprecipitated sample (IP) eluted from anti-V5 beads was used for western blot. Rap1 expression was detected with anti-V5 antibody. Samples were collected from strains containing single copy integration vectors either expressing V5-tagged Rap1: FL (FW5420), ΔAD (FW5424), Δ631-696 (FW5396) or an empty vector (FW5399). **(B)** Expanded Yeast GO-Slim Process analysis of proteins enriched with chromatin bound Rap1, as described in Figure 6C. The GO-Slim terms and their frequencies of proteins enriched in Rap1-FL vs empty vector control and genome-wide are shown. 13 proteins with ambiguous gene assignment were excluded from the analysis. **(C)** Metagene plots of Sth1 ChIP-seq (left) and Sth1 MNase ChIP-seq (right) data at Rap1-regulated promoters. The normalized signal per million reads is plotted on the y-axis, 1 kb up- and downstream of the promoter Rap1 binding sites (n = 141). As control, we generated metagene plots for Ume6-regulated gene promoters (n = 87). Signals from immunoprecipitated (black) and input (gray) samples are shown. For Sth1 MNase ChIP-seq, chromatin was liberated by MNase digestion prior to immunoprecipitation. Data were obtained from GEO (GSE56994 & GSE65594) (Lopez-Serra et al., 2014; Parnell et al., 2015). **(D)** MNase-seq metagene plots showing that promoters with Rap1-dependent divergent transcription show differences in nucleosome occupancy. Normalized signal per million reads is shown 450 bp up- and downstream of the transcription start site (TSS, left panel), or Rap1 binding site (Rap1 bs, right panel). Separate plots are shown for the set of Rap1-regulated genes as shown in Figure 2F (n = 141), and each cluster of genes according to the antisense strand (ASc1, ASc2, ASc3). Nucleosome positions before (black) and after (gray) Rap1 depletion are shown for each plot. Data were obtained from GEO (GSE73337) (Kubik et al., 2015). **(E)** Auxin-induced depletion (AID) of Rap1 and Sth1 detected by western blotting. Samples were collected from *RAP1-AID* (FW3877), *STH1-AID* (FW6032), and *RAP1-AID STH1-AID* (FW6231) cells as in Figure 6F, before (-IAA) and after (+IAA) auxin treatment. Depletion of V5-AID tagged proteins was detected with an anti-V5 antibody. Sth1-AID-FLAG was detected with an anti-FLAG antibody after re-probing of the V5-blot. The asterisks on the FLAG blot indicate the residual V5 blot signal. Hxk1 was detected as a loading control. **(F)** *IRT2* and *iMLP1* expression in cells co-depleted for Sth1 and Rap1. Same data as described in Figure 6F, except that the original order of loading for the samples is displayed. **(G)** Reporter assay showing that co-depletion of RSC and Rap1 suppresses noncoding transcription. Quantification of *SUT129* (YFP) and *PPT1* (mCherry) promoter activity as described in Figure 6H. Cells harbouring *RAP1-AID* (FW6206), *STH1-AID* (FW6218), and *RAP1-AID STH1-AID* (FW6433) were either not treated (NT) or treated with IAA (+IAA). Mean signals corrected for background (AU, arbitrary units) are plotted. The error bars represent 95% confidence intervals (n = 50 cells).

Figure S7. Wu *et al.*

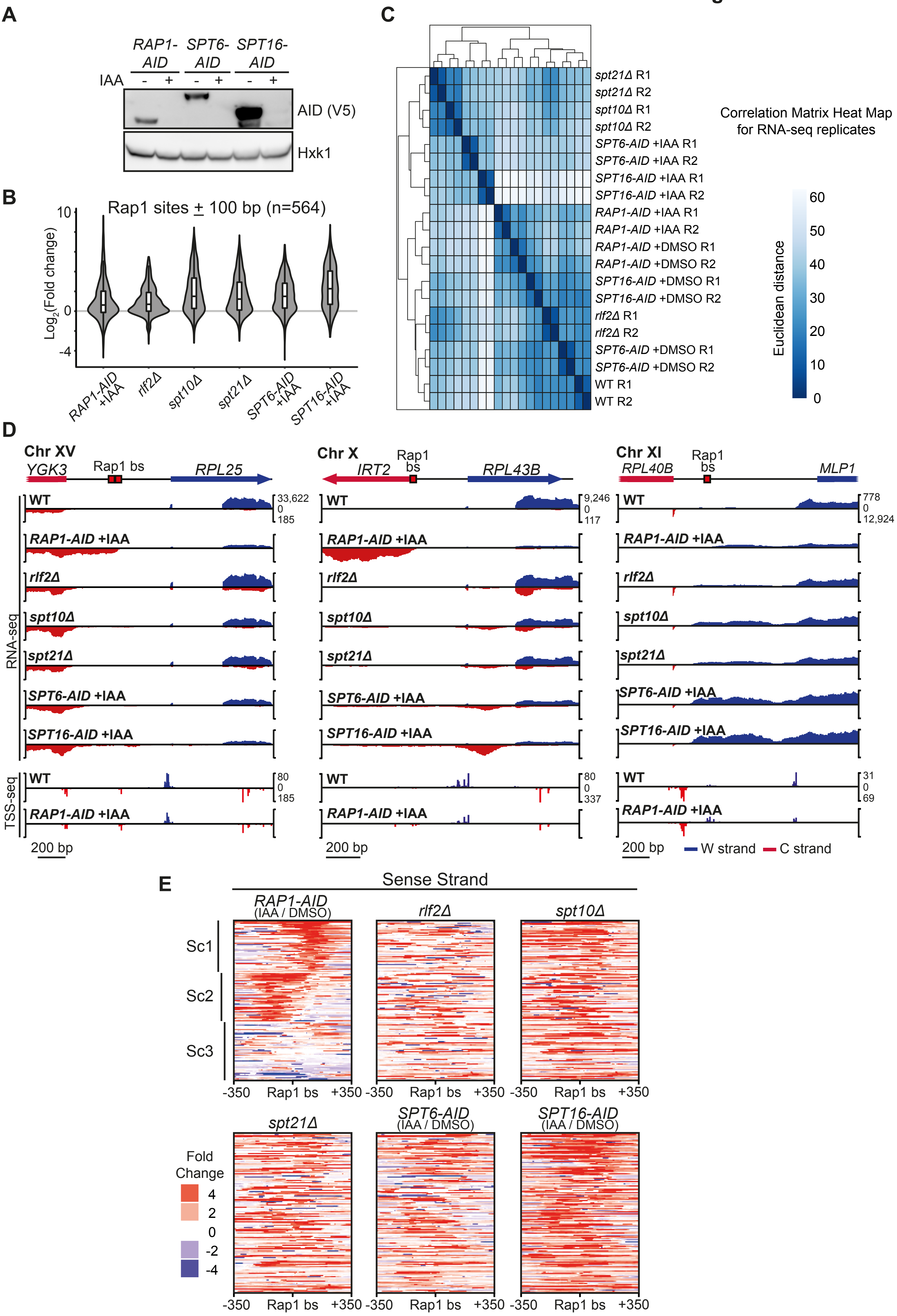

Figure S7. Rap1 suppresses divergent transcription independent of chromatin assembly and remodelling pathways, Related to Figure 7.
(A) Depletion of Rap1, Spt6, and Spt16 detected by western blotting. *RAP1-AID* (FW3877), *SPT6-AID* (FW5555), and *SPT16-AID* (FW5559) cells were either mock-treated with DMSO (-) or treated with IAA (+) as in Figure 7A. AID-tagged proteins were detected with an anti-V5 antibody. Hxk1 was used as a loading control. **(B)** Violin and box-and-whisker plots showing the distribution of changes in RNA expression around Rap1 sites. Fold change values of *rlf2Δ* (FW5609), *spt10Δ* (FW5543), and *spt21Δ* (FW5547) compared to a wild-type control (WT, FW629) are displayed, whereas for *RAP1-AID* (FW3877), *SPT6-AID* (FW5555), and *SPT16-AID* (FW5559) fold change values were obtained by comparing IAA treatment (+IAA) to mock treatment (+DMSO). The distribution of RNA expression changes for ±100 bp windows (n = 1128) around Rap1 binding sites (n = 564) is shown on the y-axis, calculated from two independent experiments. **(C)** Correlation matrix heat map for total RNA-seq biological replicate samples, showing the Euclidean distance between the samples across the genome (based on all genes). **(D)** Divergent transcription at the *RPL25, RPL43B*, and *RPL40B* loci, comparing Rap1-repressed noncoding transcription to chromatin regulators (*RLF2*, *SPT10*, *SPT21*, *SPT6*, and *SPT16*). RNA-seq data of strains described in A and B. The normalized reads are shown on the y-axis for the Watson (W, blue) and Crick (C, red) strands. Rap1 binding sites are shown as red boxes. The TSS-seq tracks for wild-type (WT) and Rap1 depletion (*RAP1-AID* +IAA) are displayed in the last two tracks. **(E)** Heat maps showing the changes in RNA expression as described for Figure 7C, except that data from sense strand was used for the analysis. A colour scale displays the fold change values.

Table S1. Summary of Rap1 silencing mutant screen for *IRT2* expression, Related to Figure 5

plasmid	mutant	*IRT2*	HM silencing	telomere silencing	telomere length
			(Feeser and Wolberger, PMID 18538788)		
372	EV	+++	NA	NA	NA
501	Δ672-827	+	loss	loss	increase
540	T700A D701A R747A K748A N749A Patch 2A	-	wt	wt	increase
543	D761A M763A M817A Patch 5A	-	wt	decrease	increase
544	D761A M763A R814A M817A Patch 5B	-	wt	decrease	increase
493	R747A	-	wt	wt	increase
495	M817A	-	wt	decrease	increase
497	R804A M817A	-	wt	decrease	increase
498	T700A D701A R747A K748A N749A S753A Patch 2B	-	wt	wt	increase
499	S725A D727A E729A Patch 4	-	loss	wt	increase
516	D727A	-	loss	wt	increase
531	M817R	-	wt	decrease	increase
539	N679A I682A N782A Patch 1	-	wt	wt	wt
541	H709A D742A E743A Patch 3A	-	wt	wt	increase
542	L706A H709A D742A E743A Patch 3B	-	wt	wt	increase
545	R747S	-	NA	NA	NA
492	H709A	-	wt	wt	increase
494	M763A	-	wt	decrease	decrease
496	M763A M817A	-	wt	decrease	wt
500	K796A R804A T812A Patch 6	-	wt	wt	increase
510	D689A	-	wt	wt	wt
511	K696A	-	wt	wt	wt
512	D701A	-	wt	wt	wt
513	D701R	-	wt	wt	wt
514	Q715A	-	wt	wt	wt
515	D701A H789A	-	wt	wt	wt
517	E729A	-	wt	wt	wt
518	S731A	-	wt	wt	wt
519	S731Y	-	wt	wt	wt
520	E734A	-	wt	wt	wt
521	E743R	-	wt	wt	wt
522	S753Y	-	wt	wt	wt
523	N782R	-	wt	wt	wt
524	H789A	-	wt	wt	wt
525	D790A	-	wt	wt	wt
526	K796A	-	wt	wt	increase
527	N798A	-	wt	wt	wt
528	Q800A	-	wt	wt	wt
529	E801A	-	wt	wt	wt
530	R804A	-	wt	wt	wt
532	M817Y	-	wt	wt	wt
533	R820A	-	wt	wt	wt
534	D701A R747A	-	wt	wt	increase
535	N798A D799A	-	wt	wt	wt
536	R804A T812A	-	wt	wt	wt
537	S731Y M763A	-	wt	decrease	wt
538	S731Y R820E	-	wt	wt	wt
471	FL control	-	NA	NA	NA

Table S3. Summary of screen for regulators of *IRT2* and *iMLP1* expression, Related to Figure 7

gene	mutant type	strain reference	*IRT2* levels	*iMLP1* levels	mutant type	*IRT2*	*iMLP1*
					(van Bakel *et al.*, PMID 23658529)		
Ada2	deletion	FW6715	-	-	NA	NA	NA
Arp8	deletion	FW6707	-	-	NA	NA	NA
Bre1	deletion	FW6722	-	-	deletion	-	-
Bur2	deletion	FW4817	-	-	NA	NA	NA
Cdc40	deletion	FW6683	-	+	NA	NA	NA
Cmr1	deletion	FW6725	-	-	NA	NA	NA
Ctk1	deletion	FW4756	-	-	NA	NA	NA
Est2	deletion	FW4757	-	-	NA	NA	NA
Gcn4	deletion	FW6682	-	+	NA	NA	NA
Gcn5	deletion	FW6717	-	-	NA	NA	NA
Gcr2	deletion	FW6698	-	-	NA	NA	NA
Hst1	deletion	FW6721	-	-	NA	NA	NA
Hst2	deletion	FW6691	-	-	NA	NA	NA
Hst3	deletion	FW6678	-	+	NA	NA	NA
Hst4	deletion	FW6686	-	-	NA	NA	NA
Ino80	deletion	FW4819	-	-	deletion	-	-
Isw1	deletion	FW6681	-	+	deletion	-	-
Isw2	deletion	FW6679	-	-	deletion	-	-
Mga2	deletion	FW6700	-	-	NA	NA	NA
Ngg1	deletion	FW6687	-	-	NA	NA	NA
NHP6A	deletion	FW6719	-	-	NA	NA	NA
Nrd1	AID	FW4821	-	-	NA	NA	NA
Opi3	deletion	FW6701	-	-	NA	NA	NA
Paf1	deletion	FW6706	-	-	deletion	-	+
Rap1	AID	FW3877	++	++	ts and tet-off	++	++
Rif1	deletion	FW6729	-	+	NA	NA	NA
Rif2	deletion	FW6704	-	-	NA	NA	NA
Rlf2	deletion	FW6703	-	++	deletion	-	+
Rpb9	deletion	FW6694	-	+	NA	NA	NA
Rpd3	deletion	FW6689	-	-	deletion	-	-
Rrd1	deletion	FW6708	-	-	NA	NA	NA
Rrp6	deletion	FW6680	-	-	NA	NA	NA
Rsc1	deletion	FW6685	-	+	NA	NA	NA
Rsc2	deletion	FW6718	-	-	NA	NA	NA
Rtt106	deletion	FW6699	-	+	NA	NA	NA
Rtt109	deletion	FW6677	-	+	NA	NA	NA
Sch9	deletion	FW4820	-	-	NA	NA	NA
Set2	deletion	FW6728	-	-	deletion	-	-
Set3	deletion	FW6709	-	-	NA	NA	NA
Sgf29	deletion	FW6726	-	-	NA	NA	NA
Sgf73	deletion	FW6695	-	-	NA	NA	NA
Sin4	deletion	FW6716	-	-	NA	NA	NA
Sir1	deletion	FW6705	-	-	NA	NA	NA
Sir2	deletion	FW6711	-	-	deletion	-	-
Sir3	deletion	FW6713	-	-	NA	NA	NA

Table S3. Summary of screen for regulators of *IRT2* and *iMLP1* expression, Related to Figure 7

gene	mutant type	strain reference	*IRT2* levels	*iMLP1* levels	mutant type	*IRT2*	*iMLP1*
					(van Bakel *et al.*, PMID 23658529)		
Sir4	deletion	FW6690	-	-	NA	NA	NA
Snf2	deletion	FW6724	-	-	deletion	-	-
Snf5	deletion	FW6723	-	-	NA	NA	NA
Spt10	deletion	FW5543	-	++	deletion	-	++
Spt16	AID	FW5559	+	++	NA	+	++
Spt21	deletion	FW6676	-	++	deletion	-	++
Spt23	deletion	FW4758	-	-	NA	NA	NA
Spt3	deletion	FW6684	-	-	NA	NA	NA
Spt4	deletion	FW6710	-	-	NA	NA	NA
Spt6	AID	FW5555	-	+	ts	+	++
Spt7	deletion	FW6714	-	-	NA	NA	NA
Spt8	deletion	FW6675	-	-	NA	NA	NA
Srb2	deletion	FW6702	-	-	NA	NA	NA
Ssn3	deletion	FW6693	-	-	NA	NA	NA
Sum1	deletion	FW6696	-	-	NA	NA	NA
Swi3	deletion	FW6688	-	-	NA	NA	NA
Swr1	deletion	FW6697	-	-	deletion	-	-
Trf4	deletion	FW6720	-	-	NA	NA	NA
Ubp3	deletion	FW6712	-	-	NA	NA	NA
Vps16	deletion	FW6692	-	-	NA	NA	NA
Xrn1	deletion	FW4759	-	-	NA	NA	NA

Table S5. Plasmids, Related to STAR Methods

plasmid number	plasmid name
255	*pFA6A-V5::KanMX6*
252	*pFA6A-V5-IAA7::KanMX6*
547	*pKAN-IAA17 (71-114)-Myc::KanMX*
546	*pHYG-IAA17 (71-114)-FLAG::hphNT*
250	*pNH603 pGPD1-osTIR1 HIS3*
247	*pNH605 pGPD1-osTIR1 LEU2*
227	*NatMX gene deletion*
471	*pNH603 Myc-NLS-Rap1 (1-827)::HIS3*
472	*pNH603 Myc-NLS-Rap1 (339-827)::HIS3*
473	*pNH603 Myc-NLS-Rap1 (1-599)::HIS3*
474	*pNH603 Myc-NLS-Rap1 (339-599)::HIS3*
372	*pNH603::HIS3 single copy integration vector*
477	*pNH603 HA-NLS-Rap1 (1-827)::HIS3*
478	*pNH603 HA-NLS-Rap1 (ΔDBD 362-597)::HIS3*
479	*pNH603 HA-NLS-Rap1 (ΔTox 597-662)::HIS3*
480	*pNH603 HA-NLS-Rap1 (ΔAD 631-678)::HIS3*
482	*pNH603 HA-NLS-Rap1 (Δ764-827)::HIS3*
483	*pNH603 HA-NLS-Rap1 (Δ631-696)::HIS3*
566	*pNH603 HA-NLS-Rap1 (1-827)-V5::HIS3*
558	*pNH603 HA-NLS-Rap1 (ΔDBD 362-597)-V5::HIS3*
559	*pNH603 HA-NLS-Rap1 (ΔTox 597-662)-V5::HIS3*
568	*pNH603 HA-NLS-Rap1 (ΔAD 631-678)-V5::HIS3*
561	*pNH603 HA-NLS-Rap1 (Δ764-827)-V5::HIS3*
562	*pNH603 HA-NLS-Rap1 (Δ631-696)-V5::HIS3*
105	*LoxP-KanMX5-LoxP*
106	*LoxP-HIS5MX4-LoxP*
108	*LoxP-KlURA3MX4-LoxP*
109	*pGAL1-CRE::URA3*
110	*pGAL1-CRE::HIS3*
592	*YFP-pPPT1-mCherry::NatMX6*
593	*YFP-R1p(SspI)-pPPT1-mCherry::NatMX6*
595	*YFP-R1prv(SspI)-pPPT1-mCherry::NatMX6*
618	*YFP-R1d(XmnI)-pPPT1-mCherry::NatMX6*
492-501, 510-545	*pNH603 Myc-NLS-Rap1 (point or patch mutant)::HIS3, see Table S1*

Table S6. Oligonucleotides, Related to STAR Methods

Primer no	Sequence (5' - 3')	Name	Notes
N/A	CACTCTrGrArGrCrArArUrArCrC	TSS-seq RNA adapter	5' RNA adapter oligonucleotide ligated to 5' end of decapped RNA fragments - TSS sequencing protocol.<br>5' RNA adapter ligated as RNA:DNA hybrid to 5' ends of decapped RNA fragments.
N/A	GCAC[iBiodT]GCACTCTGAGCAATACC	TSS-seq 2nd strand synthesis primer	Primer for 2nd strand synthesis in TSS sequencing protocol (internally biotinylated)
489	ATGCAACGCCTACTTGTTTT	IME1 -2400 REV	Oligos to amplify *IRT2* northern blot probe DNA template from genomic DNA
493	GATGGAGGGTTGGCATAAAA	IME1 UME6Δ check FW	Oligos to amplify *IRT2* northern blot probe DNA template from genomic DNA
1130	TGCACCCAGACAACTACACA	AW10_RPL40B_ncRNA_probef	Oligos to amplify *iMLP1* northern blot probe DNA template from genomic DNA
1131	CGCCGTAAGACTCAATGGAC	AW11_RPL40B_ncRNA_prober	Oligos to amplify *iMLP1* northern blot probe DNA template from genomic DNA
2111	GGCCCTGATGATAATG	AW411_SNR190_NBprobe_fwd	Oligos to amplify *SNR190* northern blot probe DNA template from genomic DNA
2112	GGCTCAGATCTGCATG	AW412_SNR190_NBprobe_rev	Oligos to amplify *SNR190* northern blot probe DNA template from genomic DNA
1701	TGCGGCTGGTATGGTATTGTAAGG	AW255_pRPL43B_Rap1_ChIP_A_fwd	Oligos to amplify region adjacent to Rap1 binding sites at *pRPL43B* , for ChIP-qPCR
1702	AAAGGCAGAAGATGGGCGGC	AW256_pRPL43B_Rap1_ChIP_B_rev	Oligos to amplify region adjacent to Rap1 binding sites at *pRPL43B* , for ChIP-qPCR
2170	GCTTTACCTCTTGCTGAACGGGA	AW434_pRPL40B_ChIP_A_fwd	Oligos to amplify region adjacent to Rap1 binding sites at *pRPL40B* , for ChIP-qPCR
2171	TCCGCCATATGATCCGCCTC	AW435_pRPL40B_ChIP_A_rev	Oligos to amplify region adjacent to Rap1 binding sites at *pRPL40B* , for ChIP-qPCR
106	GTACCACCATGTTCCCAGGTATT	FvW_ACTFrt	Oligos to ampify region at 3' end of *ACT1* ORF, for ChIP-qPCR
268	AGATGGACCACTTTCGTCGT	FvW_ACT1rt	Oligos to ampify region at 3' end of *ACT1* ORF, for ChIP-qPCR
N/A	TATGTATGGGTTAAAAAGGATGTATGGATG	FW-ppt_rap1-3top2	Oligos to clone transcription factor binding sites into fluorescent reporter plasmid (blunt-end cloning)
N/A	CATCCATACATCCTTTTTAACCCATACATA	FW-ppt_rap1-3bottom2	Oligos to clone transcription factor binding sites into fluorescent reporter plasmid (blunt-end cloning)
