# Supplementary material for: Repression of Divergent Noncoding Transcription by a Sequence-Specific Transcription Factor
Source: Mol Cell. 2018 Dec 20;72(6):942–954.e7. doi: 10.1016/j.molcel.2018.10.018 (PMC6310685; doi:10.1016/j.molcel.2018.10.018)
Supplement: Document S2. Article plus Supplemental Information [file mmc4.pdf]

Molecular Cell

Article

Repression of Divergent Noncoding Transcription by a Sequence-Specific Transcription Factor

Graphical Abstract

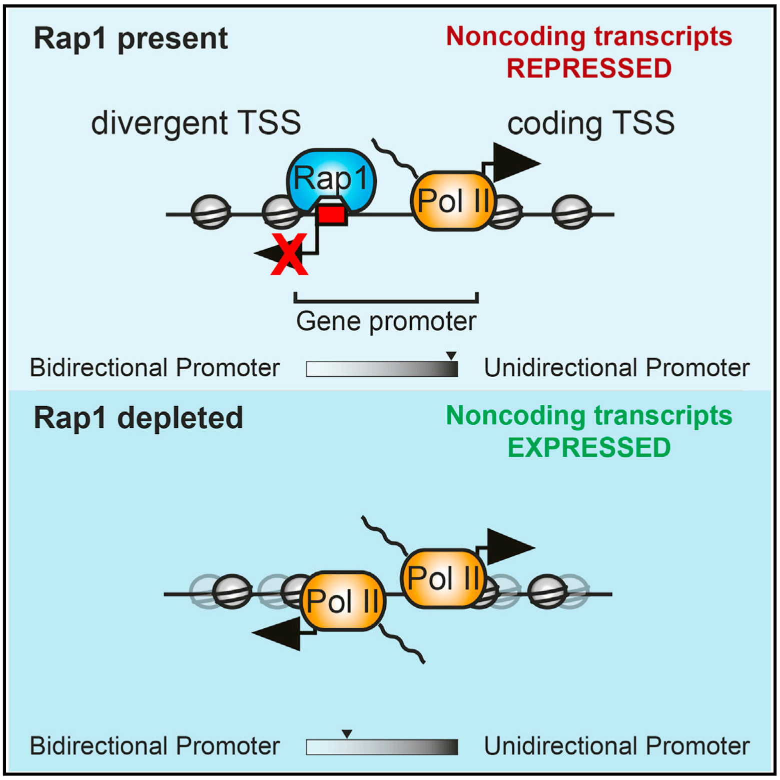

Highlights

- Expression of divergent noncoding RNAs is repressed by Rap1
- Rap1 prevents initiation of divergent noncoding transcription near its binding sites
- Rap1 provides directionality toward productive transcription

Authors

Andrew C.K. Wu, Harshil Patel, Minghao Chia, Fabien Moretto, David Frith, Ambrosius P. Snijders, Folkert J. van Werven

Correspondence

folkert.vanwerven@crick.ac.uk

In Brief

Wu et al. characterize how divergent noncoding RNAs are repressed at highly expressed gene promoters in yeast. The authors identified that the sequence-specific transcription factor Rap1 restricts transcription initiation in the divergent direction locally near its binding site, thereby controlling promoter directionality.

Wu et al., 2018, Molecular Cell *72*, 942–954
December 20, 2018 © 2018 The Author(s). Published by Elsevier Inc.
https://doi.org/10.1016/j.molcel.2018.10.018

CellPress

CellPress

Molecular Cell
Article

Repression of Divergent Noncoding Transcription by a Sequence-Specific Transcription Factor

Andrew C.K. Wu,[1] Harshil Patel,[2] Minghao Chia,[1] Fabien Moretto,[1] David Frith,[3] Ambrosius P. Snijders,[3] and Folkert J. van Werven[1,4,*]
[1]Cell Fate and Gene Regulation Laboratory, The Francis Crick Institute, 1 Midland Road, London NW1 1AT, UK
[2]Bioinformatics and Biostatistics, The Francis Crick Institute, 1 Midland Road, London NW1 1AT, UK
[3]Protein Analysis and Proteomics Platform, The Francis Crick Institute, 1 Midland Road, London NW1 1AT, UK
[4]Lead Contact
*Correspondence: folkert.vanwerven@crick.ac.uk
https://doi.org/10.1016/j.molcel.2018.10.018

SUMMARY

Many active eukaryotic gene promoters exhibit divergent noncoding transcription, but the mechanisms restricting expression of these transcripts are not well understood. Here, we demonstrate how a sequence-specific transcription factor represses divergent noncoding transcription at highly expressed genes in yeast. We find that depletion of the transcription factor Rap1 induces noncoding transcription in a large fraction of Rap1-regulated gene promoters. Specifically, Rap1 prevents transcription initiation at cryptic promoters near its binding sites, which is uncoupled from transcription regulation in the protein-coding direction. We further provide evidence that Rap1 acts independently of previously described chromatin-based mechanisms to repress cryptic or divergent transcription. Finally, we show that divergent transcription in the absence of Rap1 is elicited by the RSC chromatin remodeler. We propose that a sequence-specific transcription factor limits access of basal transcription machinery to regulatory elements and adjacent sequences that act as divergent cryptic promoters, thereby providing directionality toward productive transcription.

INTRODUCTION

Precise control of gene expression is critical for all cellular functions. How and when genomes produce coding messenger RNAs and prevent the expression of unwanted RNAs has been a long-standing question of interest. In this context, an apparent paradox exists: genomic locations of coding gene transcription also produce aberrant noncoding transcripts. The transcriptionally active coding gene promoters, which often express noncoding transcripts in the antisense direction (Neil et al., 2009; Seila et al., 2008; Xu et al., 2009), are a major source. This process is known as divergent or bidirectional transcription. The functions of the noncoding RNAs produced and the mechanisms that limit expression of divergent noncoding transcripts are not well understood.

Divergent noncoding transcription is present across eukaryotic species. A large fraction of all noncoding transcripts emanate from divergent or bidirectional gene promoters (Neil et al., 2009; Seila et al., 2008; Xu et al., 2009). Typically, divergent noncoding transcripts initiate within or nearby coding gene promoters, but they do not share the same core promoter as transcripts in the coding direction (Andersson et al., 2015; Duttke et al., 2015; Rhee and Pugh, 2012; Scruggs et al., 2015). The transcription of divergent noncoding RNAs is lower than coding genes (Churchman and Weissman, 2011). Divergent noncoding transcripts are unstable and rapidly degraded. The Nrd1-Nab3-Sen1 and premature polyadenylation signal pathways in yeast and mammalian cells, respectively, terminate and degrade divergent transcripts (Jensen et al., 2013). In addition, exosome and nonsense-mediated decay pathways degrade cryptic and divergent transcripts (Neil et al., 2009; van Dijk et al., 2011). Divergent and pervasive transcription can also be repressed by controlling TATA-binding protein activity (Xue et al., 2017). Finally, CAF-1-mediated chromatin assembly represses the accumulation of divergent noncoding transcripts at promoters, which in turn is opposed by chromatin regulators that promote rapid turnover of nucleosomes (Marquardt et al., 2014).

In budding yeast, 138 genes encode for the protein subunits of the ribosome. These highly expressed ribosomal protein (RP) genes account for approximately half of all RNA polymerase II transcription (Warner, 1999). Transcription of nearly all RP genes is controlled by the pioneer transcription factor Rap1, which binds to upstream elements in RP gene promoters (Lieb et al., 2001). When RP promoters are active, Rap1 recruits coactivators such as Fhl1, Ifh1, and Sfp1, as well as basal transcription factors like TFIID and TFIIA (Azad and Tomar, 2016; Hu and Li, 2007). Thus, Rap1 orchestrates RP gene expression. Given that RP genes are among the most actively transcribed genes in yeast, they are an ideal model for studying how aberrant transcription is controlled.

Here, we describe how divergent noncoding transcription is repressed at highly active RP gene promoters. We find that depletion of Rap1, but not other transcription factors important for RP expression, causes transcription in the divergent direction. Rap1 represses noncoding transcription typically within 50 bp of the Rap1 motif, which is uncoupled from transcription

 © 2018 The Author(s). Published by Elsevier Inc.
This is an open access article under the CC BY license (http://creativecommons.org/licenses/by/4.0/).

regulation in the protein-coding direction. We further show that Rap1-mediated repression of divergent transcription is distinct from known chromatin-based mechanisms. Thus, a sequence-specific transcription factor controls promoter directionality by repressing transcription in the divergent direction. Our work adds a new layer of regulation to various mechanisms that limit expression of aberrant transcripts and defines how promoter directionality is controlled.

RESULTS

Depletion of Rap1 Causes Divergent Transcription at *RPL43B* and *RPL40B*

In budding yeast, a large fraction of bidirectional promoters express noncoding transcripts, also known as cryptic unstable transcripts (CUTs) or stable unannotated transcripts (SUTs), in the divergent direction (Neil et al., 2009; Xu et al., 2009). Transcription of divergent CUTs and SUTs typically correlates with nucleosome-depleted regions (NDRs) and promoter activity in the coding gene direction. Considering that RP genes are among the most highly expressed genes in yeast, surprisingly few RP gene promoters (16 out of 138 promoters) display an annotated divergent noncoding transcript (CUT or SUT) (Neil et al., 2009; Xu et al., 2009). We hypothesized that RP promoters must have a robust mechanism for limiting divergent noncoding transcription.

To investigate this, we deleted or depleted transcription factors important for RP gene regulation. We selected the *RPL43B* and *RPL40B* genes to study, since both promoters are directly adjacent to a divergent noncoding transcript: *IRT2* and *SUT242*, respectively (Figure 1A). Four RP gene transcription factors (Fhl1, Ifh1, Sfp1, and Rap1) have essential roles in cellular fitness (Hu and Li, 2007); hence, we generated auxin-inducible degron (AID) alleles (Nishimura et al., 2009) (Figure 1B). We measured the expression of divergent transcripts by northern blot using probes directed against *IRT2* and *SUT242*. No effects on *IRT2* and *SUT242* expression were observed when we depleted Fhl1, Ifh1, or Sfp1, or in *hmo1*Δ or *crf1*Δ cells (Figure 1C). Strikingly, Rap1-depleted cells (*RAP1-AID* + 3-indoleacetic acid [IAA]) showed strong induction of *IRT2* (Figure 1C). In addition, the *RPL40B* promoter displayed expression of multiple divergent transcripts upon Rap1 depletion. The transcript with the strongest signal approximated the size of the adjacent *MLP1* gene, which we define as isoform of *MLP1* (*iMLP1*). *IRT2* and *iMLP1* expression increased simultaneously as Rap1 protein levels decreased (Figures 1D and S1A). Thus, Rap1 specifically represses divergent transcription at the *RPL43B* and *RPL40B* promoters.

Rap1 is a pioneer transcription factor that binds to DNA sequence elements in RP and metabolic gene promoters (Lieb et al., 2001). To examine whether the Rap1 binding site (bs) is important for repressing divergent noncoding transcription, we deleted Rap1 motifs in the *RPL43B* and *RPL40B* promoters (*RPL43B-bs*Δ and *RPL40B-bs*Δ). *IRT2* and *iMLP1* expression levels increased in *RPL43B-bs*Δ and *RPL40B-bs*Δ, respectively, to a level comparable to Rap1-depleted cells (*RAP1-AID* + IAA) (Figures 1E, 1F, and S1B). Initiation of *IRT2* transcription occurred downstream of the Rap1 bs in *RPL43B-bs*Δ, because the *IRT2* transcript length increased due to the residual loxP sequence. Thus, Rap1 binding is required to repress divergent noncoding transcription from the *RPL43B* and *RPL40B* promoters.

Transcription within intergenic regions affects local coding gene expression (Ard et al., 2017). We examined the effect of divergent transcription on the expression of neighboring genes. Previous work showed that *IRT2* is part of a regulatory circuit that facilitates expression of *IME1*, the master regulator of entry into meiosis (Moretto et al., 2018). We hypothesized that Rap1 prevents mis-expression of *IRT2* from affecting *IME1* levels. Indeed, median *IME1* expression increased from 5 transcripts per cell in wild-type (WT) to 16 in the *RPL43B-bs*Δ mutant (Figures 1G and S1C). We also investigated the effect of *iMLP1* expression. *iMLP1* is a long transcript isoform of *MLP1*; when we deleted *MLP1* in the *RPL40B-bs*Δ cells, *iMLP1* disappeared and a shorter transcript appeared (Figure 1F). Mlp1 protein levels were markedly reduced in *RPL40B-bs*Δ, suggesting that *iMLP1* transcription affects expression of *MLP1* (Figures 1H and S1D). The 5′ extended sequence of *iMLP1* harbors 15 upstream AUG sequences, which may render *iMLP1* translationally inert similar to other 5′ extended transcript isoforms (Chen et al., 2017; Cheng et al., 2018; Chia et al., 2017). We conclude that mis-regulation of Rap1-repressed divergent transcripts affects neighboring gene expression.

Rap1 Represses Noncoding Transcription near Its Binding Site

We next investigated how depleting Rap1 affects noncoding transcription at a genome-wide scale by RNA sequencing (RNA-seq). We performed RNA-seq on both polyadenylated (poly(A)) and total RNA (Figure S2A). As expected, the expression of Rap1-regulated coding genes decreased upon Rap1 depletion (Figures S2B and S2C) (Knight et al., 2014; Lieb et al., 2001). In addition, *IRT2* and *iMLP1* expression increased in IAA-treated *RAP1-AID* cells, whereas the control (DMSO) did not show *IRT2* or *iMLP1* expression (Figures 2A and S2D). We also observed noncoding transcription from other RP gene promoters. For example, the *RPL8A* promoter expressed a divergent transcript spanning the neighboring *GUT1* gene, but antisense to the coding sequence (Figure 2A). Consequently, sense *GUT1* expression was reduced 1.7-fold. Thus, RNA-seq is able to identify novel Rap1-repressed divergent transcripts.

Our data from example loci indicate that Rap1 mediates repression of noncoding transcription close to the Rap1 binding sites. To systematically determine how Rap1 depletion affects noncoding transcription, we binned RNA-seq data in windows of 50, 100, 200, and 500 bp up- and downstream of 564 annotated Rap1 sites (Figure 2B) (Lieb et al., 2001; Rhee and Pugh, 2011). For smaller windows (50 and 100 bp), approximately 40% of Rap1 binding sites displayed increased RNA expression (>2-fold) upon Rap1 depletion (Figure 2C). For the larger windows (200 and 500 bp), the number of Rap1 sites showing increased RNA expression decreased to 30% and 16%, respectively, suggesting a spatial effect limited to regions harboring Rap1 binding sites. Our analyses with different window sizes showed little difference between RNA-seq data from poly(A) RNA and total RNA (Figures 2D and S2E). In conclusion, Rap1

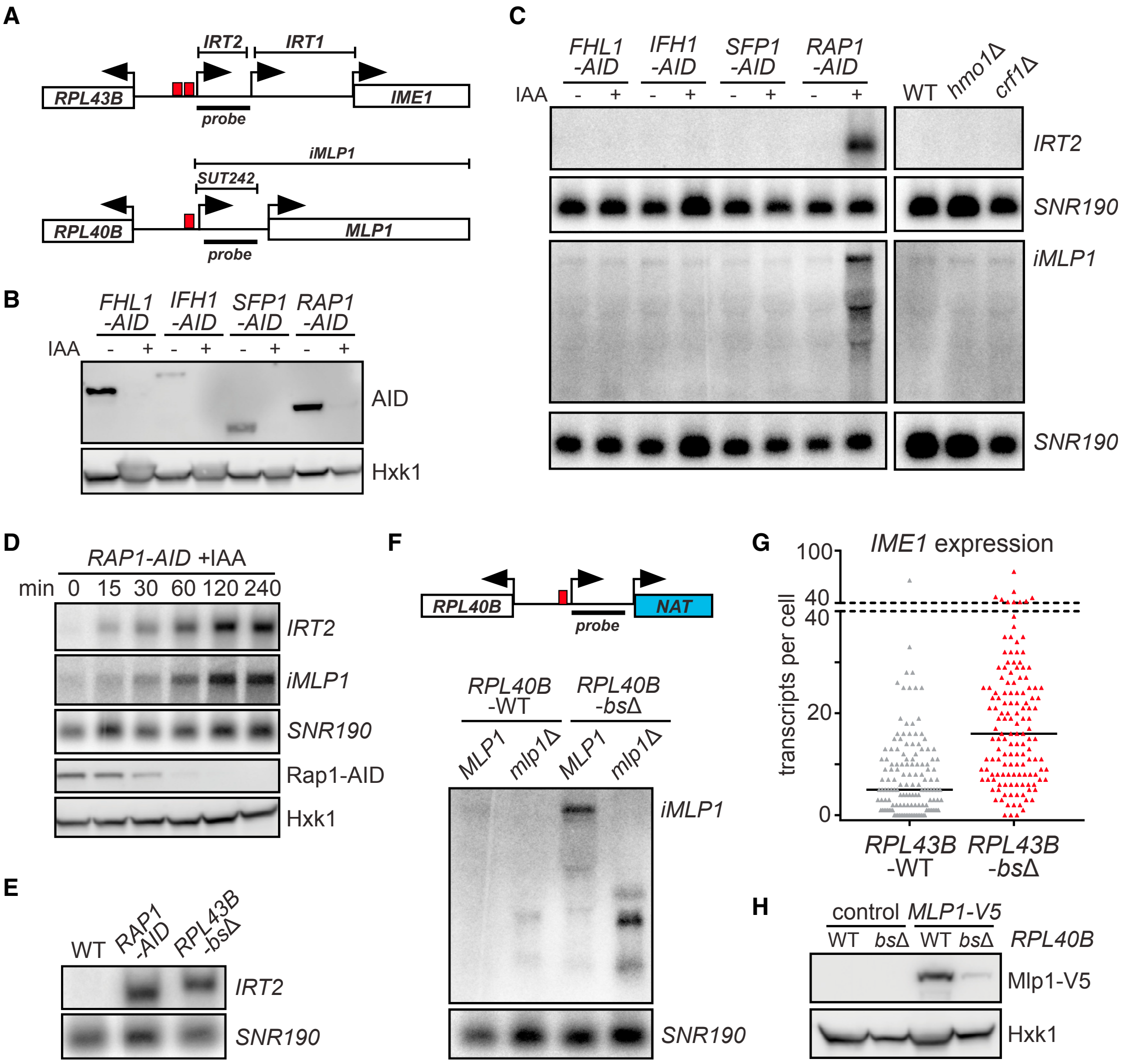

Figure 1. Rap1 Prevents Expression of Noncoding RNAs

(A) Schematic of two divergent ribosomal protein (RP) gene promoters. Red boxes depict Rap1 motifs.

(B) Auxin-induced depletion (AID) of transcription factors important for RP gene regulation detected by western blot. *FHL1-AID* (FW4200), *IFH1-AID* (FW4202), *SFP1-AID* (FW4204), and *RAP1-AID* (FW3877) cells were treated with 3-indole-acetic acid (IAA). Hxk1 was used as a loading control.

(C) *IRT2* and *iMLP1* expression detected by northern blot in cells described in (B), WT (FW629), *hmo1*Δ (FW4132), and *crf1*Δ (FW4136). ^{32}P-labeled probes targeting *IRT2* or *SUT242*/*iMLP1* and *SNR190* were used.

(D) Similar to (C), *RAP1-AID* (FW3877) with multiple time points (+ IAA).

(E) *IRT2* expression in *RPL43B-bs⊿* cells (FW3443). WT (FW629) and Rap1-depleted (FW3877) cells were included as in (C).

(F) *iMLP1* expression in *RPL40B-bs*Δ (FW4141), *mlp1*Δ (FW6030), and *mlp1*Δ *RPL40B-bs*Δ (FW6029) cells. *NAT*, nourseothricin marker; replaced *MLP1*.

(G) *IME1* expression in single diploid cells: WT (FW631) or *RPL43B-bs*Δ (FW6139). Each triangle represents transcript count for one cell and black lines indicate median number of transcripts per cell. n = 139 cells; *p < 0.0001 (unpaired Student's t test).

(H) Mlp1-V5 expression in WT (FW629), *RPL40B-bs⊿* (FW4141), *MLP1-V5* (FW4122), and *MLP1-V5 RPL40B-bs*Δ (FW4120) cells.

See also Figure S1.

represses transcription near Rap1 binding sites across the genome.

Next, we analyzed the RNA-seq data to identify features of cryptic transcript repression by Rap1. First, we determined whether there is a bias for the orientation of Rap1-repressed transcripts. We selected 141 Rap1 binding sites at well-annotated gene promoters regulated by Rap1 for further analysis (mostly RP genes) (Knight et al., 2014; Lieb et al., 2001). Expression near the Rap1 binding sites was upregulated in both the sense and antisense direction after Rap1 depletion; however, the largest increase was detected in the antisense direction (Figure 2E). A control set of promoters regulated by the repressor Ume6 was not affected by Rap1 depletion (McKnight et al., 2016). Second, we clustered the data centered on the Rap1 binding sites (Figures 2F and S2F–S2H). The antisense clusters 1 and 2 (ASc1 and ASc2) both displayed increased expression

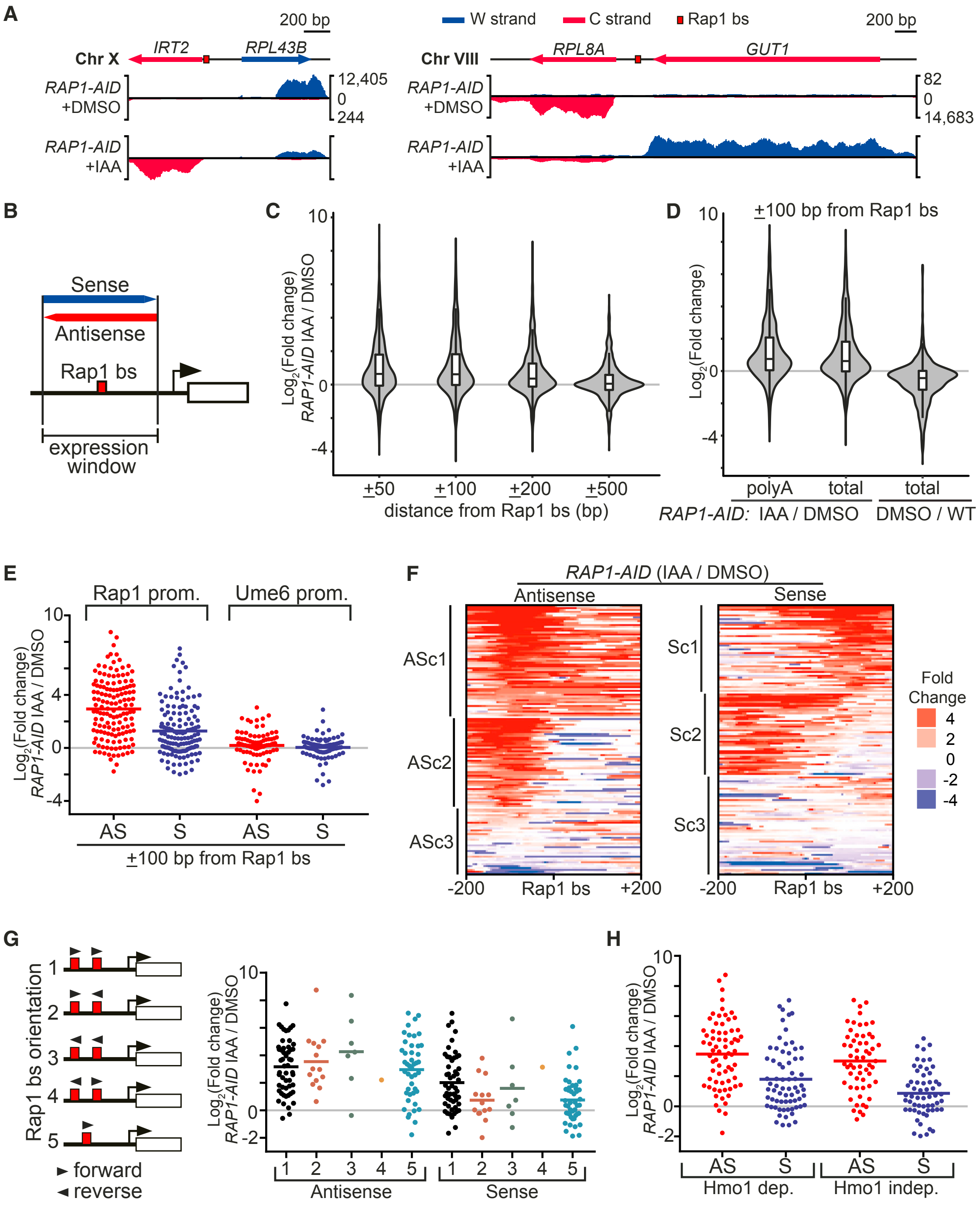

Figure 2. Rap1 Represses Divergent Noncoding Transcription

(A) Examples of divergent noncoding RNAs repressed by Rap1. IAA- and DMSO-treated *RAP1-AID* (FW3877) cells were processed for total RNA-seq. Normalized reads (y axis) for the Watson (W, blue) and Crick (C, red) strands.

(B) Scheme for determining RNA-seq signals around Rap1 binding sites. Reads that overlapped with the selected genomic region were counted.

(C) Violin and box-and-whisker plots of total RNA-seq data as in (A), showing expression changes for different window sizes. n = 564 Rap1 binding sites, signals for W and C strands were computed separately.

(D) Similar to C, comparing polyadenylated (poly(A)) and total RNA-seq data. As a control, the expression changes in *RAP1-AID* + DMSO over WT (FW629) are displayed (total RNA-seq).

(E) Similar to (D), using scatterplots to display expression changes for antisense (AS) and sense (S) strand windows relative to the coding gene for Rap1 (n = 141) and Ume6 (n = 87) -regulated promoters. Horizontal red or blue lines: mean value.

(legend continued on next page)

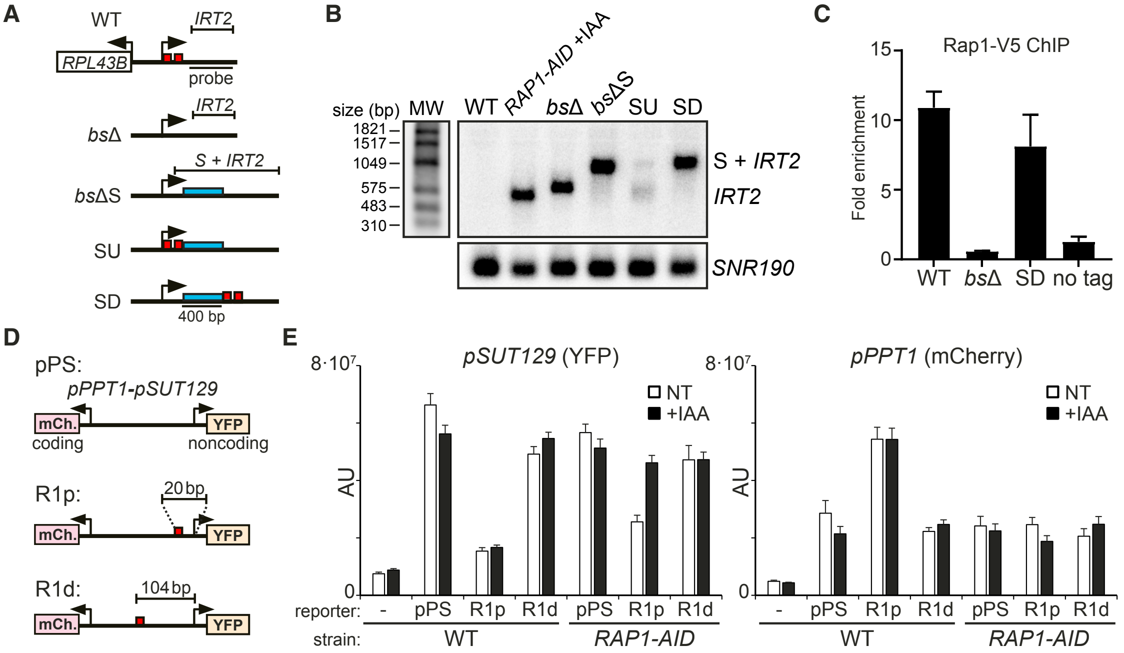

Figure 3. A Proximal Rap1 Motif Is Required and Sufficient to Repress Divergent Transcription

(A) Schematic of mutants (*bs*Δ, FW3440; *bs*ΔS, FW3920; SU, FW7451; SD, FW3922). Blue bar, spacer sequence; red boxes, Rap1 binding sites.

(B) *IRT2* expression in mutants described in A, WT (FW629), and *RAP1-AID* + IAA (FW3877). Northern blot membranes were probed for *IRT2* and *SNR190*. MW, RNA molecular weight marker.

(C) Rap1 binding to *RPL43B* promoter measured by chromatin immunoprecipitation (ChIP) (FW4732, FW4734, FW4735, and FW629). Data were normalized over *ACT1* and plotted as mean ± SEM (n = 3).

(D) Schematic of fluorescent reporter constructs. pPS was described previously (Marquardt et al., 2014). The Rap1 sites from the *RPL43B* promoter were integrated at a proximal (R1p, 20 bp) or distal (R1d, 104 bp) position to the TSS of *SUT129*.

(E) Ectopic repression of divergent noncoding transcription by Rap1. WT (FW629), pPS, R1p, and R1d in WT or *RAP1-AID* (FW6407; FW6895; FW7253; FW6208; FW6206; FW6408) cells were not treated (NT) or treated (+IAA), fixed, and imaged. Mean signals corrected for background (AU, arbitrary units) were plotted + 95% confidence intervals (n = 50 cells per sample).

See also Figure S3.

upstream of the Rap1 binding sites, while ASc3 showed very mild increase (Figure 2F). When we clustered for the sense direction signals, we observed that transcripts were upregulated both up- and downstream of the Rap1 site (Sc1 and Sc2). Finally, we examined whether the transcripts induced upon Rap1 depletion were enriched for specific classes of RP gene promoters (Knight et al., 2014). We found that the orientation or number of Rap1 motifs had little effect (Figure 2G). In addition, promoters regulated by Hmo1 displayed a comparable increase in expression to Hmo1-independent promoters (Figure 2H). Thus, Rap1 represses transcription near the Rap1 binding sites in the antisense, and to lesser extent, the sense direction.

A Proximal Rap1 Motif Is Required and Sufficient to Repress Divergent Transcription

Our results demonstrate that Rap1 represses noncoding transcription near the Rap1 binding sites. If close proximity of the Rap1 binding site to the cryptic promoter sequence is important, then increasing the distance should impair repression of noncoding transcription. To test this, we integrated 400-bp spacer sequences upstream or downstream of the Rap1 motifs relative to the *RPL43B* promoter (Figures 3A and 3B). When we integrated a spacer to replace Rap1 binding sites (*bs*ΔS), *IRT2* was expressed and the size of the transcript approximated the spacer sequence plus *IRT2* (S + *IRT2*) indicating that initiation of *IRT2* occurs downstream of the Rap1 binding sites. Strikingly, we observed a similar pattern when we integrated the spacer directly downstream (SD), but not upstream (SU), of the Rap1 binding sites relative to *RPL43B*. The spacer sequence had no effect on Rap1 binding to the *RPL43B* promoter (Figure 3C). Thus, the Rap1 binding sites must be nearby the cryptic promoter sequence for repression of divergent transcription.

Next, we determined whether the Rap1 binding site is sufficient to repress divergent transcription. We integrated Rap1

(F) Heatmaps showing changes in RNA expression on AS or S strands for data described in (E). Promoters were clustered on AS (ASc1–3) or S (Sc1–3) using k-means clustering (k = 3).

(G) Different classes of RP promoters based on orientation of Rap1 motifs (red boxes) (left) and corresponding scatterplots of data described in (E) (right).

(H) Similar to (E), but data are separated into Hmo1-dependent or -independent promoters.

See also Figure S2.

motifs in a fluorescent reporter construct that harbors a divergent promoter transcribing *PPT1* in the coding direction and *SUT129* in the noncoding direction (pPS) (Figures 3D and S3A) (Marquardt et al., 2014). A Rap1 motif proximal to the *SUT129* promoter (R1p) lowered yellow fluorescent protein (YFP) levels, while *PPT1* (mCherry) activity increased (Figure 3E). *SUT129* promoter (R1p) activity increased to match control plasmid (pPS) levels upon Rap1 depletion (*RAP1-AID* + IAA). The repression of *SUT129* by Rap1 was not dependent on transcription regulation in the coding direction because in *RAP1-AID* (IAA or NT) cells the *PPT1* signal matched WT (Figure 3E, right panel). The results were comparable using a reporter with Rap1 motifs in the reverse orientation (R1prv) (Figure S3B). Finally, a more distal Rap1 binding site (R1d) to *SUT129* showed comparable YFP levels to the WT reporter, and Rap1 depletion had little effect (Figure 3E). Thus, the Rap1 motif is sufficient to repress divergent noncoding transcription when located near the cryptic promoter sequences.

TSS Mapping of Rap1-Repressed Divergent Noncoding Transcripts

To investigate the relationship between Rap1 motifs and cryptic promoters at a genome-wide scale, we mapped transcription start sites by sequencing (TSS-seq) in WT and Rap1-depleted cells (Figures S4A–S4C). At the *RPL43B* promoter, a cluster of multiple TSSs was detected in a region of 35 bp up- and downstream of the Rap1 motifs in Rap1-depleted cells, and to a lesser extent, in WT cells (Figure 4A, left panel). The signals are unlikely to originate from abortive RNA polymerase II initiation because the TSS-seq procedure isolates poly(A) and capped RNAs. At the *RPL40B* promoter, multiple *iMLP1* TSSs in a region of 23 bp were detected directly upstream of the Rap1 binding site in the *RPL40B* promoter, and the TSS-seq signals increased upon Rap1 depletion (Figure 4A, right panel). Conversely, the *MLP1* protein coding TSS signal decreased in Rap1-depleted cells supporting our observation that Mlp1 protein levels decreased in cells mis-expressing *iMLP1*.

Next, we computed the changes in TSS signals between WT and Rap1-depleted cells. The TSS-seq data matched the RNA-seq data well over a wide range of expression (Figure S4D). ASc1 and ASc2 clusters displayed increased TSS signals around the Rap1 binding sites, whereas there were fewer differences in cluster 3 (ASc3) (Figures 2F and 4B, antisense). As expected, TSS signals decreased in the sense direction downstream of the Rap1 binding sites in Rap1-depleted cells because coding gene expression was reduced (Figure 4B, sense). Interestingly, sequences directly upstream of the canonical coding transcript TSSs displayed increased TSS signals in the sense direction, suggesting that Rap1 is also important for TSS selection (Challal et al., 2018). Finally, most Rap1-regulated promoters examined contained an antisense TSS as the nearest one to the Rap1 binding site (82% antisense, 18% sense). Approximately 50% of the promoters displayed (>2-fold) increased TSS signals within 50 bp of the Rap1 motif in Rap1-depleted cells (Figures 4C and S4E). Thus, Rap1 represses initiation of divergent transcription close to its promoter regulatory elements.

The Rap1 C-Terminal Domain Contributes to Repressing Divergent Transcription

Functional domains of Rap1 are important in gene repression and activation (Azad and Tomar, 2016), so we examined whether repression of divergent transcription requires a specific domain. We generated deletions in the N- and C-terminal domains of Rap1 (Figure 5A). The Rap1 fragments were expressed in *RAP1-AID* cells (Figure S5A). As expected, full-length Rap1 (FL, 1–827) maintained repression of *IRT2* and *iMLP1* expression upon Rap1 depletion (*RAP1-AID* + IAA), whereas the empty vector (EV) control displayed divergent transcription (Figure 5B). A deletion of the N terminus (ΔN, 339–827) rescued Rap1 depletion. Cells harboring deletions in the C terminus (ΔC, 1–599) or N and C termini (ΔN ΔC, 339–599) displayed expression of *IRT2* and *iMLP1*. The Rap1 DNA binding domain represses divergent transcription to some extent because *IRT2* and *iMLP1* expression decreased in ΔN ΔC to ~70% of the EV (Figure S5B).

Important functions of Rap1 are exerted by the C-terminal silencing domain, the activation domain (AD), and the toxicity domain (Tox) (Freeman et al., 1995; Garbett et al., 2007; Layer et al., 2010; Sussel and Shore, 1991). We assessed the ability of Rap1 domain deletion mutants to repress divergent transcription (Figures 5A and S5C–S5E) (Layer et al., 2010). We found that Rap1ΔTox and Rap1ΔAD did not affect *IRT2* and *iMLP1* repression, whereas mutants lacking the DNA binding domain (Rap1ΔDBD), the silencing domain (Rap1Δ764–827), or the AD plus an adjacent sequence (Rap1Δ631–696) failed to repress *IRT2* and *iMLP1* (Figure 5C). Except for Rap1ΔDBD and Rap1Δ764–827, the Rap1 C-terminal mutants associated at the *RPL43B* (*IRT2*) and *RPL40B* (*iMLP1*) promoters (Figures 5D and S5D). We also examined whether different point and patch mutations in the Rap1 silencing domain, already characterized for telomere regulation and hidden mating-type loci silencing, affected repression of *IRT2* expression (Feeser and Wolberger, 2008). We found that none of the mutants caused a significant increase in *IRT2* expression indicating the Rap1 silencing domain is not important for repressing divergent transcription (Table S1). In conclusion, part of Rap1 C terminus, which includes the AD but not the silencing domain, contributes to repression of divergent transcription.

RSC Chromatin Modeler Elicits Divergent Transcription in the Absence of Rap1

Given that the Rap1Δ631–696 mutant displayed divergent transcription but maintained its ability to bind the Rap1 motif, we hypothesized this mutant may associate differently with corepressors or activators of divergent transcription. To identify candidate regulators, we affinity-purified Rap1 from micrococcal nuclease (MNase) solubilized chromatin and used proteomics mass spectrometry to identify associated proteins (Figure 6A) (van Werven et al., 2008). We compared full-length Rap1 (Rap1-FL), Rap1ΔAD, Rap1Δ631–696, and an empty vector (EV) control (Figure S6A; Table S2). Several proteins known to interact with Rap1 were enriched in Rap1-FL versus EV: TAFs, telomere-related proteins, and nuclear pore complex (NPC) proteins (Layer et al., 2010; Van de Vosse et al., 2013) (Figure 6B). In addition, we identified multiple subunits of the RSC complex (12 out of 17). As expected, enriched proteins were involved in

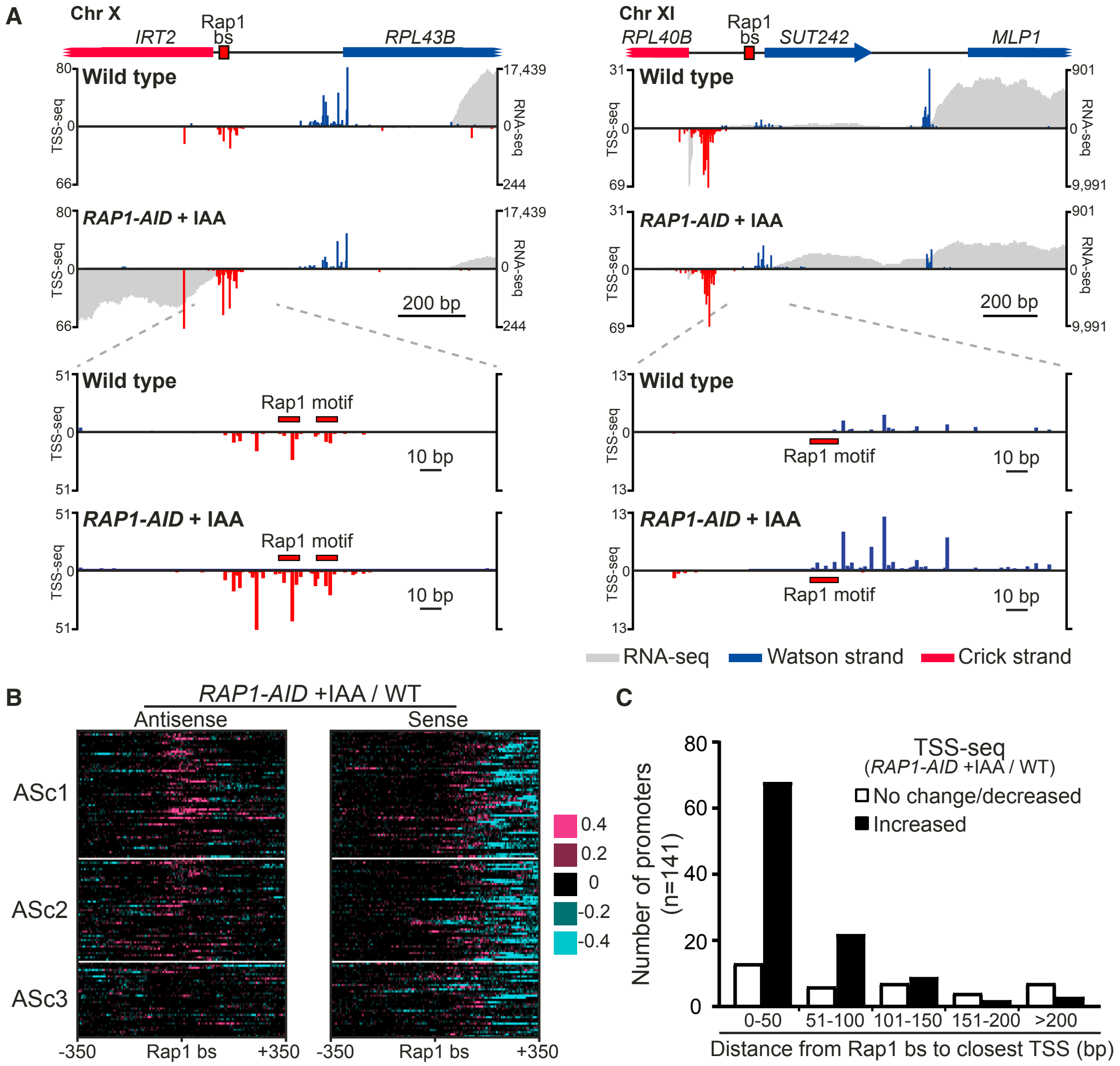

Figure 4. Rap1 Represses Divergent Transcription Close to Its Binding Site

(A) Transcription start sites (TSSs) at *RPL43B* and *RPL40B* in WT (FW629) and *RAP1-AID* + IAA (FW3877) cells. TSS-seq signal per million reads and total RNA-seq data are plotted for both strands.

(B) TSS-seq difference heatmap near 141 promoter Rap1 binding sites ordered as in Figure 2F (ASc1–3). Pink regions depict higher TSS-seq signal (5 bp bin size) in *RAP1-AID* + IAA versus WT; cyan regions depict lower TSS-seq signal.

(C) Distribution of TSSs near promoter Rap1 binding sites from data described in (A) and (B). The distance from the Rap1 binding sites (n = 141) to the closest TSS was measured and their frequency is plotted in bins of 50 bp.

See also Figure S4.

RNA polymerase II transcription and chromatin organization (Figures 6C and S6B). Next, we searched for interacting proteins that showed differential enrichment between Rap1Δ631–696 and Rap1-FL but were not altered in Rap1ΔAD. We found that all identified subunits of RSC were enriched in Rap1Δ631–696 suggesting that Rap1 negatively affects RSC association to the local chromatin environment (Figure 6D).

RSC (remodels the structure of chromatin) is an ATP-dependent chromatin remodeling complex, and it generates NDRs at promoters to facilitate gene activation (Cairns et al., 1996; Clapier et al., 2017). The ATPase subunit of RSC, Sth1, binds near promoter Rap1 binding sites, supporting our observation that RSC interacts with chromatin bound Rap1 (Figures 6E and S6C) (Lopez-Serra et al., 2014; Parnell et al., 2015). RSC interacts with nucleosomes and DNA directly and does not require Rap1 for promoter association or action (Krietenstein et al., 2016; Kubik et al., 2015, 2018). In line with this observation, a narrow NDR is maintained at promoters in the absence of Rap1 (Figures 6F and S6D). It is worth noting that for the clusters with high levels of divergent transcription (ASc1 and ASc2) nucleosomes are highly organized directly upstream of the Rap1 motif, likely due to transcription-coupled chromatin remodeling (Figures 6F and S6D) (Venkatesh and Workman, 2015).

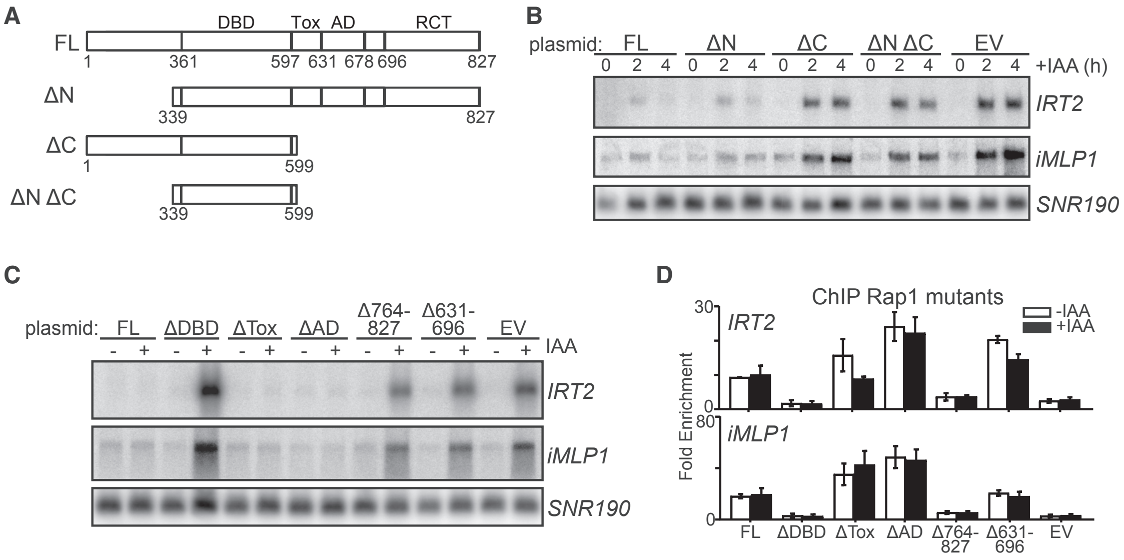

Figure 5. The Rap1 C-Terminal Domain Contributes to Repression of Divergent Transcription

(A) Schematic of Rap1 domains and truncation mutants. DBD, DNA-binding domain; Tox, toxicity domain; AD, activation domain; RCT, Rap1 C-terminal interacting domain.

(B) *IRT2* and *iMLP1* expression in Rap1 mutants detected by northern blot. *RAP1-AID* (FW3877) cells expressing full-length Rap1 (FL) (FW5129), ΔN (FW5133), ΔC (FW5138), ΔN ΔC (FW5141), or an EV (FW5145) were used.

(C) *IRT2* and *iMLP1* expression in different Rap1 domain mutants (FL [FW4948]; ΔDBD [FW4950]; ΔTox [FW4952]; ΔAD [FW4954]; Δ764–827 [FW4958]; Δ631–696 [FW4960]; EV [FW5145]).

(D) Rap1 binding at *RPL43B* (*IRT2*) and *RPL40B* (*iMLP1*) promoters for mutants in (C) (FW5420, FW5393, FW5394, FW5424, FW5395, FW5396, and FW5399), measured by ChIP. Data normalized over *ACT1* and plotted as mean ± SEM (n = 3) are shown.

See also Figure S5.

We hypothesized that RSC promotes divergent transcription in the absence of Rap1. To test this, we depleted Sth1 together with Rap1 (Figure S6E). Depleting Sth1 (*STH1-AID* + IAA) had no effect on *IRT2* and *iMLP1* expression (Figure 6G). When Rap1 and Sth1 were co-depleted, *IRT2* and *iMLP1* expression was greatly reduced compared to Rap1 depletion alone (Figures 6G and S6F). Depleting RSC also suppressed divergent transcription when we used the *PPT1/SUT129* reporter plasmid harboring proximal Rap1 sites (R1p, Figures 6H and S6G). Taken together, we propose that Rap1 reduces RSC association to the local chromatin environment and is positioned to repress divergent noncoding transcription—restricting RSC to stimulate productive coding transcription instead.

Chromatin Regulators Control Divergent Transcription in a Manner Distinct from Rap1

Chromatin remodelers and histone modifying enzymes play essential roles in repressing noncoding transcription (Venkatesh and Workman, 2015). Thus, chromatin regulators may mediate Rap1-dependent repression of divergent transcription. To identify repressors of divergent transcription, we measured *IRT2* and *iMLP1* expression levels in 62 gene deletion and depletion strains (Table S3). Specifically, we selected genes that are (1) involved in cryptic or divergent transcription (e.g., Set2, Set3, and Spt16), (2) known to interact with Rap1 (e.g., Sir3, Rif1, and Rif2), or (3) regulate chromatin and transcription.

Fourteen mutants displayed increased *iMLP1* expression. Only depletion of Spt16 (*SPT16-AID* +IAA) increased *IRT2* expression. When we compared *iMLP1* expression patterns from our data to a published dataset, we found that five mutants overlapped, which we decided to study further (van Bakel et al., 2013). These were (1) putative histone acetyltransferase Spt10, (2) transcription factor Spt21, (3) CAF-1 chromatin assembly complex component Rlf2, (4) chromatin remodeler and elongation factor Spt6, and (5) FACT (facilitates chromatin transcription) complex component Spt16. All candidates have known roles in the repression of divergent or cryptic transcription (Cheung et al., 2008; DeGennaro et al., 2013; Marquardt et al., 2014; Mason and Struhl, 2003). We performed RNA-seq with gene deletion or depletion mutants and observed increased expression within Rap1-regulated promoters (Figures 7A and S7A–S7C).

Next, we examined whether these five chromatin regulators mediate repression of divergent transcription by Rap1. We found little overlap between Rap1-repressed divergent transcripts and transcripts repressed by the chromatin regulators. To illustrate, the *RPL24B* and *RPL40B* promoters showed antisense transcription downstream of the Rap1 motif nearer to or within the coding gene in *rlf2*Δ, *spt10*Δ, and *spt21*Δ cells, and in cells depleted for Spt6 or Spt16 (Figures 7B and S7D). We also identified promoters (*RPL25* and *RPL43B*) that displayed no detectable divergent transcription in the five mutants, while there was a clear signal in Rap1-depleted cells (Figure S7D). The five depletion or deletion mutants displayed increased antisense transcription initiating

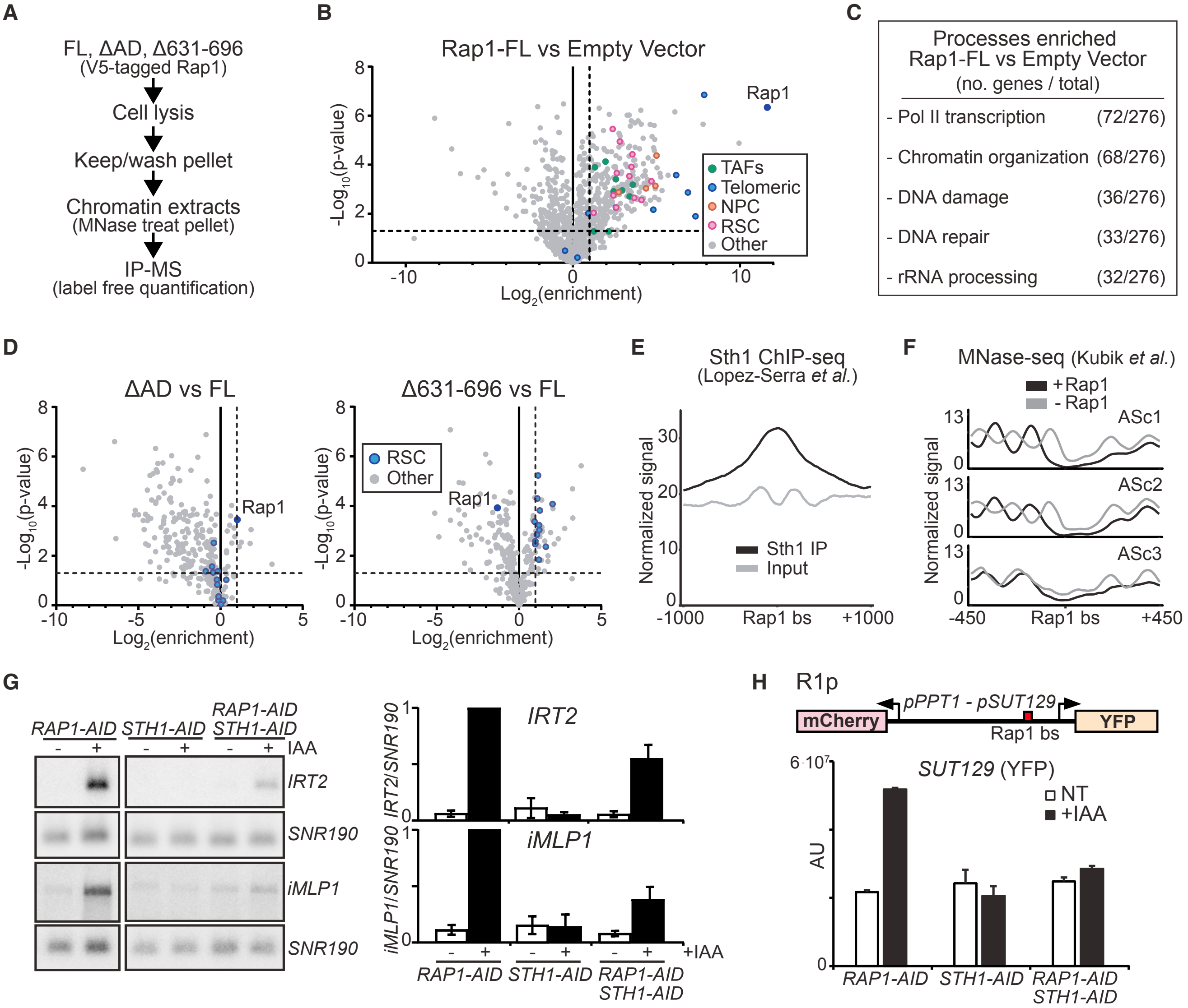

Figure 6. RSC Chromatin Modeler Elicits Divergent Transcription in the Absence of Rap1

(A) Scheme to identify proteins interacting with chromatin bound Rap1. FL (FW5420), ΔAD (FW5424), Δ631–696 (FW5396), and EV (FW5399) were affinity purified and processed for LC-MS label-free quantification (LFQ).

(B) Volcano plot showing differences in protein enrichment for Rap1-V5 (FL versus EV). Enrichment ($\log_2$) versus p value (unpaired two-sample t test, $-\log_{10}$ scale) for n = 916 identified proteins plotted. Horizontal dashed line: 1.303 (p = 0.05); vertical dashed line: 2-fold enrichment.

(C) Yeast GO-Slim Process analysis of data described in (B).

(D) Volcano plots of ΔAD versus FL (left) and Δ631–696 versus FL (right). Proteins that were enriched in FL versus EV (n = 289 proteins) as described in (B) are plotted.

(E) Metagene plots of Sth1 ChIP-seq for Rap1-regulated promoters (n = 141), centered on Rap1 binding sites (GEO: GSE56994) (Lopez-Serra et al., 2014).

(F) Metagene plots of MNase-seq data (GEO: GSE73337) (Kubik et al., 2015) for clusters described in Figure 2F (ASc1–3). The signals in the presence (black) or absence (gray) of Rap1 are displayed.

(G) *IRT2* and *iMLP1* expression in cells depleted for Rap1, Sth1, or both (FW3877; FW6032; FW6231). Membranes were probed for *IRT2*, *iMLP1*, and *SNR190* (left). Quantification of *IRT2* and *iMLP1* expression (right). Mean values ± SEM are plotted (n = 3).

(H) *SUT129* promoter activity upon co-depletion of RSC and Rap1 (FW6206; FW6218; FW6433). Cells were grown, treated, processed, and imaged as described in Figure 3E. Mean signals corrected for background (AU, arbitrary units) were plotted + 95% confidence intervals (n = 50 cells per sample). See also Figure S6.

downstream of the Rap1 binding sites, but not near Rap1 binding sites (Figures 7C and S7E). Taken together, Rap1 acts in concert with chromatin regulators to repress divergent transcription, but in a distinct manner that is spatially limited.

DISCUSSION

Here, we describe how highly expressed coding gene promoters limit divergent noncoding transcription in yeast. We identify a

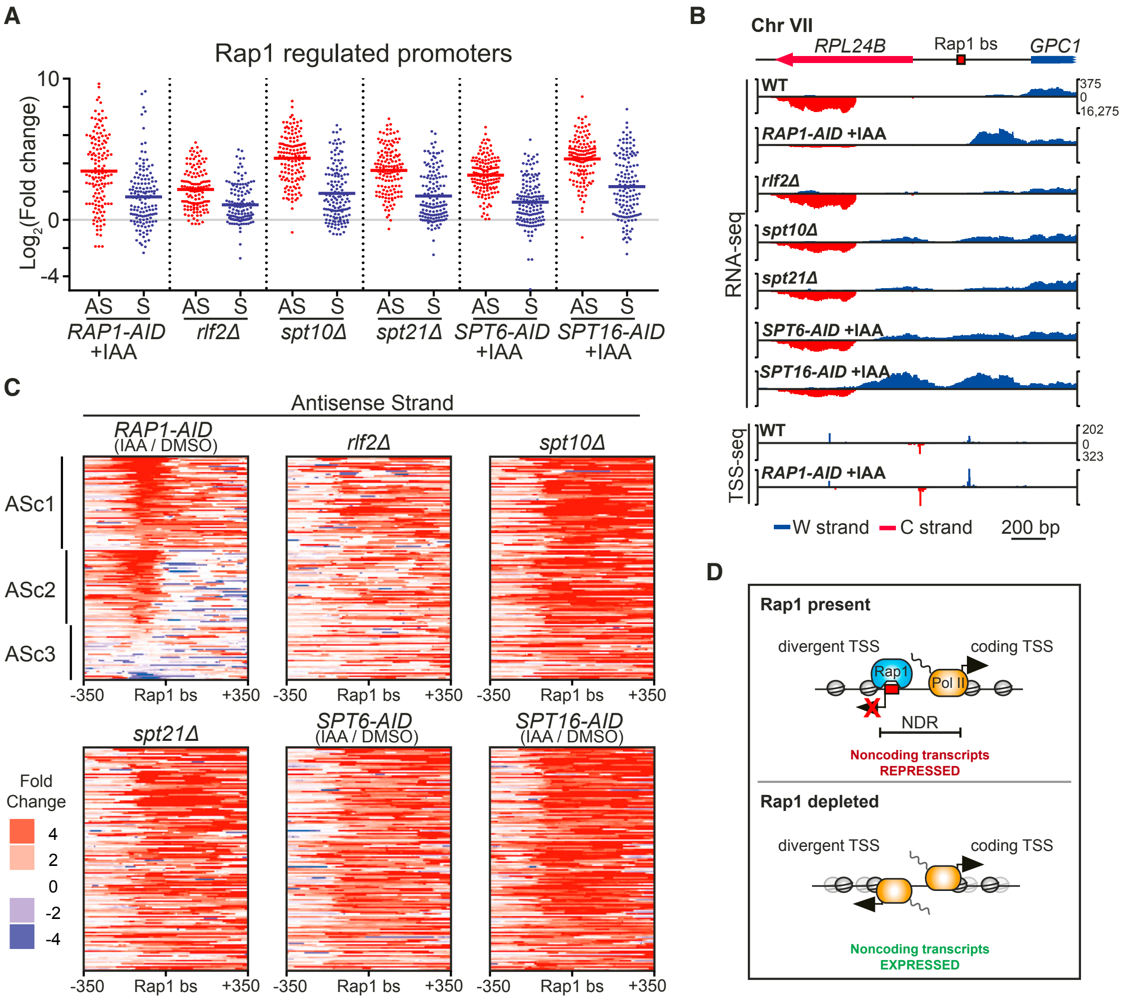

Figure 7. Chromatin Regulators Control Divergent Transcription in a Manner Distinct from Rap1

(A) Scatterplots showing the changes in RNA expression around Rap1 binding sites for *spt10Δ* (FW5543), *spt21Δ* (FW5547), *rlf2Δ* (FW5609) compared to WT (FW629), and for *RAP1-AID* (FW3877), *SPT6-AID* (FW5555), and *SPT16-AID* (FW5559) cells (IAA/DMSO). Changes in RNA expression were calculated for n = 141 promoter Rap1 sites ±100 bp for AS and S strands. Horizontal red or blue bars indicate mean values.

(B) Data from (A) of the *RPL24B* locus including TSS-seq data described in Figure 4.

(C) Heatmaps displaying the data from (A), clustered as in Figure 2F.

(D) Model for Rap1-mediated repression of divergent transcription. Rap1 promotes transcription from the coding direction TSS (black arrows) but represses initiation from the divergent TSS. When Rap1 is absent, transcription in the divergent direction occurs.

See also Figure S7.

surprising role for the pioneer transcription factor Rap1. We find that Rap1 represses divergent noncoding transcription at its binding motif and adjacent sequences. Our data demonstrate how a sequence-specific transcription factor can prevent regulatory sequences from producing aberrant transcripts and define a mechanism for providing directionality toward productive transcription.

Mechanism for Rap1-Mediated Repression of Divergent Transcription

Several lines of evidence indicate that Rap1 represses divergent noncoding transcription, uncoupled from transcription regulation in the coding direction. First, Rap1 represses divergent transcription near the Rap1 binding site. Second, abrogating other transcription factors important for RP gene expression did not affect divergent transcription. Third, close proximity of the Rap1 binding site to the cryptic promoter is essential for repressing divergent transcription. Fourth, the Rap1 binding site ectopically represses divergent noncoding transcription without affecting transcription in the protein-coding direction. Conversely, the AD of Rap1, which directs transcription in the protein-coding direction, is not required for repressing divergent transcription. Finally, we provide evidence that chromatin regulators repress divergent transcription by mechanisms distinct from Rap1.

Promoter directionality is shaped by evolution toward protein-coding genes through enrichment of DNA binding protein motifs

(Jin et al., 2017). In this context, Rap1 promotes directionality in multiple ways. First, Rap1 recruits cofactors and basal transcription machinery, which promote transcription in the coding direction (Azad and Tomar, 2016; Hu and Li, 2007). Second, Rap1 asymmetrically occupies the promoter NDR at the 5′ end, where it represses the divergent core promoter (Figure 7C). Core promoters are intrinsically directional (Duttke et al., 2015), and two independent pre-initiation complexes initiate divergent transcription at mRNA-noncoding RNA pairs in yeast (Rhee and Pugh, 2012). Hence, repressing transcription initiation at the antisense core promoter regulates promoter directionality. As a consequence, upstream regulatory elements or transcription factor binding sites should not overlap with core promoters to avoid concurrent steric interference. In mammalian cells, some pioneer transcription factors also open chromatin asymmetrically (Sherwood et al., 2014)—suggesting that repression of divergent transcription by transcription factors could be broadly conserved.

We found no evidence that Rap1 silencing and roadblock functions, and cofactors of Rap1, are important for repression of divergent transcription (Candelli et al., 2018; Yarrington et al., 2012). Typically, the Rap1 roadblock acts as a failsafe mechanism by terminating upstream transcriptional readthrough toward the downstream coding gene. In contrast, most Rap1-repressed divergent transcripts initiate antisense and upstream of the Rap1 motif, so there is no potential roadblock downstream of the divergent TSS. We find no evidence for contributions from other Rap1 cofactors suggesting that Rap1 itself represses divergent transcription directly. In this context, Rap1-mediated repression of divergent transcription shows parallels to prokaryotic operon regulation and synthetic transcriptional repression systems. In bacteria, transcriptional repressors bind operon sequences near TSSs and directly prevent recruitment of RNA polymerase through steric hindrance (Browning and Busby, 2004). Similarly, Rap1-mediated repression of divergent core promoters could also act through steric hindrance. Like bacterial repressors, Rap1 binds near the TSS of (divergent) core promoters. In eukaryotes, direct steric repression of transcription can be achieved by dCas9 CRISPR interference and transcription activator-like effector repressors (TALERs) when targeted near TSSs (Gilbert et al., 2013; Li et al., 2015).

A Model for Control of Divergent Noncoding Transcription by Rap1

Interestingly, the C terminus of Rap1 contributes to repression of divergent transcription. Perhaps the region we identified (residue 631–696) contributes to exclusion or activity of other factors. The Rap1 C terminus may modulate the affinity and binding mode of the DNA binding domain (Feldmann et al., 2015). Consistent with our observations, a study by Challal et al. also found that Rap1 regulates the fidelity of TSS selection at gene promoters (Challal et al., 2018). It is worth noting that this study showed that the DNA binding domain of Rap1 is, at least in part, sufficient to prevent initiation at aberrant TSSs. Future work should clarify the contributions of the Rap1 DNA-binding and C-terminal domains toward repression of noncoding transcription.

We find that Rap1 controls the activity or action of RSC, because in Rap1-depleted cells RSC elicits divergent transcription. Our data indicate that Rap1 restricts RSC to stimulate productive transcription in the protein-coding direction. Recently, it was shown that RSC helps maintain an NDR in the absence of Rap1 (Kubik et al., 2018). A RSC-dependent NDR could contribute to divergent transcription in the absence of Rap1. We propose that, in WT cells, Rap1 competes locally with the binding of activators of divergent transcription, such as RSC and basal transcription machinery (Figure 7D).

Decades of work have shown that eukaryotes have adopted redundant strategies to limit expression of aberrant noncoding RNAs. Mis-regulation of divergent noncoding transcripts could have negative effects on local or global gene expression, especially in gene-dense genomes such as that in budding yeast. Repression of cryptic transcription nearby regulatory elements, as we have shown for Rap1, could be a conserved property of sequence-specific transcription factors and other DNA binding proteins.

STAR★METHODS

Detailed methods are provided in the online version of this paper and include the following:

- KEY RESOURCES TABLE
- CONTACT FOR REAGENT AND RESOURCE SHARING
- EXPERIMENTAL MODEL AND SUBJECT DETAILS
  - Yeast Strains
  - Growth and Conditions
- METHOD DETAILS
  - Plasmids and Yeast Transformation
  - Fluorescence Microscopy and Quantification
  - Spot Growth Assay
  - RNA Extraction
  - Northern Blot
  - Western Blot
  - Antibodies
  - Chromatin Immunoprecipitation
  - Single Molecule RNA FISH
  - RNA Sequencing Library Preparation
  - TSS Sequencing Library Preparation
  - Differential Expression Analysis
  - TSS-Seq Analysis
  - ChIP-Seq and MNase-Seq Analysis
  - Immunuprecipication of Chromatin-Bound Rap1 and Mass Spectrometry
  - Oligonucleotides Used in This Study
- QUANTIFICATION AND STATISTICAL ANALYSIS
- DATA AND SOFTWARE AVAILABILITY

SUPPLEMENTAL INFORMATION

Supplemental Information includes seven figures and six tables and can be found with this article online at https://doi.org/10.1016/j.molcel.2018.10.018.

ACKNOWLEDGMENTS

We are grateful to Frank Uhlmann, Jesper Svejstrup, and the Van Werven lab for their critical reading of the manuscript. We thank the Crick Advanced Sequencing and Genomics Equipment Park Facilities for experimental

support; Peter Thorpe, Amanda Johnson, Tony Weil, Cynthia Wolberger, Sebastian Marquardt, Jesper Svejstrup for sharing reagents; and Domenico Libri for sharing unpublished data. This work was supported by the Francis Crick Institute (FC001203), which receives its core funding from Cancer Research UK (FC001203), the UK Medical Research Council (FC001203), and the Wellcome Trust (FC001203). M.C. is supported by a fellowship from the Agency for Science, Technology and Research (A*STAR) of Singapore.

AUTHOR CONTRIBUTIONS

F.J.v.W. and A.C.K.W. conceived the project. A.C.K.W. performed most experiments. H.P. and A.C.K.W. performed the bioinformatic analyses. M.C. developed the TSS-seq protocol and prepared the TSS-seq libraries. D.F. and A.P.S. performed and analyzed the mass spectrometry experiment. F.M. performed and analyzed the RNA FISH experiment. A.C.K.W. and F.J.v.W. designed the experiments and wrote the manuscript with input from the other co-authors. F.J.v.W. supervised the project.

DECLARATION OF INTERESTS

The authors declare no competing interests.

Received: May 24, 2018
Revised: September 6, 2018
Accepted: October 10, 2018
Published: December 20, 2018

REFERENCES

Andersson, R., Chen, Y., Core, L., Lis, J.T., Sandelin, A., and Jensen, T.H. (2015). Human gene promoters are intrinsically bidirectional. Mol. Cell *60*, 346–347.

Ard, R., Allshire, R.C., and Marquardt, S. (2017). Emerging properties and functional consequences of noncoding transcription. Genetics *207*, 357–367.

Azad, G.K., and Tomar, R.S. (2016). The multifunctional transcription factor Rap1: a regulator of yeast physiology. Front. Biosci. *21*, 918–930.

Browning, D.F., and Busby, S.J. (2004). The regulation of bacterial transcription initiation. Nat. Rev. Microbiol. *2*, 57–65.

Cairns, B.R., Lorch, Y., Li, Y., Zhang, M., Lacomis, L., Erdjument-Bromage, H., Tempst, P., Du, J., Laurent, B., and Kornberg, R.D. (1996). RSC, an essential, abundant chromatin-remodeling complex. Cell *87*, 1249–1260.

Candelli, T., Challal, D., Briand, J.B., Boulay, J., Porrua, O., Colin, J., and Libri, D. (2018). High-resolution transcription maps reveal the widespread impact of roadblock termination in yeast. EMBO J. *37*, e97490.

Challal, D., Barucco, M., Kubik, S., Feuerbach, F., Candelli, T., Geoffroy, H., Benaksas, C., Shore, D., and Libri, D. (2018). General regulatory factors control the fidelity of transcription by restricting non-coding and ectopic initiation. bioRxiv. https://doi.org/10.1101/331793.

Chen, K., Xi, Y., Pan, X., Li, Z., Kaestner, K., Tyler, J., Dent, S., He, X., and Li, W. (2013). DANPOS: Dynamic analysis of nucleosome position and occupancy by sequencing. Genome Res. *23*, 341–351.

Chen, J., Tresenrider, A., Chia, M., McSwiggen, D.T., Spedale, G., Jorgensen, V., Liao, H., van Werven, F.J., and Ünal, E. (2017). Kinetochore inactivation by expression of a repressive mRNA. eLife *6*, e27417.

Cheng, Z., Otto, G.M., Powers, E.N., Keskin, A., Mertins, P., Carr, S.A., Jovanovic, M., and Brar, G.A. (2018). Pervasive, coordinated protein-level changes driven by transcript isoform switching during meiosis. Cell *172*, 910–923.e16.

Cheung, V., Chua, G., Batada, N.N., Landry, C.R., Michnick, S.W., Hughes, T.R., and Winston, F. (2008). Chromatin- and transcription-related factors repress transcription from within coding regions throughout the Saccharomyces cerevisiae genome. PLoS Biol. *6*, e277.

Chia, M., Tresenrider, A., Chen, J., Spedale, G., Jorgensen, V., Ünal, E., and van Werven, F.J. (2017). Transcription of a 5′ extended mRNA isoform directs dynamic chromatin changes and interference of a downstream promoter. eLife *6*, e27420.

Churchman, L.S., and Weissman, J.S. (2011). Nascent transcript sequencing visualizes transcription at nucleotide resolution. Nature *469*, 368–373.

Clapier, C.R., Iwasa, J., Cairns, B.R., and Peterson, C.L. (2017). Mechanisms of action and regulation of ATP-dependent chromatin-remodelling complexes. Nat. Rev. Mol. Cell Biol. *18*, 407–422.

Cox, J., and Mann, M. (2008). MaxQuant enables high peptide identification rates, individualized p.p.b.-range mass accuracies and proteome-wide protein quantification. Nat. Biotechnol. *26*, 1367–1372.

DeGennaro, C.M., Alver, B.H., Marguerat, S., Stepanova, E., Davis, C.P., Bähler, J., Park, P.J., and Winston, F. (2013). Spt6 regulates intragenic and antisense transcription, nucleosome positioning, and histone modifications genome-wide in fission yeast. Mol. Cell. Biol. *33*, 4779–4792.

Dobin, A., Davis, C.A., Schlesinger, F., Drenkow, J., Zaleski, C., Jha, S., Batut, P., Chaisson, M., and Gingeras, T.R. (2013). STAR: Ultrafast universal RNA-seq aligner. Bioinformatics *29*, 15–21.

Duttke, S.H.C., Lacadie, S.A., Ibrahim, M.M., Glass, C.K., Corcoran, D.L., Benner, C., Heinz, S., Kadonaga, J.T., and Ohler, U. (2015). Human promoters are intrinsically directional. Mol. Cell *57*, 674–684.

Feeser, E.A., and Wolberger, C. (2008). Structural and functional studies of the Rap1 C-terminus reveal novel separation-of-function mutants. J. Mol. Biol. *380*, 520–531.

Feldmann, E.A., De Bona, P., and Galletto, R. (2015). The wrapping loop and Rap1 C-terminal (RCT) domain of yeast Rap1 modulate access to different DNA binding modes. J. Biol. Chem. *290*, 11455–11466.

Freeman, K., Gwadz, M., and Shore, D. (1995). Molecular and genetic analysis of the toxic effect of RAP1 overexpression in yeast. Genetics *141*, 1253–1262.

Garbett, K.A., Tripathi, M.K., Cencki, B., Layer, J.H., and Weil, P.A. (2007). Yeast TFIID serves as a coactivator for Rap1p by direct protein-protein interaction. Mol. Cell. Biol. *27*, 297–311.

Gilbert, L.A., Larson, M.H., Morsut, L., Liu, Z., Brar, G.A., Torres, S.E., Stern-Ginossar, N., Brandman, O., Whitehead, E.H., Doudna, J.A., et al. (2013). CRISPR-mediated modular RNA-guided regulation of transcription in eukaryotes. Cell *154*, 442–451.

Hu, H., and Li, X. (2007). Transcriptional regulation in eukaryotic ribosomal protein genes. Genomics *90*, 421–423.

Jensen, T.H., Jacquier, A., and Libri, D. (2013). Dealing with pervasive transcription. Mol. Cell *52*, 473–484.

Jin, Y., Eser, U., Struhl, K., and Churchman, L.S. (2017). The ground state and evolution of promoter region directionality. Cell *170*, 889–898.e10.

Kent, W.J., Zweig, A.S., Barber, G., Hinrichs, A.S., and Karolchik, D. (2010). BigWig and BigBed: enabling browsing of large distributed datasets. Bioinformatics *26*, 2204–2207.

Knight, B., Kubik, S., Ghosh, B., Bruzzone, M.J., Geertz, M., Martin, V., Dénervaud, N., Jacquet, P., Ozkan, B., Rougemont, J., et al. (2014). Two distinct promoter architectures centered on dynamic nucleosomes control ribosomal protein gene transcription. Genes Dev. *28*, 1695–1709.

Krietenstein, N., Wal, M., Watanabe, S., Park, B., Peterson, C.L., Pugh, B.F., and Korber, P. (2016). Genomic nucleosome organization reconstituted with pure proteins. Cell *167*, 709–721.

Kubik, S., Bruzzone, M.J., Jacquet, P., Falcone, J.L., Rougemont, J., and Shore, D. (2015). Nucleosome stability distinguishes two different promoter types at all protein-coding genes in yeast. Mol. Cell *60*, 422–434.

Kubik, S., O'Duibhir, E., de Jonge, W.J., Mattarocci, S., Albert, B., Falcone, J.L., Bruzzone, M.J., Holstege, F.C.P., and Shore, D. (2018). Sequence-directed action of rsc remodeler and general regulatory factors modulates +1 nucleosome position to facilitate transcription. Mol. Cell *71*, 89–102.e5.

Layer, J.H., Miller, S.G., and Weil, P.A. (2010). Direct transactivator-transcription factor IID (TFIID) contacts drive yeast ribosomal protein gene transcription. J. Biol. Chem. *285*, 15489–15499.

CellPress

Li, B., and Dewey, C.N. (2011). RSEM: accurate transcript quantification from RNA-seq data with or without a reference genome. BMC Bioinformatics *12*, 323.

Li, H., and Durbin, R. (2009). Fast and accurate short read alignment with Burrows-Wheeler transform. Bioinformatics *25*, 1754–1760.

Li, H., Handsaker, B., Wysoker, A., Fennell, T., Ruan, J., Homer, N., Marth, G., Abecasis, G., and Durbin, R.; 1000 Genome Project Data Processing Subgroup (2009). The Sequence Alignment/Map format and SAMtools. Bioinformatics *25*, 2078–2079.

Li, Y., Jiang, Y., Chen, H., Liao, W., Li, Z., Weiss, R., and Xie, Z. (2015). Modular construction of mammalian gene circuits using TALE transcriptional repressors. Nat. Chem. Biol. *11*, 207–213.

Liao, Y., Smyth, G.K., and Shi, W. (2014). featureCounts: an efficient general purpose program for assigning sequence reads to genomic features. Bioinformatics *30*, 923–930.

Lieb, J.D., Liu, X., Botstein, D., and Brown, P.O. (2001). Promoter-specific binding of Rap1 revealed by genome-wide maps of protein-DNA association. Nat. Genet. *28*, 327–334.

Longtine, M.S., McKenzie, A., 3rd, Demarini, D.J., Shah, N.G., Wach, A., Brachat, A., Philippsen, P., and Pringle, J.R. (1998). Additional modules for versatile and economical PCR-based gene deletion and modification in Saccharomyces cerevisiae. Yeast *14*, 953–961.

Lopez-Serra, L., Kelly, G., Patel, H., Stewart, A., and Uhlmann, F. (2014). The Scc2-Scc4 complex acts in sister chromatid cohesion and transcriptional regulation by maintaining nucleosome-free regions. Nat. Genet. *46*, 1147–1151.

Love, M.I., Huber, W., and Anders, S. (2014). Moderated estimation of fold change and dispersion for RNA-seq data with DESeq2. Genome Biol. *15*, 550.

Marquardt, S., Escalante-Chong, R., Pho, N., Wang, J., Churchman, L.S., Springer, M., and Buratowski, S. (2014). A chromatin-based mechanism for limiting divergent noncoding transcription. Cell *157*, 1712–1723.

Martin, M. (2011). Cutadapt removes adapter sequences from high-throughput sequencing reads. EMBnetjournal *17*, 10.

Mason, P.B., and Struhl, K. (2003). The FACT complex travels with elongating RNA polymerase II and is important for the fidelity of transcriptional initiation in vivo. Mol. Cell. Biol. *23*, 8323–8333.

McKnight, J.N., Tsukiyama, T., and Bowman, G.D. (2016). Sequence-targeted nucleosome sliding in vivo by a hybrid Chd1 chromatin remodeler. Genome Res. *26*, 693–704.

Morawska, M., and Ulrich, H.D. (2013). An expanded tool kit for the auxin-inducible degron system in budding yeast. Yeast *30*, 341–351.

Moretto, F., Wood, N.E., Kelly, G., Doncic, A., and van Werven, F.J. (2018). A regulatory circuit of two lncRNAs and a master regulator directs cell fate in yeast. Nat. Commun. *9*, 780.

Neil, H., Malabat, C., d'Aubenton-Carafa, Y., Xu, Z., Steinmetz, L.M., and Jacquier, A. (2009). Widespread bidirectional promoters are the major source of cryptic transcripts in yeast. Nature *457*, 1038–1042.

Nishimura, K., Fukagawa, T., Takisawa, H., Kakimoto, T., and Kanemaki, M. (2009). An auxin-based degron system for the rapid depletion of proteins in nonplant cells. Nat. Methods *6*, 917–922.

Park, D., Morris, A.R., Battenhouse, A., and Iyer, V.R. (2014). Simultaneous mapping of transcript ends at single-nucleotide resolution and identification of widespread promoter-associated non-coding RNA governed by TATA elements. Nucleic Acids Res. *42*, 3736–3749.

Parnell, T.J., Schlichter, A., Wilson, B.G., and Cairns, B.R. (2015). The chromatin remodelers RSC and ISW1 display functional and chromatin-based promoter antagonism. eLife *4*, e06073.

Quinlan, A.R., and Hall, I.M. (2010). BEDTools: a flexible suite of utilities for comparing genomic features. Bioinformatics *26*, 841–842.

Reja, R., Vinayachandran, V., Ghosh, S., and Pugh, B.F. (2015). Molecular mechanisms of ribosomal protein gene coregulation. Genes Dev. *29*, 1942–1954.

Rhee, H.S., and Pugh, B.F. (2011). Comprehensive genome-wide protein-DNA interactions detected at single-nucleotide resolution. Cell *147*, 1408–1419.

Rhee, H.S., and Pugh, B.F. (2012). Genome-wide structure and organization of eukaryotic pre-initiation complexes. Nature *483*, 295–301.

Schneider, C.A., Rasband, W.S., and Eliceiri, K.W. (2012). NIH Image to ImageJ: 25 years of image analysis. Nat. Methods *9*, 671–675.

Scruggs, B.S., Gilchrist, D.A., Nechaev, S., Muse, G.W., Burkholder, A., Fargo, D.C., and Adelman, K. (2015). Bidirectional transcription arises from two distinct hubs of transcription factor binding and active chromatin. Mol. Cell *58*, 1101–1112.

Seila, A.C., Calabrese, J.M., Levine, S.S., Yeo, G.W., Rahl, P.B., Flynn, R.A., Young, R.A., and Sharp, P.A. (2008). Divergent transcription from active promoters. Science *322*, 1849–1851.

Sherwood, R.I., Hashimoto, T., O'Donnell, C.W., Lewis, S., Barkal, A.A., van Hoff, J.P., Karun, V., Jaakkola, T., and Gifford, D.K. (2014). Discovery of directional and nondirectional pioneer transcription factors by modeling DNase profile magnitude and shape. Nat. Biotechnol. *32*, 171–178.

Sussel, L., and Shore, D. (1991). Separation of transcriptional activation and silencing functions of the RAP1-encoded repressor/activator protein 1: isolation of viable mutants affecting both silencing and telomere length. Proc. Natl. Acad. Sci. USA *88*, 7749–7753.

Tyanova, S., Temu, T., Sinitcyn, P., Carlson, A., Hein, M.Y., Geiger, T., Mann, M., and Cox, J. (2016). The Perseus computational platform for comprehensive analysis of (prote)omics data. Nat. Methods *13*, 731–740.

van Bakel, H., Tsui, K., Gebbia, M., Mnaimneh, S., Hughes, T.R., and Nislow, C. (2013). A compendium of nucleosome and transcript profiles reveals determinants of chromatin architecture and transcription. PLoS Genet. *9*, e1003479.

Van de Vosse, D.W., Wan, Y., Lapetina, D.L., Chen, W.M., Chiang, J.H., Aitchison, J.D., and Wozniak, R.W. (2013). A role for the nucleoporin Nup170p in chromatin structure and gene silencing. Cell *152*, 969–983.

van Dijk, E.L., Chen, C.L., d'Aubenton-Carafa, Y., Gourvennec, S., Kwapisz, M., Roche, V., Bertrand, C., Silvain, M., Legoix-Né, P., Loeillet, S., et al. (2011). XUTs are a class of Xrn1-sensitive antisense regulatory non-coding RNA in yeast. Nature *475*, 114–117.

van Werven, F.J., van Bakel, H., van Teeffelen, H.A., Altelaar, A.F., Koerkamp, M.G., Heck, A.J., Holstege, F.C., and Timmers, H.T. (2008). Cooperative action of NC2 and Mot1p to regulate TATA-binding protein function across the genome. Genes Dev. *22*, 2359–2369.

Venkatesh, S., and Workman, J.L. (2015). Histone exchange, chromatin structure and the regulation of transcription. Nat. Rev. Mol. Cell Biol. *16*, 178–189.

Warner, J.R. (1999). The economics of ribosome biosynthesis in yeast. Trends Biochem. Sci. *24*, 437–440.

Winzeler, E.A., Shoemaker, D.D., Astromoff, A., Liang, H., Anderson, K., Andre, B., Bangham, R., Benito, R., Boeke, J.D., Bussey, H., et al. (1999). Functional characterization of the *S. cerevisiae* genome by gene deletion and parallel analysis. Science *285*, 901–906.

Xu, Z., Wei, W., Gagneur, J., Perocchi, F., Clauder-Münster, S., Camblong, J., Guffanti, E., Stutz, F., Huber, W., and Steinmetz, L.M. (2009). Bidirectional promoters generate pervasive transcription in yeast. Nature *457*, 1033–1037.

Xue, Y., Pradhan, S.K., Sun, F., Chronis, C., Tran, N., Su, T., Van, C., Vashisht, A., Wohlschlegel, J., Peterson, C.L., et al. (2017). Mot1, Ino80C, and NC2 function coordinately to regulate pervasive transcription in yeast and mammals. Mol. Cell *67*, 594–607.e4.

Yarrington, R.M., Richardson, S.M., Lisa Huang, C.R., and Boeke, J.D. (2012). Novel transcript truncating function of Rap1p revealed by synthetic codon-optimized Ty1 retrotransposon. Genetics *190*, 523–535.

STAR★METHODS

KEY RESOURCES TABLE

REAGENT or RESOURCE	SOURCE	IDENTIFIER
Antibodies		
Anti-V5 tag (mouse) antibody	Thermo Fisher Scientific	R96025; RRID: AB_2556564
Anti-hexokinase (rabbit) antibody	US Biological	H2035; RRID: AB_2629457
Anti-Myc tag (mouse) antibody	Merck Millipore	05-724; RRID: AB_309938
Anti-HA tag (mouse) antibody	This paper	12CA5
Anti-FLAG tag (mouse) antibody	Sigma-Aldrich (Merck)	F3165; RRID: AB_259529
Anti-mouse IgG HRP-linked antibody	GE Life Sciences	NA931V; RRID: AB_772210
Anti-rabbit IgG HRP-linked antibody	GE Life Sciences	NA934V; RRID: AB_772206
Anti-V5 agarose affinity gel	Sigma-Aldrich (Merck)	A7345; RRID: AB_10062721
Chemicals, Peptides, and Recombinant Proteins		
Indole-3-acetic acid (IAA, auxin)	Sigma-Aldrich (Merck)	I3750
ULTRAhyb Ultrasensitive Hybridization Buffer	Thermo Fisher Scientific	AM8670
Prime-It II Random Primer Labeling Kit	Agilent	300385
dATP [α-32P]	PerkinElmer	NEG512H500UC
dATP [α-32P]	Hartmann Analytic	SRP-203
Proteinase K	Thermo Fisher Scientific	EO0491
rDNase	Machery-Nagel	740963
Micrococcal Nuclease (MNase)	NEB	M02475
RNA Fragmentation Reagents (Ambion)	Thermo Fisher Scientific	AM8740
Shrimp Alkaline Phosphatase (rSAP)	NEB	M0371L
Cap-Clip Acid Pyrophosphatase	CellScript	C-CC15011H
T4 RNA ligase 1 (high concentration)	NEB	M0437M
SuperScript IV Reverse Transcriptase	Thermo Fisher Scientific	18090050
RNasin Plus Ribonuclease Inhibitor	Promega	N2115
RNase H	NEB	M0297L
RNase cocktail	Thermo Fisher Scientific	AM2286
Dynabeads MyOne Streptavidin C1	Thermo Fisher Scientific	65002
NotI-HF	NEB	R3189M
T3 RNA polymerase	NEB	M0378S
Vaccinia capping enzyme	NEB	M2080S
TURBO DNase	Thermo Fisher Scientific	AM2238
Linear acrylamide	Thermo Fisher Scientific	AM9520
IME1 and *ACT1* single molecule RNA-FISH probes	Biosearch Technologies	N/A
Critical Commercial Assays		
PowerUp SYBR Green Master Mix	Thermo Fisher Scientific	A25742
Amersham ECL Prime Western Blotting Detection Reagent	GE Life Sciences	RPN2232
Ribo-Zero Gold rRNA Removal Kit (Yeast)	Illumina	MRZY1324
TruSeq Stranded Total RNA	Illumina	RS-122-2202
TruSeq Stranded mRNA	Illumina	RS-122-2101
Poly(A)Purist MAG Kit	Thermo Fisher Scientific	AM1922
2100 Bioanalyzer	Agilent	G2939BA
HighPrep PCR	MagBio	AC-60050
Qubit dsDNA HS Assay Kit	Thermo Fisher Scientific	Q32851
KAPA HiFi HotStart ReadyMix PCR Kit	KAPA Biosystems (Roche)	KK2602

(*Continued on next page*)

Continued

REAGENT or RESOURCE	SOURCE	IDENTIFIER
KAPA Hyper Prep Kit	KAPA Biosystems (Roche)	KK8504
KAPA SI Adaptor Kit Set A+B (30 uM)	KAPA Biosystems (Roche)	KK8700
RNeasy MinElute Cleanup Kit	QIAGEN	74204
Deposited Data		
Total, poly(A), and TSS RNA sequencing	This paper	GEO: GSE110004
Sth1 ChIP-seq	Lopez-Serra et al., 2014	GEO: GSE56994
Sth1 MNase ChIP-seq	Parnell et al., 2015	GEO: GSE65594
MNase-seq	Kubik et al., 2015	GEO: GSE73337
Experimental Models: Organisms/Strains		
S. cerevisiae: Strain background: BY, see Table S4	This paper	N/A
Oligonucleotides		
Oligonucleotides and primers, see Table S6	This paper	N/A
Recombinant DNA		
Plasmids, see Table S5	This paper	N/A
Software and Algorithms		
Cutadapt (version 1.9.1)	Martin, 2011	https://cutadapt.readthedocs.io/en/stable/
RSEM (version 1.3.0)	Li and Dewey, 2011	https://github.com/deweylab/RSEM
STAR (version 2.5.2a)	Dobin et al., 2013	https://github.com/alexdobin/STAR
DESeq2 (version 1.12.3)	Love et al., 2014	https://doi.org/10.18129/B9.bioc.DESeq2
BWA (version 0.5.9-r16)	Li and Durbin, 2009	http://bio-bwa.sourceforge.net/
DANPOS2 (version 2.2.2)	Chen et al., 2013	https://sites.google.com/site/danposdoc/
Subread (version 1.5.1)	Liao et al., 2014	http://subread.sourceforge.net/
SAMTools (version 1.3.1)	Li et al., 2009	http://www.htslib.org/
BEDTools (version 2.26.0)	Quinlan and Hall, 2010	https://bedtools.readthedocs.io/
BigWig and BigBed	Kent et al., 2010	http://hgdownload.soe.ucsc.edu/admin/exe/linux.x86_64/
MaxQuant (version 1.6.01)	Cox and Mann, 2008	http://www.coxdocs.org/doku.php?id=:maxquant:start
Perseus (version 1.4.0.2)	Tyanova et al., 2016	http://www.coxdocs.org/doku.php?id=:perseus:start
ImageJ (version 1.48k)	Schneider et al., 2012	https://imagej.nih.gov/ij/index.html
SGD Gene Ontology Slim Mapper	Saccharomyces Genome Database	https://www.yeastgenome.org/cgi-bin/GO/goSlimMapper.pl

CONTACT FOR REAGENT AND RESOURCE SHARING

Further information and requests for resources and reagents should be directed to the Lead Contact, Folkert van Werven (folkert.vanwerven@crick.ac.uk).

EXPERIMENTAL MODEL AND SUBJECT DETAILS

Yeast Strains

Strains isogenic to the *Saccharomyces cerevisiae* BY strain background were used throughout this study. The genotypes are listed in Table S4. Gene deletions were generated using the one-step disruption protocol as described previously (Longtine et al., 1998). The gene deletion strains used to examine mis-regulation of *IRT2* and *iMLP1* expression in Table S4 were described previously (Winzeler et al., 1999).

Growth and Conditions

Cells were grown in YPD media (1% w/v yeast extract, 2% w/v peptone, 2% w/v glucose, supplemented with tryptophan (96 mg/L), uracil (24 mg/L) and adenine (12 mg/L). Cells were cultured with shaking in conical flasks at 30°C. For single molecule RNA FISH

experiments described in Figure 1, diploid cells were grown to saturation in nutrient-rich YPD media, then shifted to sporulation media (SPO, 0.3% w/v potassium acetate and 0.02% w/v raffinose) to OD_{600} 1.8 and immediately fixed with formaldehyde (3% v/v). Cells were collected immediately after resuspending in SPO media.

For auxin induced depletion experiments, 3-indole-acetic acid (IAA) (Sigma-Aldrich) was used to induce AID-tagged protein depletion. 1 M IAA stocks were prepared in dimethyl sulfoxide (DMSO) and added directly to cultures to a final concentration of 500 μM.

METHOD DETAILS

Plasmids and Yeast Transformation

A one-step tagging procedure was used for generating C-terminal auxin-inducible degron (AID) alleles (*RAP1-AID, FHL1-AID, IFH1-AID, SFP1-AID, SPT6-AID, SPT16-AID, and STH1-AID*), which contains three copies of the V5 epitope and the IAA7 degron (Nishimura et al., 2009). The *RAP1-AID-MYC* allele harbors nine copies of the Myc epitope and *IAA17* residues 71-114 (AID-MYC), and *STH1-AID-FLAG* allele contains six copies of the FLAG epitope and *IAA17* residues 71-114 (AID-FLAG) (Morawska and Ulrich, 2013). The AID strains also contained a single copy integration plasmid expressing *Oryza sativa* TIR1 (*osTIR1*) ubiquitin E3 ligase from the *GPD1* promoter (gift from Leon Chan). *osTIR1* plasmids were linearized by digestion with PmeI and integrated at either the *HIS3* or *LEU2* locus.

All Rap1 mutants and truncation constructs were expressed from single copy integration plasmids (gift from Wendell Lim) in *RAP1-AID* or *RAP1-AID-MYC* strain backgrounds. The truncation or domain deletion mutants described in Figure 5 were cloned from *RAP1* plasmids (gift from Amanda Johnson and Tony Weil) by NotI and XhoI digestion into plasmid 372 to generate plasmids 471-474, and by SacI and KpnI digestion to generate plasmids 477-483 (Garbett et al., 2007; Layer et al., 2010). Three copies of the V5 epitope tag from strain 4732 were introduced by Gibson-style cloning (NEBuilder HiFi, NEB) at the C terminus of Rap1 in plasmids 477-483 to generate plasmids 558, 559, 561, 562, 566, and 568 used for ChIP in Figure 5D.

Single copy integration *RAP1* expression plasmids containing C-terminal point and patch mutations were re-cloned from plasmids described previously (gift from Cynthia Wolberger) by Gibson-style cloning (Feeser and Wolberger, 2008). In short, plasmid 471 was linearized by PCR to allow cloning of homologous Rap1 C terminus fragments containing point and patch mutations. Rap1 plasmids were then linearized by digestion with PmeI and integrated at the *HIS3* locus.

The mCherry-YFP *pPPT1-pSUT129* fluorescent reporter plasmid was described previously (gift from Sebastian Marquardt) (Marquardt et al., 2014). The Rap1 transcription factor binding sites from the *RPL43B* promoter were cloned into unique SspI (proximal) or XmnI (distal) restriction sites. The *PPT1-SUT129* locus was replaced by digesting the plasmid with EcoRI and integrating the reporter construct by transformation as described above. All plasmids are listed in Table S5.

Fluorescence Microscopy and Quantification

Cells were grown in YPD to the exponential phase and fixed with formaldehyde (3.7% w/v) for 15 min. Fixed cells were washed with phosphate-sorbitol buffer (0.1 M KP_i (pH 7), 0.05 M $MgCl_2$, 1.2 M sorbitol), and resuspended in phosphate-sorbitol buffer before imaging. Imaging was performed using a 100x oil objective, NA 1.4, and SOLA SE light engine (Lumencor) on a Nikon Eclipse Ti-E imaging system (Nikon). We used 500 ms exposure time using GFP and mCherry filters to quantify YFP and mCherry levels, respectively. An ORCA-FLASH 4.0 camera (Hamamatsu) and NIS-Elements AR software (Nikon) were used to collect images.

Whole cell fluorescence signals were obtained for YFP and mCherry channels using ImageJ software (NIH) (Schneider et al., 2012). ROIs were manually drawn around the periphery of each cell. The mean intensity in each channel per cell was multiplied by the cell area to obtain mean signal. The signal for each channel was corrected for cell-free background fluorescence in a similar way. Autofluorescence signal was also determined as described for wild-type cells. For the analyses, 50 cells were quantified per sample.

Spot Growth Assay

Cells were grown to saturation in YPD media, then diluted to OD_{600} 0.4 in sterile water. Serial dilutions (5-fold) were spotted onto YPD agar plates in the presence of IAA or DMSO. Cells were incubated at 30°C for 2 days before imaging.

RNA Extraction

Yeast cells were collected from cultures by centrifugation, washed with sterile water, and snap-frozen in liquid nitrogen. RNA was extracted from yeast cell pellets using Acid Phenol:Chloroform:Isoamyl alcohol (125:24:1, Ambion) and precipitated in ethanol with 0.3 M sodium acetate. RNA was resuspended in DEPC-treated sterile water.

Northern Blot

Northern blots were performed as previously described (Chia et al., 2017). RNA samples were denatured in denaturation buffer (1 M deionized glyoxal, 50% v/v DMSO, 10 mM sodium phosphate (NaP_i) buffer (pH 6.8)) at 70°C for 10 min. Denatured samples were mixed with loading buffer (10% v/v glycerol, 2 mM NaP_i buffer, 0.4% w/v bromophenol blue) and separated on an agarose gel (1.1% v/v agarose, 0.01 M NaP_i buffer) by electrophoresis for 2 hr at 80 Volts. Total RNA was transferred onto positively charged nylon membranes (GE Amersham Hybond N+) by capillary transfer and rRNA bands were visualized by methylene blue staining (0.02% w/v methylene blue, 0.3 M sodium acetate).

The membranes were blocked for at least 3 hr at 42°C in hybridization buffer (1% w/v SDS, 40% v/v deionized formamide, 25% w/v dextran sulfate, 58 g/L NaCl, 200 mg/L sonicated salmon sperm DNA (Agilent), 2 g/L BSA, 2 g/L polyvinyl-pyrolidone, 2 g/L Ficoll 400, 1.7 g/L pyrophosphate, 50 mM Tris pH 7.5) or ULTRAhyb Ultrasensitive Hybridization Buffer (Thermo Fisher Scientific) before hybridization. The radioactively labeled probes were synthesized using a Prime-it II Random Primer Labeling Kit (Agilent), 25 ng of target-specific DNA template, and dATP [α-^{32}P] (Perkin-Elmer or Hartmann Analytic). The oligonucleotide sequences used to generate target-specific DNA templates for *IRT2*, *iMLP1*, and *SNR190* northern blot probes are found in Table S6. After overnight hybridization at 42°C, blots were washed for 30 min at 65°C with each of the following: 2X saline-sodium citrate (SSC) buffer, 2X SSC + 1% w/v SDS, 1X SSC + 1% SDS, and 0.5X SSC + 1% SDS. Membranes were exposed to phosphorimaging screens before scanning using Typhoon 9400, FLA 9500, or FLA 7000 instruments (GE Healthcare Life Sciences). For re-probing, membranes were washed with stripping buffer (1 mM Tris, 0.1 mM EDTA, 0.5% SDS) at 85°C until negligible residual signal remained on the membrane.

IRT2, *iMLP1*, and *SNR190* levels were estimated from northern blots using ImageJ (Schneider et al., 2012). The net intensity of each region of interest was determined by subtracting the mean background intensity of the areas immediately above and below the region of interest from the intensity of the main band(s). Signals were first normalized to *SNR190* levels, and then further normalized to a specific band on the same membrane.

Western Blot

Western blots were performed as previously described (Chia et al., 2017). Protein extracts were prepared using the trichloroacetic acid (TCA) extraction protocol. Cells were collected by centrifugation and re-suspended in cold 5% w/v TCA for at least 10 min. Samples were washed with acetone, then completely air-dried. Cells were resuspended with protein breakage buffer (50 mM Tris (pH 7.5), 1 mM EDTA, 2.75 mM dithiothreitol (DTT)) and disrupted using 0.5 mm glass beads and a Mini Beadbeater (Biospec). Two volumes of protein extract were mixed with 1 volume of SDS-PAGE sample buffer (187.5 mM Tris (pH 6.8), 6.0% v/v β-mercaptoethanol, 30% v/v glycerol, 9.0% v/v SDS, 0.05% w/v Bromophenol blue) and denatured at 95°C for 5 min. After SDS-polyacrylamide gel electrophoresis (4%–20% gradient), proteins were transferred onto PVDF membranes. The membranes were blocked in blocking buffer (1% w/v BSA, 1% w/v non-fat powdered milk in phosphate buffered saline with 0.01% v/v Tween-20 (PBST) buffer) before incubation with primary antibodies in blocking buffer overnight at 4°C. Membranes were washed in PBST buffer and incubated with anti-mouse or anti-rabbit IgG HRP-linked antibodies in blocking buffer. Protein levels were detected using Amersham ECL Prime detection reagent and an Amersham Imager 600 instrument (GE Healthcare).

Antibodies

The following antibodies were used for western blotting (also see Key Resources Table). Anti-V5 mouse monoclonal IgG_{2A} (1:2000, Thermo Fisher Scientific R96025, previously Invitrogen 46-0705), anti-hexokinase rabbit (1:8000, US Biological H2035), anti-Myc tag mouse monoclonal (1:2000, Merck Millipore CAT 05-724 Lot DAM1764400), anti-HA tag mouse IgG (1:2000, clone 12CA5), anti-FLAG tag monoclonal mouse IgG_1 (1:2000, Sigma-Aldrich F3165), anti-mouse IgG antibody HRP-linked (1:10000, GE healthcare NA931V5), anti-rabbit IgG antibody HRP-linked (1:10000, GE healthcare NA934V).

Chromatin Immunoprecipitation

ChIP experiments were performed as previously described (Chia et al., 2017). In short, cells were fixed with 1% v/v formaldehyde for 20 min at room temperature and reactions were quenched with glycine (100 mM). Cells were washed once with FA lysis buffer (0.05 M HEPES-KOH (pH 7.5), 0.15 M NaCl, 0.001 M EDTA (pH 8), 1% v/v Triton X-100, 0.1% w/v sodium deoxycholate, 0.1% w/v SDS) and snap-frozen with liquid nitrogen. Cell pellets were disrupted using a Mini Beadbeater and zirconia/silica beads (0.5 mm, Biospec), and cross-linked chromatin extracts were sheared by sonication using a Bioruptor (Diagenode, 9 cycles of 30 s on/off, high intensity). Extracts were incubated for 2 hr at room temperature with 20 μL of anti-V5 antibodies conjugated to agarose beads (Sigma-Aldrich). Subsequently, reverse-crosslinking was performed in TE-SDS buffer (10 mM Tris (pH 8), 1 mM EDTA, 1.0% w/v SDS) at 65°C overnight, samples were treated with Proteinase K (Thermo Fisher Scientific), and DNA fragments were purified by spin column (Machery-Nagel). ChIP signals at *RPL43B* and *RPL40B* promoters were determined by qPCR using PowerUp SYBR Green Master Mix (Thermo Fisher Scientific) and a QuantStudio 3 instrument (Applied Biosystems). As a negative control, we used a primer pair directed to the *ACT1* ORF 3′ end. The oligonucleotide sequences used for ChIP experiments are in Table S6.

Single Molecule RNA FISH

Single molecule RNA fluorescence *in situ* hybridization (FISH) was performed as previously described (Moretto et al., 2018). In short, cells were fixed with formaldehyde overnight, treated with zymolyase and further fixed in 80% v/v ethanol. Subsequently, cells were hybridized with fluorophore-labeled probes (Biosearch Technologies) directed to *IME1* (AF594) and the internal control *ACT1* (Cy5) (Dyes, Thermo Fisher Scientific). Cells were imaged using a 100x oil objective, NA 1.4, and SOLA SE light engine (Lumencor) on a Nikon Eclipse Ti-E imaging system (Nikon). DIC, DAPI, AF594 (*IME1*), and Cy5 (*ACT1*) images were collected every 0.3 micron (20 stacks) using an ORCA-FLASH 4.0 camera (Hamamatsu) and NIS-Elements AR software (Nikon). ImageJ was used to generate maximum intensity Z projections of the images (Schneider et al., 2012). Subsequently, StarSearch software (Raj laboratory,

University of Pennsylvania, http://rajlab.seas.upenn.edu/StarSearch/launch.html) was used to quantify transcripts in single cells. Comparable thresholds were used to count RNA foci in single cells. Only cells positive for the internal control *ACT1* were quantified for analysis (n = 139 cells).

RNA Sequencing Library Preparation

Total RNA from yeast was incubated with rDNase (Machery-Nagel) and column purified (Machery-Nagel) prior to sequencing library preparation. 1 μg of intact yeast total RNA was depleted of ribosomal RNA (rRNA) using a commercial kit (Illumina RiboZero Gold rRNA Removal Kit (Yeast)) for total RNA sequencing and 500 ng of RNA was used for polyadenylated (polyA) RNA sequencing. Libraries were prepared using the TruSeq Stranded Total RNA kit or TruSeq stranded mRNA kit (Illumina) according to the manufacturer's instructions (10 or 13 PCR cycles). Each library was sequenced on the HiSeq 2500 or 4000 platform (Illumina) and generated ~45 million 101 bp strand-specific paired-end reads per sample, on average.

TSS Sequencing Library Preparation

To obtain libraries representing the 5′ ends of polyadenylated and capped transcripts (TSS-seq), approximately 7-9 μg of poly(A)$^+$ RNA together with *in vitro* spike-ins was first subjected to zinc-mediated fragmentation (Ambion) at 70°C. The reaction was subsequently cleaned up using RNeasy MinElute columns (QIAGEN) to isolate RNA fragments with mode length of ~200 nucleotides. These fragments were incubated with shrimp alkaline phosphatase (rSAP, NEB) at 37°C to remove the 5′ phosphate groups of non-capped fragments, followed by acid phenol/chloroform extraction and ethanol precipitation as described above. With the exception of a "no decapping" control sample from wild-type (WT, FW629) cells, dephosphorylated fragments were next treated with Cap-Clip Acid Pyrophosphatase (CellScript) to remove the 5′-terminal caps from fragments representing the bona fide 5′ ends of transcripts. After another round of acid phenol/chloroform extraction and ethanol precipitation, all samples were treated with T4 RNA ligase 1 (NEB) to introduce a custom adaptor sequence to the 5′ uncapped ends of fragments. Excess adapters were removed via a column clean up step. First strand cDNA synthesis was performed using Superscript IV Reverse Transcriptase (Thermo Fisher Scientific), and second strand synthesis was performed using a KAPA HiFi HotStart ReadyMixPCR Kit (KAPA Biosystems) after RNase H (NEB) and RNase cocktail digestion (Ambion). Double-stranded cDNA was quantified by Qubit fluorometric quantitation (Thermo Fisher Scientific) and used as inputs for library preparation using a KAPA Hyper Prep Kit (KAPA Biosystems) and KAPA Single-Indexed adapters for Illumina platforms (KAPA Biosystems). Libraries were quantified by Qubit and sequenced on the HiSeq 4000 platform (Illumina), and typically generated ~39 million 76bp strand-specific single-end reads per sample. ~16 million single-end reads were generated from the "No decapping" control library.

Differential Expression Analysis

Adaptor trimming was performed with cutadapt (version 1.9.1) with parameters "–minimum-length=25–quality-cutoff=20 -a AGATCGGAAGAGC -A AGATCGGAAGAGC" (Martin, 2011). The RSEM package (version 1.3.0) (Li and Dewey, 2011) in conjunction with the STAR alignment algorithm (version 2.5.2a) (Dobin et al., 2013) was used for the mapping and subsequent gene-level counting of the sequenced reads with respect to all *S. cerevisiae* genes downloaded from the Ensembl genome browser (assembly R64-1-1, release 90). The parameters used were "–star-output-genome-bam–forward-prob 0," and all other parameters were kept as default. Differential expression analysis was performed with the DESeq2 package (version 1.12.3) (Love et al., 2014) within the R programming environment (version 3.3.1).

A list of experimentally determined Rap1 sites was obtained from a high-resolution ChIP-exo dataset (Rhee and Pugh, 2011). For differential expression analysis with varying window sizes (e.g., ± 50 bp to ± 500 bp), sites within 500 bp of chromosome ends were removed. To determine the list of 141 well annotated Rap1-regulated genes, we combined lists of ribosomal protein (RP) genes (Reja et al., 2015) and previously identified Rap1-regulated glycolytic pathway genes (Lieb et al., 2001), and removed the RP genes regulated by Abf1 (instead of Rap1). We manually assigned the corresponding promoter Rap1 site to each Rap1-regulated gene from the ChIP-exo dataset. If ChIP-exo coordinates were missing, the Rap1 motif coordinate identified from from Lieb et al. was assigned instead (Lieb et al., 2001). STAR genomic alignments were filtered to only include those that were unspliced, primary, uniquely mapped, properly paired, and had a maximum insert size of 500bp. Fragment counts within specified windows (e.g., ± 100 bp) around the 564 Rap1 sites (1128 intervals total, Watson and Crick strand alignments were assigned to separate intervals) were obtained using the featureCounts tool from the Subread package (version 1.5.1) (Liao et al., 2014). The parameters used were "-O–minOverlap 1–nonSplitOnly–primary -s 2 -p -B -P -d 0 -D 600 -C." Windows on separate strands were treated as separate intervals for all strand-specific RNA-seq experiments, and only reads which overlapped with the corresponding strand and interval were counted. Differential expression analysis around Rap1 binding sites was performed as described in the section above, however, the DESeq2 size factors with respect to the transcriptome were used to normalize the per-sample counts. The same strategy was employed to perform analysis for promoter regions of Ume6-regulated genes (McKnight et al., 2016). Ume6 sites were approximated −250 bp relative to the annotated start of Ume6-regulated genes. The ggplot2 package (version 2.2.1) was used within the RStudio programming environment (version 3.4.0) to generate violin and box-and-whisker plots. The calculated $\log_2$(Fold change) values from DESeq2 analysis were plotted using the geom_violin (scale = "count") and geom_boxplot functions (outlier data points not shown in boxplot

but included in violin plot). Volcano and scatterplots were generated using the geom_point function in ggplot2 or Graphpad Prism (version 7.02). Screenshots of RNA-seq and TSS-seq data were taken using the Integrative Genomics Viewer (Broad Institute, version 2.3.75).

TSS-Seq Analysis

Adaptor trimming was performed with cutadapt (version 1.9.1) (Martin, 2011) with parameters "–minimum-length=20–quality-cutoff=20 -a AGATCGGAAGAGC." The custom 5′ adaptor sequence specific to the protocol was removed by re-running cutadapt with the parameters "–minimum-length=20–quality-cutoff=20 -g GCACTCTGAGCAATACC," and only the reads containing the adaptor sequence were used for further analysis. BWA (version 0.5.9-r16) (Li and Durbin, 2009) using default parameters was used to perform the read mapping to the *S. cerevisiae* genome (assembly R64-1-1, release 90). Uniquely mapped alignments corresponding to the sense and antisense strands were obtained using SAMtools view (version 1.3.1) by using the flags "-q 1 -F 20" and "-q 1 -f 16," respectively (Li et al., 2009). BedGraph coverage tracks representing the TSS-seq signal per million mapped reads were generated using BEDTools genomeCoverageBed (version 2.26.0) (Quinlan and Hall, 2010) with the parameters "-bg −5 -scale <SCALE_FACTOR>." BedGraph files were converted to bigWig using the wigToBigWig binary available from the UCSC with the "-clip" parameter (Kent et al., 2010). Coverage tracks from three biological replicates for each sample were merged for plotting. TSS annotations were obtained from Ensembl assembly R64-1-1, release 90 and annotated SMORE-seq TSSs described previously (Park et al., 2014). To calculate TPM values for each TSS, TSS-seq counts were obtained by quantifying the abundance of reads with the 1st transcribed 5′ nucleotide within ± 75 bp of annotated TSSs (Park et al., 2014), on the respective strand. For the analysis in Figure 4C, the closest cryptic TSS to the Rap1 binding site was annotated manually, and distance was measured from the Rap1 binding site to the mode TSS cluster peak. Differential expression analysis was performed using DESeq2, normalized by sequencing depth. A $\log_2$(fold change) value > 1 (fold change > 2), comparing *RAP1-AID* +IAA over wild-type cells, was considered an increase.

ChIP-Seq and MNase-Seq Analysis

Publicly available datasets for Sth1 ChIP-seq (GEO: GSE56994) (Lopez-Serra et al., 2014), Sth1 MNase ChIP-seq (GEO: GSE65594) (Parnell et al., 2015), and MNase-seq (GEO: GSE73337) (Kubik et al., 2015) described previously were obtained from GEO. ChIP-seq and MNase-seq reads were adaptor-trimmed using cutadapt as specified previously. Genome-wide mapping of the trimmed reads was performed with BWA (version 0.5.9-r16) (Li and Durbin, 2009) using default parameters. Single-end ChIP-seq alignments were filtered to remove duplicate and multi-mapped reads. Paired-end MNase-seq alignments were filtered to only include those that were properly paired, uniquely mapped, had a maximum of two mismatches in either read, and an insert size within the range 120 - 200 bp. Genome-wide nucleosome coverage profiles were obtained using the DANPOS2 dpos (version 2.2.2) (Chen et al., 2013) command with parameters "–span 1–smooth_width 20–width 40–count 1000000."

Immunuprecipication of Chromatin-Bound Rap1 and Mass Spectrometry

Chromatin extracts were prepared as previously described (van Werven et al., 2008). In short, cells were disrupted using 0.5 mm glass beads in nuclear isolation buffer (NIB: 250 mM sucrose, 10 mM $MgCl_2$, 20 mM HEPES (pH 7.8), 0.1% v/v Triton X-100, 5 mM β-mercaptoethanol, 1X cOmplete protease inhibitor (Roche)). The pellet was then collected by centrifugation (27000 x *g*, 15 min, 4°C), washed once in NIB buffer, centrifuged again, and resuspended in 4.5 mL NIB buffer with 2 mM $CaCl_2$. Samples were treated with 3000 U of micrococal nuclease (MNase, NEB) for 4 min at 30°C and reactions were stopped by addition of EDTA to 10 mM, then transferred onto ice. The concentration of NaCl was adjusted to 150 mM and samples were clarified by centrifugation at 16000 x *g* for 10 min at 4°C. The supernatant was taken as the chromatin extract.

Anti-V5 tag immunoprecipitation was performed on approximately 15 mg of chromatin extract from cells expressing full-length Rap1-V5 (FL, FW5420), Rap1(ΔAD, FW5424)-V5, Rap1(Δ631-696, FW5396)-V5, or containing an empty vector control (Untagged, FW5399). 100 μL of anti-V5 agarose affinity gel antibody (Sigma-Aldrich) was incubated with chromatin extracts for 4 hr at 4°C. Agarose beads were washed 5 times with 1 mL NIB wash buffer (NIB buffer with 350 mM NaCl) and proteins were eluted in SDS-PAGE sample buffer by heating at 95°C for 5 min.

Eluted proteins were subjected to SDS-PAGE, migrated approximately 1 cm into the gel (12% NuPAGE, Invitrogen), and stained with InstantBlue Protein Stain (Expedeon). Proteins were in-gel digested using trypsin, and peptides were analyzed with an Orbitrap-Fusion Lumos mass spectrometer coupled to an Ultimate3000 HPLC equipped with an EASY-Spray nanosource (Thermo Fisher Scientific). Label-free quantification (LFQ) was performed using MaxQuant software (v1.6.01) (Cox and Mann, 2008). Perseus software (version 1.4.0.2) was used for further statistical processing of the proteingroup.txt output table (Tyanova et al., 2016). LFQ intensities were $\log_2$ transformed, and the dataset was filtered for proteins having at least three values in at least one group (each group consisting of triplicate injections). The remaining missing values were imputed using default Perseus settings by drawing from a simulated noise distribution with a down shift of 1.8 and a width of 0.3 compared with the $\log_2$ LFQ intensity distribution. Two-sample t tests were performed with a permutation-based FDR set at 0.05. Proteins that were enriched >2-fold with $p < 0.05$ (comparing Full-Length versus Untagged Control samples) were subjected to SGD Gene Ontology Slim Mapper Process Analysis (https://www.yeastgenome.org/cgi-bin/GO/goSlimMapper.pl). Volcano plots were generated using Graphpad Prism (version 7.02). The processed mass spectrometry data are available in Table S2.

For the GO-Slim terms in Figure 6C, proteins that were enriched >2-fold with $p < 0.05$ (unpaired two-sample t test) were used for the analysis (n = 289 proteins) in the SGD Yeast GO-Slim Process Mapper. 13 proteins that were ambiguously assigned to genes were excluded from the analysis.

Oligonucleotides Used in This Study

A table of oligonucleotides used in this study is available in Table S6.

QUANTIFICATION AND STATISTICAL ANALYSIS

Details of statistical tests used, sample number, and number of independent experiments are included in the relevant figure legends. p values were calculated using the Students' t test, with $p < 0.05$ considered significant. Error bars are described in individual figure legends as ± standard error of the mean (±SEM) or 95% confidence intervals. Standard box-and-whisker plots were generated showing the median value (horizontal line), lower and upper quartiles (lower and upper hinges), and lowest and highest values (whiskers, within 1.5 times interquartile range).

DATA AND SOFTWARE AVAILABILITY

The accession number for the RNA sequencing and TSS sequencing data reported in this paper is GEO: GSE110004.

Molecular Cell, Volume *72*

Supplemental Information

Repression of Divergent Noncoding Transcription

by a Sequence-Specific Transcription Factor

Andrew C.K. Wu, Harshil Patel, Minghao Chia, Fabien Moretto, David Frith, Ambrosius P. Snijders, and Folkert J. van Werven

Figure S1. Wu *et al.*

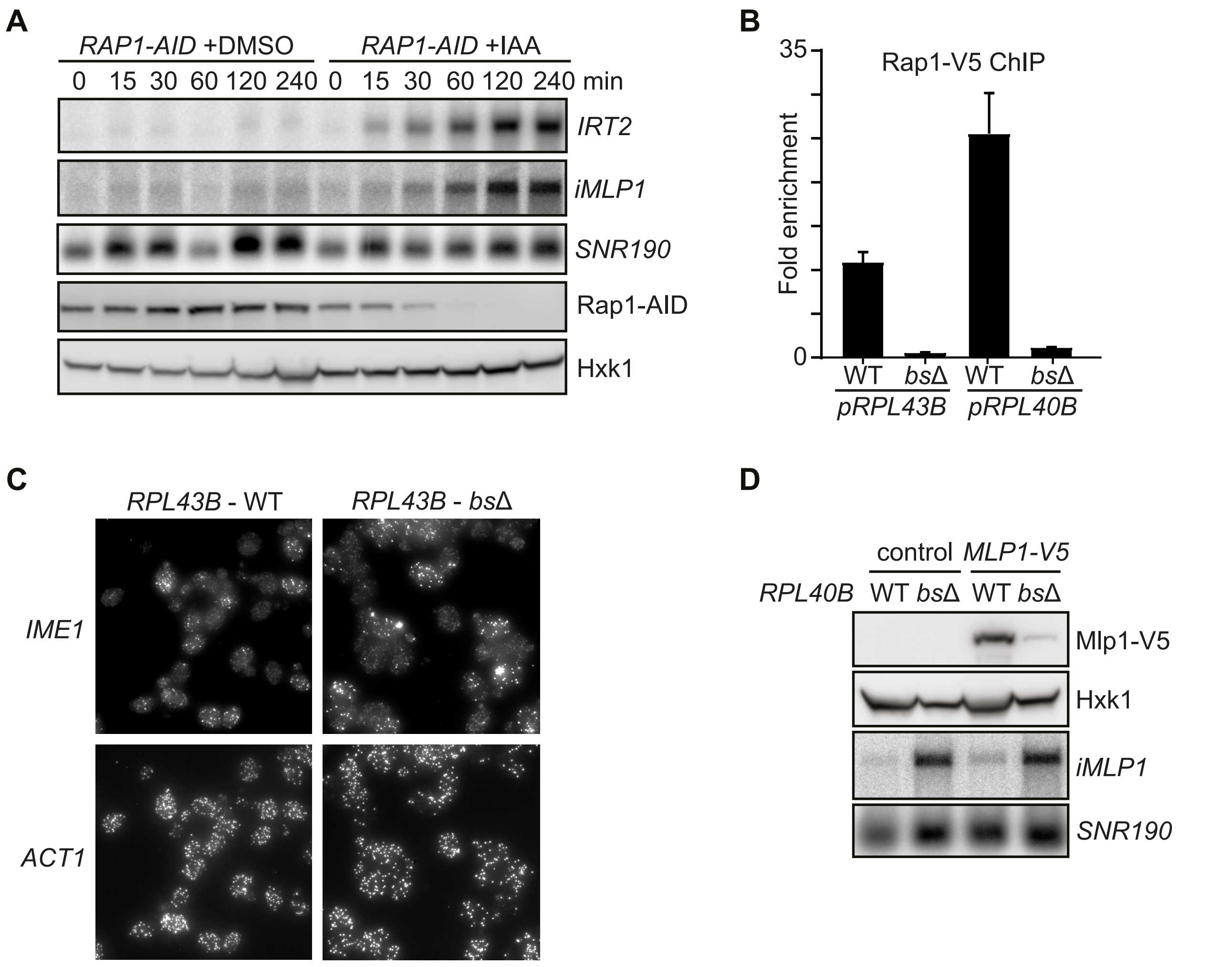

Figure S1. Rap1 prevents expression of noncoding RNAs, Related to Figure 1. (**A**) Northern blots and western blots corresponding to Figure 1D, except samples were included from *RAP1-AID* cells (FW3877) treated with DMSO or IAA at equivalent time points. *IRT2*, *iMLP1*, and *SNR190* transcripts were detected using labelled northern blot probes as described in Figure 1D, and Rap1-V5-AID and Hxk1 were detected using anti-V5 and anti-Hxk antibodies. (**B**) Rap1 binding at *RPL43B* and *RPL40B* promoters determined by chromatin immunoprecipitation. Cells harbouring Rap1-V5 (FW4732), *RPL43B*-bsΔ Rap1-V5 (FW4734), and *RPL40B*-bsΔ Rap1-V5 (FW6228) were grown to exponential phase. Cell were crosslinked with formaldehyde, chromatin extracts were prepared, and anti-V5 antibodies were used to immunoprecipitate Rap1-V5 bound DNA fragments. Rap1 binding at *RPL43B* and *RPL40B* promoters was measured by qPCR, and the signals were normalized over *ACT1* gene 3' end. The mean fold enrichment from three independent experiments plus the standard error of the mean (+SEM) is plotted. (**C**) Representative single-molecule RNA fluorescence in-situ hybridization (RNA FISH) images corresponding to Figure 1H. Single spots corresponding to individual *IME1* (AF594) or *ACT1* (Cy5) mRNA transcripts were counted in diploid wild-type (FW631) or *RPL43B*-bsΔ (FW6139) cells immediately after shifting to SPO medium. (**D**) Northern blots and western blots corresponding to Figure 1D, showing the induction of *iMLP1* and reduction of Mlp1 expression when the *RPL40B* promoter Rap1 site is deleted. Wild-type (FW629), *RPL40B*-bsΔ (FW4141), or *MLP1* tagged with V5 epitope tag (FW4122) and *MLP1-V5 RPL40B*-bsΔ (FW4120) cells were grown to exponential growth and samples were collected. Expression of Hxk1 and *SNR190* are shown for western and northern blot loading controls, respectively.

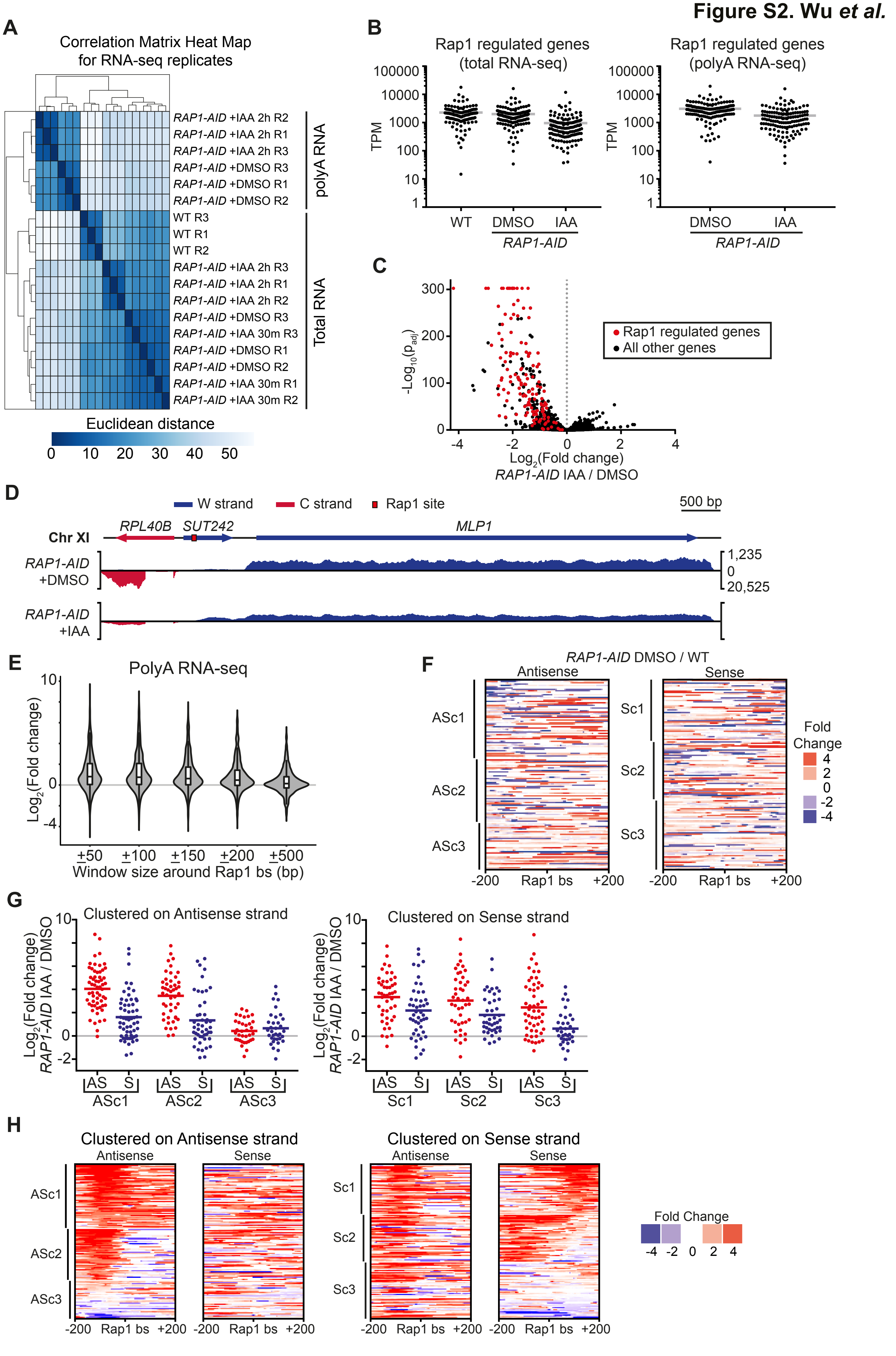
A
Correlation Matrix Heat Map for RNA-seq replicates
RAP1-AID +IAA 2h R2
RAP1-AID +IAA 2h R1
RAP1-AID +IAA 2h R3
RAP1-AID +DMSO R3
RAP1-AID +DMSO R1
RAP1-AID +DMSO R2
WT R3
WT R1
WT R2
RAP1-AID +IAA 2h R3
RAP1-AID +IAA 2h R1
RAP1-AID +IAA 2h R2
RAP1-AID +DMSO R3
RAP1-AID +IAA 30m R3
RAP1-AID +DMSO R1
RAP1-AID +DMSO R2
RAP1-AID +IAA 30m R1
RAP1-AID +IAA 30m R2
polyA RNA
Total RNA
Euclidean distance
0 10 20 30 40 50
B
Rap1 regulated genes (total RNA-seq)
Rap1 regulated genes (polyA RNA-seq)
TPM
100000 10000 1000 100 10 1
WT DMSO IAA
RAP1-AID
DMSO IAA
RAP1-AID
C
-Log10(padj)
300 200 100 0
Rap1 regulated genes
All other genes
-4 -2 0 2 4
Log2(Fold change)
RAP1-AID IAA / DMSO
D
W strand
C strand
Rap1 site
500 bp
Chr XI
RPL40B
SUT242
MLP1
RAP1-AID +DMSO
RAP1-AID +IAA
1,235
0
20,525
E
PolyA RNA-seq
Log2(Fold change)
10 0 -4
±50 ±100 ±150 ±200 ±500
Window size around Rap1 bs (bp)
F
RAP1-AID DMSO / WT
Antisense
Sense
ASc1
ASc2
ASc3
Sc1
Sc2
Sc3
-200 Rap1 bs +200
Fold Change
4 2 0 -2 -4
G
Clustered on Antisense strand
Clustered on Sense strand
Log2(Fold change) RAP1-AID IAA / DMSO
10 0 -2
AS S
ASc1 ASc2 ASc3
Sc1 Sc2 Sc3
H
Clustered on Antisense strand
Clustered on Sense strand
Antisense
Sense
ASc1
ASc2
ASc3
Sc1
Sc2
Sc3
-200 Rap1 bs +200
Fold Change
-4 -2 0 2 4

Figure S2. Rap1 represses divergent noncoding transcription, Related to Figure 2.
(A) Correlation matrix heat map for RNA-seq biological replicate samples, showing the Euclidean distance between the samples across the genome (based on all genes). **(B)** Scatter plots showing distribution of expression for Rap1 regulated genes (n = 141) in wild-type (FW629), and *RAP1-AID* (FW3877) cells after DMSO or IAA treatment. Separate plots shown for total (rRNA depleted) and polyadenylated (polyA) RNA-seq. Each dot represents transcripts per million (TPM) for each gene, shown on the y-axis (exponential scale). Gray lines indicate mean values for each group. **(C)** Volcano plot showing that the expression of Rap1-regulated genes is decreased upon Rap1 depletion. On the y-axis the false discovery rate adjusted *p*-value ($-Log_{10}(p_{adj})$) is plotted, and on the x-axis the fold change is displayed (Log_2(Fold change)). *RAP1-AID* (FW3877) cells after IAA or DMSO treatment were compared. For the analyses (n = 7126) genes were used and in red (n = 141) Rap1 regulated genes are highlighted. Data are calculated from three independent experiments. **(D)** Genome browser tracks showing example of divergent noncoding RNA *iMLP1* repressed by Rap1, adjacent to the *RPL40B* locus. *RAP1-AID* cells (FW3877) were grown to exponential phase and were treated with DMSO or IAA for 2 hours. Samples were taken and processed for total RNA-seq. The normalized reads are shown on the y-axis for the Watson (W, blue) and Crick (C, red) strands. **(E)** Violin and box-and-whisker plots, as described in Figure 2C, showing distribution of expression changes around Rap1 sites (n = 564) for polyadenylated (polyA) transcripts. The fold change in expression comparing *RAP1-AID* +IAA versus *RAP1-AID* +DMSO treated cells is plotted on the y-axis. Signals for Watson and Crick strands were computed separately, resulting in n = 1128 data points for window sizes of 50, 100, 150, 200, and 500 bp up- and downstream of the Rap1 binding site. **(F)** Control heat maps showing the changes in RNA expression on the antisense and sense strands around n = 141 promoter Rap1 sites. For the analysis *RAP1-AID* (FW3877) DMSO treated cells were compared to wild type control (WT, FW629) cells. Promoters were clustered and ordered as described in Figure 2F. **(G)** Scatter plots showing RNA expression changes 100 bp up- and downstream of Rap1 binding sites ($\pm$100 bp) after Rap1 depletion as described in Figure 2E. Data are separated by antisense (left) or sense (right) clusters. Fold change values were calculated for *RAP1-AID* (FW3877) +IAA or +DMSO treated cells from three independent experiments. Number of promoters in each cluster: ASc1 (n = 59), ASc2 (n = 47), ASc3 (n = 35), Sc1 (n = 46), Sc2 (n = 43), Sc3 (n = 52). Horizontal lines, mean values. **(H)** Heat maps showing the changes in RNA expression on the antisense and sense strands, similar to Fig. 2F except that both strands are shown. Clustered and ordered based on antisense (ASc1-3) or sense (Sc1-3) strand signals using k-means clustering (k = 3).

Figure S3. Wu *et al.*

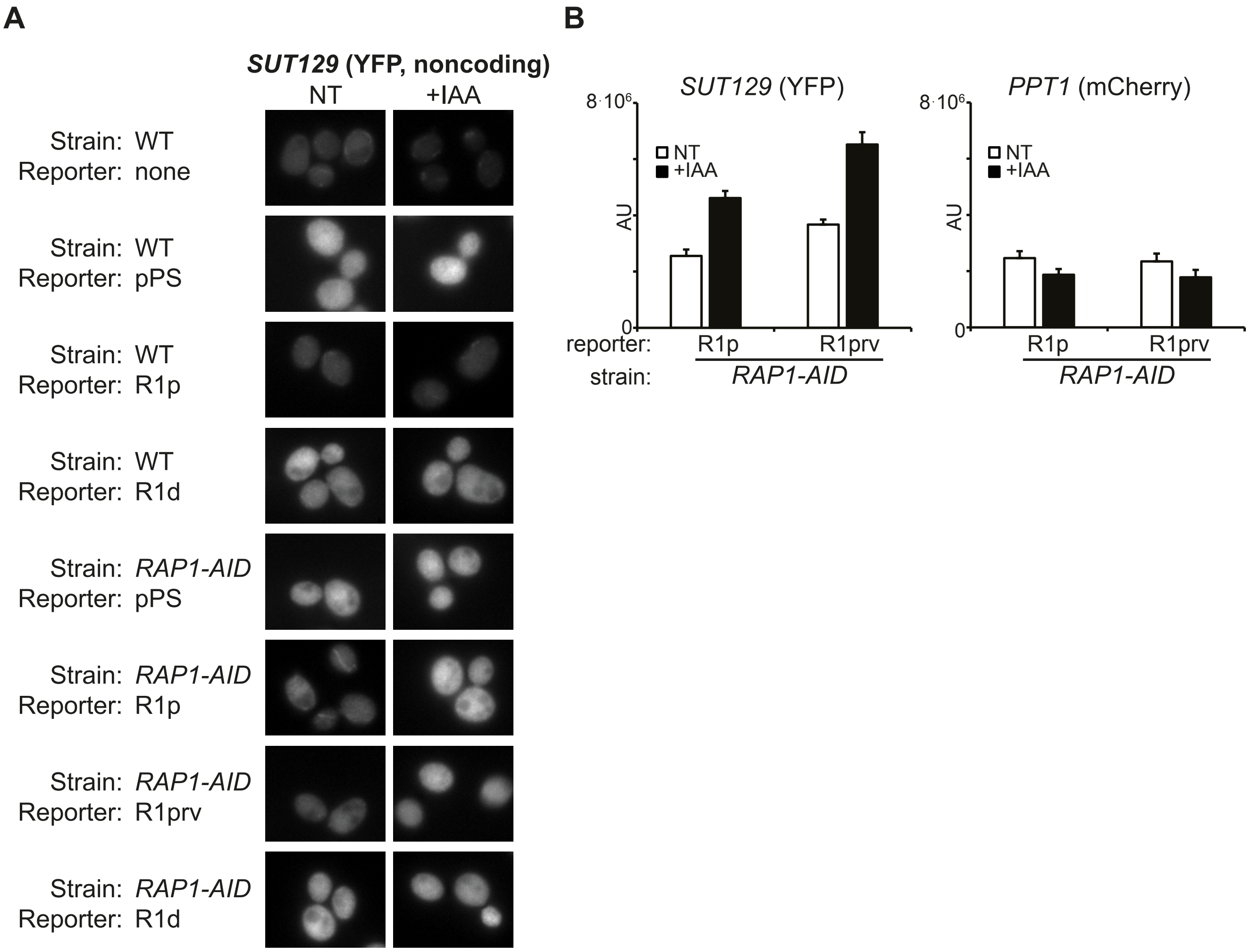

Figure S3. A proximal Rap1 motif is required and sufficient to repress divergent transcription, Related to Figure 3.

(A) Representative images showing *SUT129* promoter activity (YFP, noncoding), as described in Figure 3. Cells were grown in YPD and samples were either not treated (NT) or treated with 500 µM IAA (+IAA) for four hours. The following cells were fixed and imaged: Wild-type cells harbouring no reporter (FW629), control reporter (pPS, FW6407), reporter with proximal Rap1 motifs (R1p, FW6895), reporter with distal Rap1 motifs (R1d, FW7253), *RAP1-AID* cells harbouring control reporter (pPS, FW6208), reporter with proximal Rap1 motifs (R1p, FW6206), reporter with proximal Rap1 motifs in reverse orientation (R1prv, FW6204), reporter with distal Rap1 motifs (R1d, FW6408) **(B)** Orientation of the Rap1 motifs does not affect repression of divergent noncoding transcription. *RAP1-AID* cells harbouring the reporter with R1p (FW6206), or R1prv (FW6204) were quantified for *SUT129* (YFP, noncoding) and *PPT1* (mCherry, coding) promoter activity. Mean signals corrected for background (AU, arbitrary units) are plotted plus 95% confidence intervals (error bars). N = 50 cells were quantified per sample.

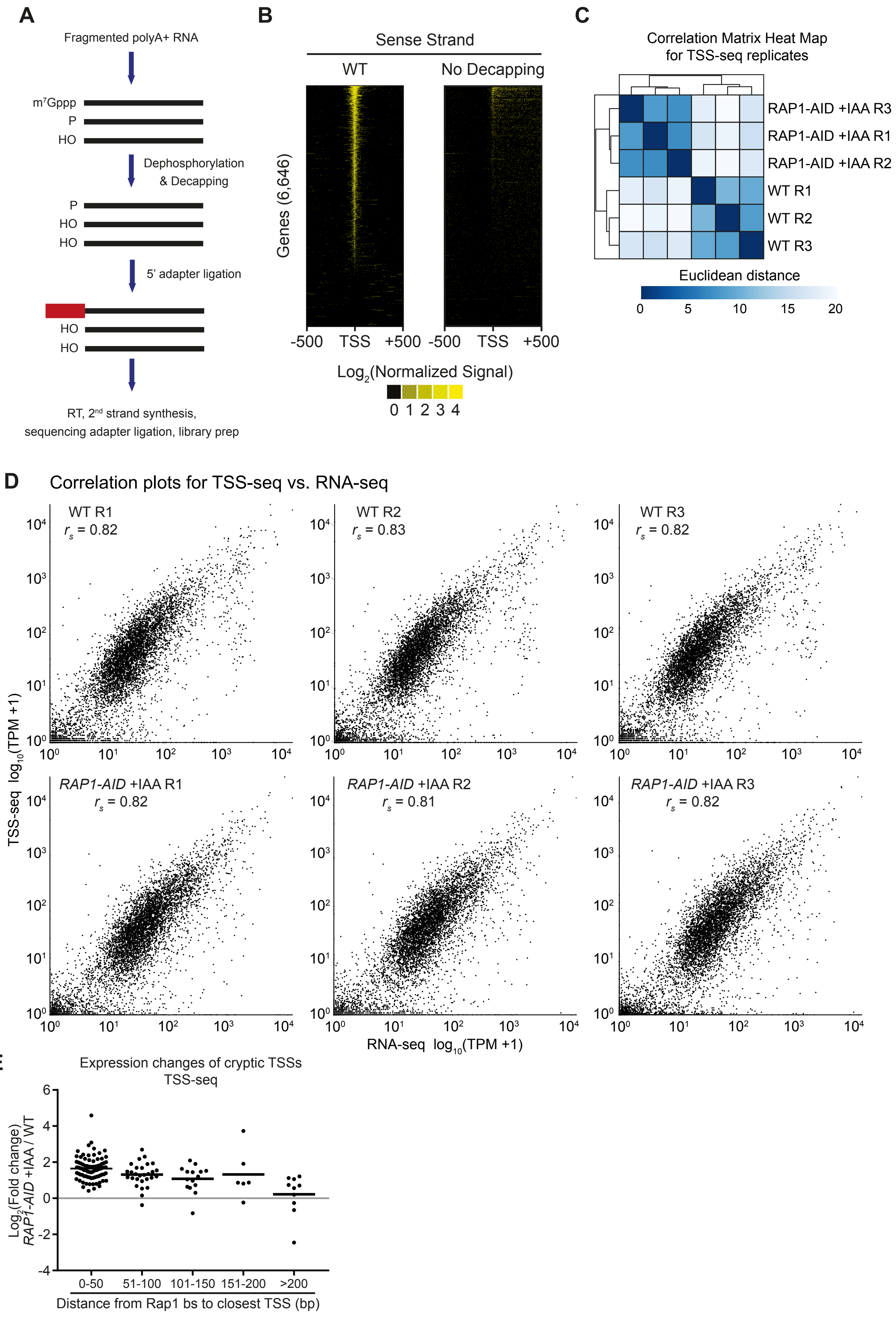
A
Fragmented polyA+ RNA
m7Gppp
P
HO
Dephosphorylation & Decapping
P
HO
HO
5' adapter ligation
HO
HO
RT, 2nd strand synthesis, sequencing adapter ligation, library prep
B
Sense Strand
WT
No Decapping
Genes (6,646)
-500 TSS +500 -500 TSS +500
Log2(Normalized Signal)
0 1 2 3 4
C
Correlation Matrix Heat Map for TSS-seq replicates
RAP1-AID +IAA R3
RAP1-AID +IAA R1
RAP1-AID +IAA R2
WT R1
WT R2
WT R3
Euclidean distance
0 5 10 15 20
D
Correlation plots for TSS-seq vs. RNA-seq
WT R1 rs = 0.82
WT R2 rs = 0.83
WT R3 rs = 0.82
RAP1-AID +IAA R1 rs = 0.82
RAP1-AID +IAA R2 rs = 0.81
RAP1-AID +IAA R3 rs = 0.82
TSS-seq log10(TPM +1)
RNA-seq log10(TPM +1)
E
Expression changes of cryptic TSSs
TSS-seq
Log2(Fold change) RAP1-AID +IAA / WT
0-50 51-100 101-150 151-200 >200
Distance from Rap1 bs to closest TSS (bp)

Figure S4. Rap1 represses divergent transcription initiation near its binding site, Related to Figure 4.

(A) Diagram of TSS sequencing (TSS-seq) protocol. Polyadenylated RNA was isolated and subjected to fragmentation, dephosphorylation, and decapping. A custom adapter sequence was ligated to 5' uncapped fragment ends. Samples then underwent reverse transcription, second strand synthesis, and library preparation for Illumina sequencing. See STAR methods for details. **(B)** Heat map of normalized TSS-seq signals for 6,646 *S. cerevisiae* genes in wild-type (FW629) cells (left), aligned to annotated transcription start sites (TSSs). A colour scale displays the range of normalized signals (Log_2 scale). Normalized counts from 3 independent experiments were merged for the plot. A "no decapping" control sample is shown where samples were not treated with decapping enzyme to enrich for 5' capped transcript ends. **(C)** Correlation matrix heat map for TSS-seq biological replicate samples, showing the Euclidean distance between the samples across the genome. Correlations are based on $\pm$75 bp windows centered on annotated TSSs for all genes (Park et al., 2014). **(D)** Scatter plots showing correlation between TSS-seq and RNA-seq data. TSS-seq counts were obtained from each gene by quantifying the abundance of reads with the 1st transcribed 5' nucleotide within $\pm$75 bp of annotated TSSs (Park et al., 2014), on the respective strand. These were converted to TPM values and plotted (TSS-seq, y-axis) against RNA-seq TPM values (x-axis) for the corresponding gene. Individual replicate comparisons are plotted separately. r_S, spearman's correlation coefficient. **(E)** Scatter plots showing changes in expression of cryptic TSSs near promoter Rap1 sites, comparing *RAP1-AID* +IAA versus wild-type control samples. TSSs were classified into bins of 50 bp, increasing in distance to the promoter Rap1 binding site. Fold change values were calculated from three independent experiments. Horizontal lines, mean values.

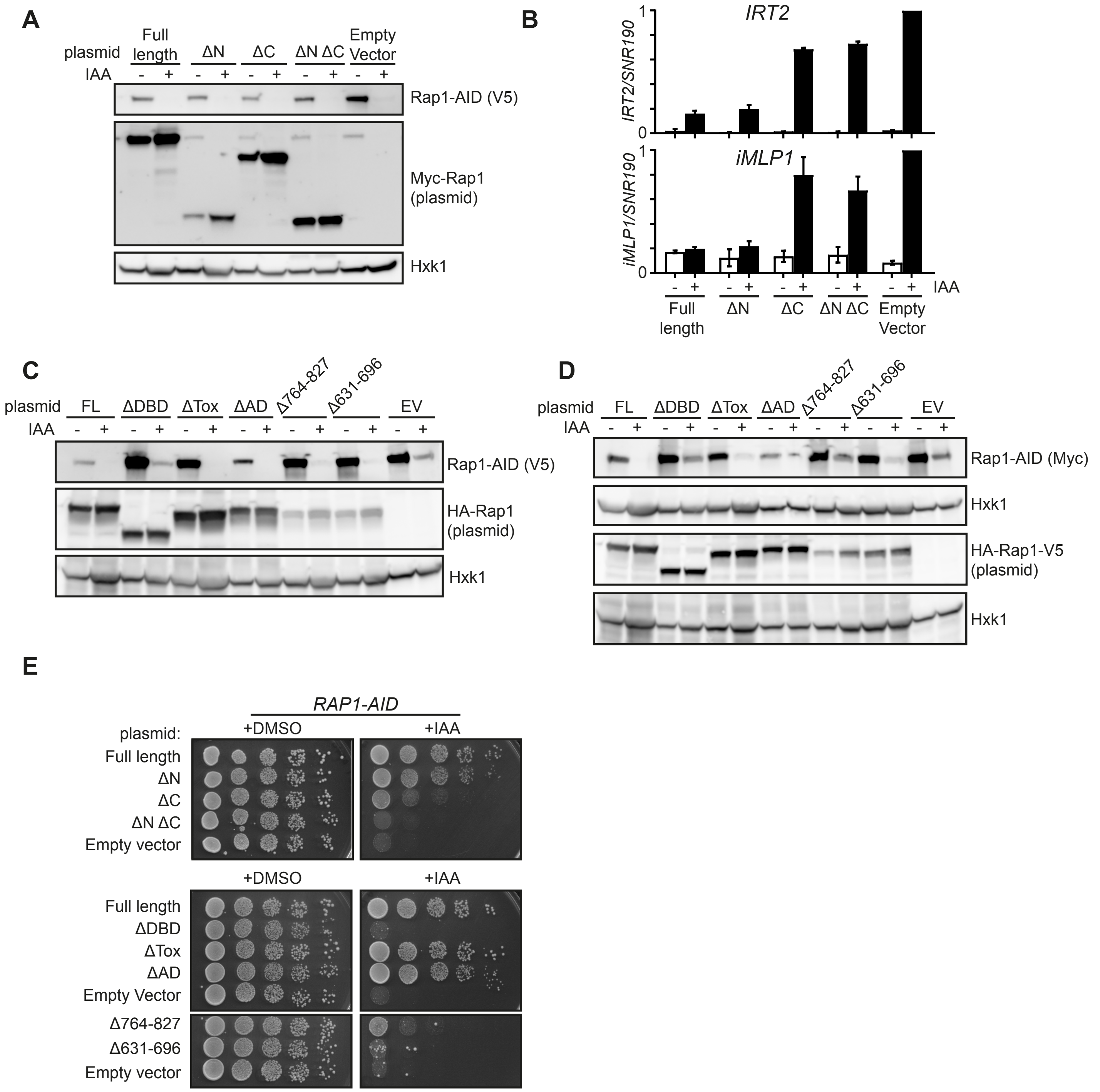
A
plasmid
Full length
ΔN
ΔC
ΔN ΔC
Empty Vector
IAA
Rap1-AID (V5)
Myc-Rap1 (plasmid)
Hxk1
B
IRT2
IRT2/SNR190
iMLP1
iMLP1/SNR190
IAA
Full length
ΔN
ΔC
ΔN ΔC
Empty Vector
C
plasmid
FL
ΔDBD
ΔTox
ΔAD
Δ764-827
Δ631-696
EV
IAA
Rap1-AID (V5)
HA-Rap1 (plasmid)
Hxk1
D
plasmid
FL
ΔDBD
ΔTox
ΔAD
Δ764-827
Δ631-696
EV
IAA
Rap1-AID (Myc)
Hxk1
HA-Rap1-V5 (plasmid)
Hxk1
E
RAP1-AID
plasmid:
+DMSO
+IAA
Full length
ΔN
ΔC
ΔN ΔC
Empty vector
+DMSO
+IAA
Full length
ΔDBD
ΔTox
ΔAD
Empty Vector
Δ764-827
Δ631-696
Empty vector

Figure S5. The Rap1 C-terminal domain contributes to repression of divergent noncoding transcription, Related to Figure 5.

(A) Expression of Rap1 truncation mutants, corresponding to Figure 5B. *RAP1-AID* cells expressing single copy integration vectors harboring full-length (FL) (FW5129), N-terminal deletion (ΔN) (FW5133), C-terminal deletion (ΔC) (FW5138), and ΔN ΔC (FW5141) Rap1*,* or empty vector (EV, FW5145). Samples were collected from cells before and 2 hours after treatment with IAA. AID-tagged Rap1 protein, Myc-tagged truncated Rap1 proteins, and Hxk1 (loading control) were detected using anti-V5, Myc, and Hxk antibodies, respectively. **(B)** Quantification of *IRT2* and *iMLP1* expression described in Figure 5B. Samples from two independent experiments were processed and the mean values plus standard error of the mean are displayed (±SEM). Signals for *IRT2* and *iMLP1* were normalized over *SNR190*, and to control for technical variation between experiments and blots, the normalized signal for Rap1-V5-AID +IAA containing empty vector was set to 1. **(C)** Expression of Rap1 domain mutants described in Figure 5C. *RAP1-AID* cells expressing single copy integration vectors harboring full-length Rap1 (FL) (FW4948), DNA-binding domain deletion (ΔDBD) (FW4950), toxicity domain deletion (ΔTox) (FW4952), activation domain deletion ΔAD (FW4954), residues 764 to 827 deleted (Δ764-827) (FW4958), residues 631 to 696 deleted (Δ631-696) (FW4960), or empty vector (EV) (FW5145). Samples were collected 0 and 2 hours after treatment with IAA. AID-tagged Rap1 protein, HA-tagged Rap1 domain mutant proteins, and Hxk1 (loading control) were detected using anti-V5, HA, and Hxk1 antibodies, respectively. **(D)** Similar to C, except that endogenous Rap1 was tagged with AID-Myc and the Rap1 domain mutant constructs also contain C-terminal V5 epitope tags, as described in Figure 5D. **(E)** Spot growth assay of Rap1 truncation and domain deletion constructs described in A and C. Cells were grown to saturation in YPD media overnight, then adjusted to equivalent optical density (OD_{600} = 0.4). Serial 5-fold dilutions were spotted onto YPD agar plates with IAA or DMSO. Plates were incubated at 30 °C for 2 days before imaging.

Figure S6. Wu *et al.*

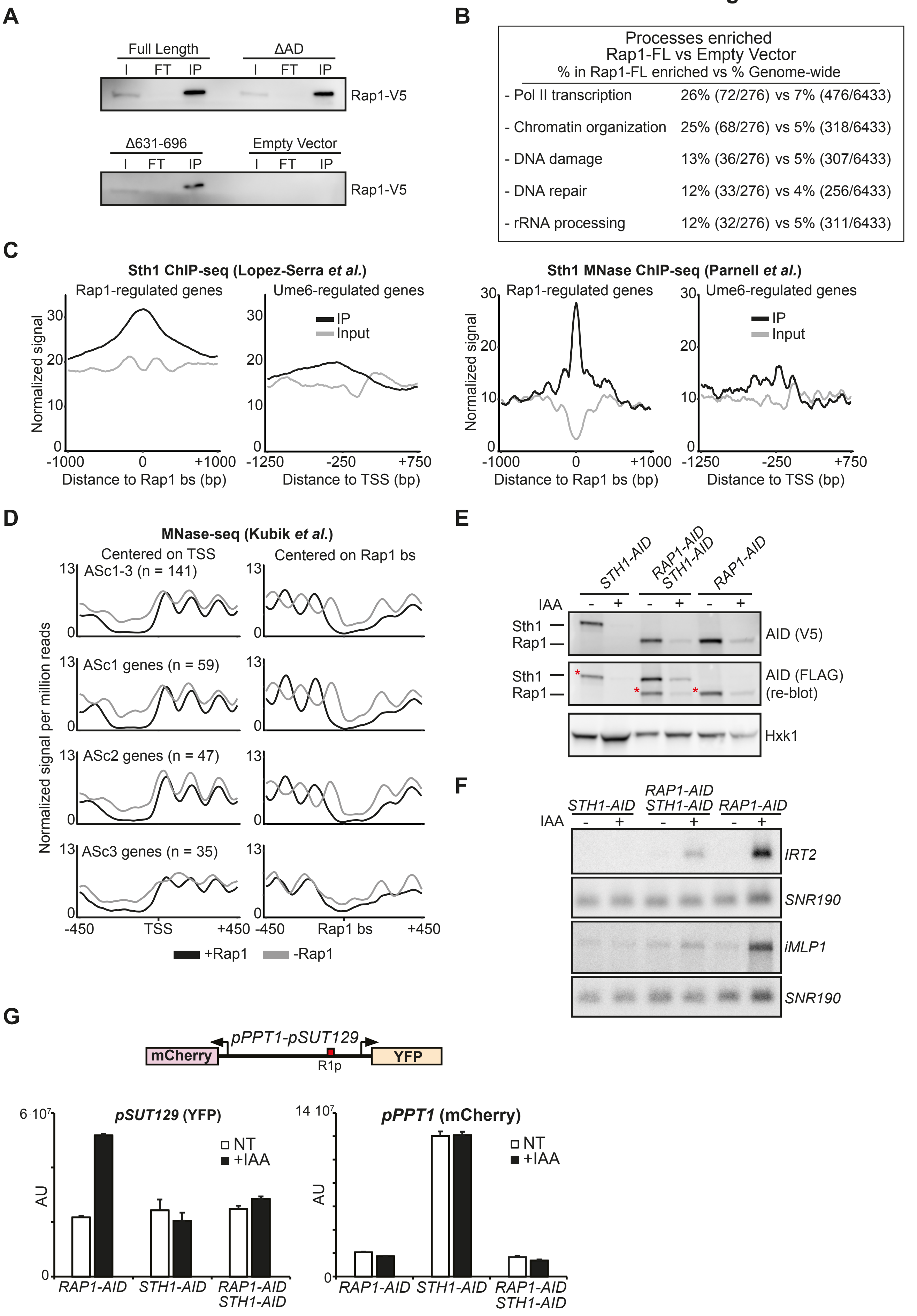

Figure S6. Co-depletion of RSC with Rap1 suppresses divergent transcription, Related to Figure 6.

(A) Expression of V5-tagged Rap1 domain mutant proteins detected after immunoprecipitation from MNase-treated chromatin extracts as described in Figure 6A. 0.67% of input (I), 0.67% of flow-through (FT), and 10% of immunoprecipitated sample (IP) eluted from anti-V5 beads was used for western blot. Rap1 expression was detected with anti-V5 antibody. Samples were collected from strains containing single copy integration vectors either expressing V5-tagged Rap1: FL (FW5420), ΔAD (FW5424), Δ631-696 (FW5396) or an empty vector (FW5399). **(B)** Expanded Yeast GO-Slim Process analysis of proteins enriched with chromatin bound Rap1, as described in Figure 6C. The GO-Slim terms and their frequencies of proteins enriched in Rap1-FL vs empty vector control and genome-wide are shown. 13 proteins with ambiguous gene assignment were excluded from the analysis. **(C)** Metagene plots of Sth1 ChIP-seq (left) and Sth1 MNase ChIP-seq (right) data at Rap1-regulated promoters. The normalized signal per million reads is plotted on the y-axis, 1 kb up- and downstream of the promoter Rap1 binding sites (n = 141). As control, we generated metagene plots for Ume6-regulated gene promoters (n = 87). Signals from immunoprecipitated (black) and input (gray) samples are shown. For Sth1 MNase ChIP-seq, chromatin was liberated by MNase digestion prior to immunoprecipitation. Data were obtained from GEO (GSE56994 & GSE65594) (Lopez-Serra et al., 2014; Parnell et al., 2015). **(D)** MNase-seq metagene plots showing that promoters with Rap1-dependent divergent transcription show differences in nucleosome occupancy. Normalized signal per million reads is shown 450 bp up- and downstream of the transcription start site (TSS, left panel), or Rap1 binding site (Rap1 bs, right panel). Separate plots are shown for the set of Rap1-regulated genes as shown in Figure 2F (n = 141), and each cluster of genes according to the antisense strand (ASc1, ASc2, ASc3). Nucleosome positions before (black) and after (gray) Rap1 depletion are shown for each plot. Data were obtained from GEO (GSE73337) (Kubik et al., 2015). **(E)** Auxin-induced depletion (AID) of Rap1 and Sth1 detected by western blotting. Samples were collected from *RAP1-AID* (FW3877), *STH1-AID* (FW6032), and *RAP1-AID STH1-AID* (FW6231) cells as in Figure 6F, before (-IAA) and after (+IAA) auxin treatment. Depletion of V5-AID tagged proteins was detected with an anti-V5 antibody. Sth1-AID-FLAG was detected with an anti-FLAG antibody after re-probing of the V5-blot. The asterisks on the FLAG blot indicate the residual V5 blot signal. Hxk1 was detected as a loading control. **(F)** *IRT2* and *iMLP1* expression in cells co-depleted for Sth1 and Rap1. Same data as described in Figure 6F, except that the original order of loading for the samples is displayed. **(G)** Reporter assay showing that co-depletion of RSC and Rap1 suppresses noncoding transcription. Quantification of *SUT129* (YFP) and *PPT1* (mCherry) promoter activity as described in Figure 6H. Cells harbouring *RAP1-AID* (FW6206), *STH1-AID* (FW6218), and *RAP1-AID STH1-AID* (FW6433) were either not treated (NT) or treated with IAA (+IAA). Mean signals corrected for background (AU, arbitrary units) are plotted. The error bars represent 95% confidence intervals (n = 50 cells).

Figure S7. Wu *et al.*

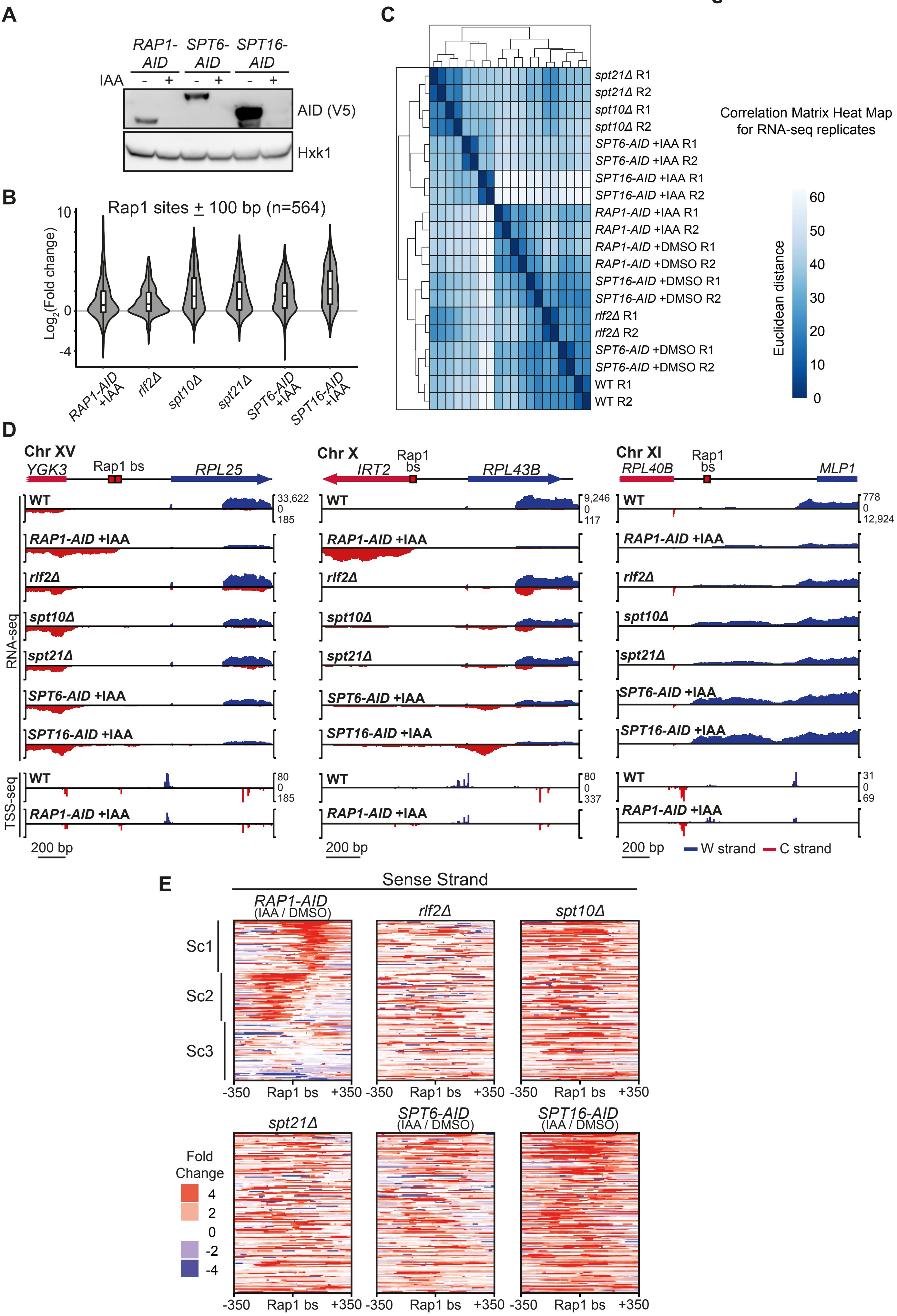

Figure S7. Rap1 suppresses divergent transcription independent of chromatin assembly and remodelling pathways, Related to Figure 7.
(A) Depletion of Rap1, Spt6, and Spt16 detected by western blotting. *RAP1-AID* (FW3877), *SPT6-AID* (FW5555), and *SPT16-AID* (FW5559) cells were either mock-treated with DMSO (-) or treated with IAA (+) as in Figure 7A. AID-tagged proteins were detected with an anti-V5 antibody. Hxk1 was used as a loading control. **(B)** Violin and box-and-whisker plots showing the distribution of changes in RNA expression around Rap1 sites. Fold change values of *rlf2Δ* (FW5609), *spt10Δ* (FW5543), and *spt21Δ* (FW5547) compared to a wild-type control (WT, FW629) are displayed, whereas for *RAP1-AID* (FW3877), *SPT6-AID* (FW5555), and *SPT16-AID* (FW5559) fold change values were obtained by comparing IAA treatment (+IAA) to mock treatment (+DMSO). The distribution of RNA expression changes for ±100 bp windows (n = 1128) around Rap1 binding sites (n = 564) is shown on the y-axis, calculated from two independent experiments. **(C)** Correlation matrix heat map for total RNA-seq biological replicate samples, showing the Euclidean distance between the samples across the genome (based on all genes). **(D)** Divergent transcription at the *RPL25, RPL43B*, and *RPL40B* loci, comparing Rap1-repressed noncoding transcription to chromatin regulators (*RLF2*, *SPT10*, *SPT21*, *SPT6*, and *SPT16*). RNA-seq data of strains described in A and B. The normalized reads are shown on the y-axis for the Watson (W, blue) and Crick (C, red) strands. Rap1 binding sites are shown as red boxes. The TSS-seq tracks for wild-type (WT) and Rap1 depletion (*RAP1-AID* +IAA) are displayed in the last two tracks. **(E)** Heat maps showing the changes in RNA expression as described for Figure 7C, except that data from sense strand was used for the analysis. A colour scale displays the fold change values.

Table S1. Summary of Rap1 silencing mutant screen for *IRT2* expression, Related to Figure 5

		IRT2	**HM silencing**	**telomere silencing**	**telomere length**
**plasmid**	**mutant**		**(Feeser and Wolberger, PMID 18538788)**		
372	EV	+++	NA	NA	NA
501	Δ672-827	+	loss	loss	increase
540	T700A D701A R747A K748A N749A Patch 2A	-	wt	wt	increase
543	D761A M763A M817A Patch 5A	-	wt	decrease	increase
544	D761A M763A R814A M817A Patch 5B	-	wt	decrease	increase
493	R747A	-	wt	wt	increase
495	M817A	-	wt	decrease	increase
497	R804A M817A	-	wt	decrease	increase
498	T700A D701A R747A K748A N749A S753A Patch 2B	-	wt	wt	increase
499	S725A D727A E729A Patch 4	-	loss	wt	increase
516	D727A	-	loss	wt	increase
531	M817R	-	wt	decrease	increase
539	N679A I682A N782A Patch 1	-	wt	wt	wt
541	H709A D742A E743A Patch 3A	-	wt	wt	increase
542	L706A H709A D742A E743A Patch 3B	-	wt	wt	increase
545	R747S	-	NA	NA	NA
492	H709A	-	wt	wt	increase
494	M763A	-	wt	decrease	decrease
496	M763A M817A	-	wt	decrease	wt
500	K796A R804A T812A Patch 6	-	wt	wt	increase
510	D689A	-	wt	wt	wt
511	K696A	-	wt	wt	wt
512	D701A	-	wt	wt	wt
513	D701R	-	wt	wt	wt
514	Q715A	-	wt	wt	wt
515	D701A H789A	-	wt	wt	wt
517	E729A	-	wt	wt	wt
518	S731A	-	wt	wt	wt
519	S731Y	-	wt	wt	wt
520	E734A	-	wt	wt	wt
521	E743R	-	wt	wt	wt
522	S753Y	-	wt	wt	wt
523	N782R	-	wt	wt	wt
524	H789A	-	wt	wt	wt
525	D790A	-	wt	wt	wt
526	K796A	-	wt	wt	increase
527	N798A	-	wt	wt	wt
528	Q800A	-	wt	wt	wt
529	E801A	-	wt	wt	wt
530	R804A	-	wt	wt	wt
532	M817Y	-	wt	wt	wt
533	R820A	-	wt	wt	wt
534	D701A R747A	-	wt	wt	increase
535	N798A D799A	-	wt	wt	wt
536	R804A T812A	-	wt	wt	wt
537	S731Y M763A	-	wt	decrease	wt
538	S731Y R820E	-	wt	wt	wt
471	FL control	-	NA	NA	NA

Table S3. Summary of screen for regulators of *IRT2* and *iMLP1* expression, Related to Figure 7

gene	mutant type	strain reference	*IRT2* levels	*iMLP1* levels	mutant type	*IRT2*	*iMLP1*
					(van Bakel *et al.*, PMID 23658529)		
Ada2	deletion	FW6715	-	-	NA	NA	NA
Arp8	deletion	FW6707	-	-	NA	NA	NA
Bre1	deletion	FW6722	-	-	deletion	-	-
Bur2	deletion	FW4817	-	-	NA	NA	NA
Cdc40	deletion	FW6683	-	+	NA	NA	NA
Cmr1	deletion	FW6725	-	-	NA	NA	NA
Ctk1	deletion	FW4756	-	-	NA	NA	NA
Est2	deletion	FW4757	-	-	NA	NA	NA
Gcn4	deletion	FW6682	-	+	NA	NA	NA
Gcn5	deletion	FW6717	-	-	NA	NA	NA
Gcr2	deletion	FW6698	-	-	NA	NA	NA
Hst1	deletion	FW6721	-	-	NA	NA	NA
Hst2	deletion	FW6691	-	-	NA	NA	NA
Hst3	deletion	FW6678	-	+	NA	NA	NA
Hst4	deletion	FW6686	-	-	NA	NA	NA
Ino80	deletion	FW4819	-	-	deletion	-	-
Isw1	deletion	FW6681	-	+	deletion	-	-
Isw2	deletion	FW6679	-	-	deletion	-	-
Mga2	deletion	FW6700	-	-	NA	NA	NA
Ngg1	deletion	FW6687	-	-	NA	NA	NA
NHP6A	deletion	FW6719	-	-	NA	NA	NA
Nrd1	AID	FW4821	-	-	NA	NA	NA
Opi3	deletion	FW6701	-	-	NA	NA	NA
Paf1	deletion	FW6706	-	-	deletion	-	+
Rap1	AID	FW3877	++	++	ts and tet-off	++	++
Rif1	deletion	FW6729	-	+	NA	NA	NA
Rif2	deletion	FW6704	-	-	NA	NA	NA
Rlf2	deletion	FW6703	-	++	deletion	-	+
Rpb9	deletion	FW6694	-	+	NA	NA	NA
Rpd3	deletion	FW6689	-	-	deletion	-	-
Rrd1	deletion	FW6708	-	-	NA	NA	NA
Rrp6	deletion	FW6680	-	-	NA	NA	NA
Rsc1	deletion	FW6685	-	+	NA	NA	NA
Rsc2	deletion	FW6718	-	-	NA	NA	NA
Rtt106	deletion	FW6699	-	+	NA	NA	NA
Rtt109	deletion	FW6677	-	+	NA	NA	NA
Sch9	deletion	FW4820	-	-	NA	NA	NA
Set2	deletion	FW6728	-	-	deletion	-	-
Set3	deletion	FW6709	-	-	NA	NA	NA
Sgf29	deletion	FW6726	-	-	NA	NA	NA
Sgf73	deletion	FW6695	-	-	NA	NA	NA
Sin4	deletion	FW6716	-	-	NA	NA	NA
Sir1	deletion	FW6705	-	-	NA	NA	NA
Sir2	deletion	FW6711	-	-	deletion	-	-
Sir3	deletion	FW6713	-	-	NA	NA	NA

Table S3. Summary of screen for regulators of *IRT2* and *iMLP1* expression, Related to Figure 7

gene	mutant type	strain reference	*IRT2* levels	*iMLP1* levels	mutant type	*IRT2*	*iMLP1*
					**(van Bakel *et al.*, PMID 23658529)**		
Sir4	deletion	FW6690	-	-	NA	NA	NA
Snf2	deletion	FW6724	-	-	deletion	-	-
Snf5	deletion	FW6723	-	-	NA	NA	NA
Spt10	deletion	FW5543	-	++	deletion	-	++
Spt16	AID	FW5559	+	++	NA	+	++
Spt21	deletion	FW6676	-	++	deletion	-	++
Spt23	deletion	FW4758	-	-	NA	NA	NA
Spt3	deletion	FW6684	-	-	NA	NA	NA
Spt4	deletion	FW6710	-	-	NA	NA	NA
Spt6	AID	FW5555	-	+	ts	+	++
Spt7	deletion	FW6714	-	-	NA	NA	NA
Spt8	deletion	FW6675	-	-	NA	NA	NA
Srb2	deletion	FW6702	-	-	NA	NA	NA
Ssn3	deletion	FW6693	-	-	NA	NA	NA
Sum1	deletion	FW6696	-	-	NA	NA	NA
Swi3	deletion	FW6688	-	-	NA	NA	NA
Swr1	deletion	FW6697	-	-	deletion	-	-
Trf4	deletion	FW6720	-	-	NA	NA	NA
Ubp3	deletion	FW6712	-	-	NA	NA	NA
Vps16	deletion	FW6692	-	-	NA	NA	NA
Xrn1	deletion	FW4759	-	-	NA	NA	NA

Table S5. Plasmids, Related to STAR Methods

plasmid number	plasmid name
255	*pFA6A-V5::KanMX6*
252	*pFA6A-V5-IAA7::KanMX6*
547	*pKAN-IAA17 (71-114)-Myc::KanMX*
546	*pHYG-IAA17 (71-114)-FLAG::hphNT*
250	*pNH603 pGPD1-osTIR1 HIS3*
247	*pNH605 pGPD1-osTIR1 LEU2*
227	*NatMX gene deletion*
471	*pNH603 Myc-NLS-Rap1 (1-827)::HIS3*
472	*pNH603 Myc-NLS-Rap1 (339-827)::HIS3*
473	*pNH603 Myc-NLS-Rap1 (1-599)::HIS3*
474	*pNH603 Myc-NLS-Rap1 (339-599)::HIS3*
372	*pNH603::HIS3 single copy integration vector*
477	*pNH603 HA-NLS-Rap1 (1-827)::HIS3*
478	*pNH603 HA-NLS-Rap1 (ΔDBD 362-597)::HIS3*
479	*pNH603 HA-NLS-Rap1 (ΔTox 597-662)::HIS3*
480	*pNH603 HA-NLS-Rap1 (ΔAD 631-678)::HIS3*
482	*pNH603 HA-NLS-Rap1 (Δ764-827)::HIS3*
483	*pNH603 HA-NLS-Rap1 (Δ631-696)::HIS3*
566	*pNH603 HA-NLS-Rap1 (1-827)-V5::HIS3*
558	*pNH603 HA-NLS-Rap1 (ΔDBD 362-597)-V5::HIS3*
559	*pNH603 HA-NLS-Rap1 (ΔTox 597-662)-V5::HIS3*
568	*pNH603 HA-NLS-Rap1 (ΔAD 631-678)-V5::HIS3*
561	*pNH603 HA-NLS-Rap1 (Δ764-827)-V5::HIS3*
562	*pNH603 HA-NLS-Rap1 (Δ631-696)-V5::HIS3*
105	*LoxP-KanMX5-LoxP*
106	*LoxP-HIS5MX4-LoxP*
108	*LoxP-KlURA3MX4-LoxP*
109	*pGAL1-CRE::URA3*
110	*pGAL1-CRE::HIS3*
592	*YFP-pPPT1-mCherry::NatMX6*
593	*YFP-R1p(SspI)-pPPT1-mCherry::NatMX6*
595	*YFP-R1prv(SspI)-pPPT1-mCherry::NatMX6*
618	*YFP-R1d(XmnI)-pPPT1-mCherry::NatMX6*
492-501, 510-545	*pNH603 Myc-NLS-Rap1 (point or patch mutant)::HIS3, see Table S1*

Table S6. Oligonucleotides, Related to STAR Methods

Primer no	Sequence (5' - 3')	Name	Notes
N/A	CACTCTrGrArGrCrArArUrArCrC	TSS-seq RNA adapter	5' RNA adapter oligonucleotide ligated to 5' end of decapped RNA fragments - TSS sequencing protocol.<br>5' RNA adapter ligated as RNA:DNA hybrid to 5' ends of decapped RNA fragments.
N/A	GCAC[iBiodT]GCACTCTGAGCAATACC	TSS-seq 2nd strand synthesis primer	Primer for 2nd strand synthesis in TSS sequencing protocol (internally biotinylated)
489	ATGCAACGCCTACTTGTTTT	IME1 -2400 REV	Oligos to amplify *IRT2* northern blot probe DNA template from genomic DNA
493	GATGGAGGGTTGGCATAAAA	IME1 UME6Δ check FW	Oligos to amplify *IRT2* northern blot probe DNA template from genomic DNA
1130	TGCACCCAGACAACTACACA	AW10_RPL40B_ncRNA_probef	Oligos to amplify *iMLP1* northern blot probe DNA template from genomic DNA
1131	CGCCGTAAGACTCAATGGAC	AW11_RPL40B_ncRNA_prober	Oligos to amplify *iMLP1* northern blot probe DNA template from genomic DNA
2111	GGCCCTGATGATAATG	AW411_SNR190_NBprobe_fwd	Oligos to amplify *SNR190* northern blot probe DNA template from genomic DNA
2112	GGCTCAGATCTGCATG	AW412_SNR190_NBprobe_rev	Oligos to amplify *SNR190* northern blot probe DNA template from genomic DNA
1701	TGCGGCTGGTATGGTATTGTAAGG	AW255_pRPL43B_Rap1_ChIP_A_fwd	Oligos to amplify region adjacent to Rap1 binding sites at *pRPL43B* , for ChIP-qPCR
1702	AAAGGCAGAAGATGGGCGGC	AW256_pRPL43B_Rap1_ChIP_B_rev	Oligos to amplify region adjacent to Rap1 binding sites at *pRPL43B* , for ChIP-qPCR
2170	GCTTTACCTCTTGCTGAACGGGA	AW434_pRPL40B_ChIP_A_fwd	Oligos to amplify region adjacent to Rap1 binding sites at *pRPL40B* , for ChIP-qPCR
2171	TCCGCCATATGATCCGCCTC	AW435_pRPL40B_ChIP_A_rev	Oligos to amplify region adjacent to Rap1 binding sites at *pRPL40B* , for ChIP-qPCR
106	GTACCACCATGTTCCCAGGTATT	FvW_ACTFrt	Oligos to ampify region at 3' end of *ACT1* ORF, for ChIP-qPCR
268	AGATGGACCACTTTCGTCGT	FvW_ACT1rt	Oligos to ampify region at 3' end of *ACT1* ORF, for ChIP-qPCR
N/A	TATGTATGGGTTAAAAAGGATGTATGGATG	FW-ppt_rap1-3top2	Oligos to clone transcription factor binding sites into fluorescent reporter plasmid (blunt-end cloning)
N/A	CATCCATACATCCTTTTTAACCCATACATA	FW-ppt_rap1-3bottom2	Oligos to clone transcription factor binding sites into fluorescent reporter plasmid (blunt-end cloning)
